# 5-Hydroxyindole-Based EZH2 Inhibitors Assembled via TCCA-Catalyzed Condensation and Nenitzescu Reactions

**DOI:** 10.3390/molecules25092059

**Published:** 2020-04-28

**Authors:** Fangyu Du, Qifan Zhou, Wenjiao Sun, Cheng Yang, Chunfu Wu, Lihui Wang, Guoliang Chen

**Affiliations:** 1 Key Laboratory of Structure-Based Drug Design & Discovery of Ministry of Education, School of Pharmaceutical Engineering, Shenyang Pharmaceutical University, No. 103 Wenhua Road, Shenhe District, Shenyang 110016, China; 2 Department of Pharmacology, Shenyang Pharmaceutical University, No. 103 Wenhua Road, Shenhe District, Shenyang 110016, China

**Keywords:** 5-hydroxyindole, EZH2 inhibitors, anti-tumor, trichloroisocyanuric acid

## Abstract

5-Hydroxyindole derivatives have various demonstrated biological activities. Herein, we used 5-hydroxyindole as a synthetic starting point for structural alterations in a combinatorial process to synthesize 22 different compounds with EZH2 inhibitor pharmacophores. A series of 5-hydroxyindole-derived compounds were screened inhibitory activities against K562 cells. According to molecular modeling and in vitro biological activity assays, the preliminary structure-activity relationship was summarized. Compound **L–04** improved both the H3K27Me3 reduction and antiproliferation parameters (IC_50_ = 52.6 μM). These findings revealed that compound **L–04** is worthy of consideration as a lead compound to design more potent EZH2 inhibitors. During the preparation of compounds, we discovered that trichloroisocyanuric acid (TCCA) is a novel catalyst which demonstrates condensation-promoting effects. To gain insight into the reaction, in situ React IR technology was used to confirm the reactivity. Different amines were condensed in high yields with β-diketones or β-ketoesters in the presence of TCCA to afford the corresponding products in a short time (10~20 min), which displayed some advantages and provided an alternative condensation strategy.

## 1. Introduction

**Figure 1:**
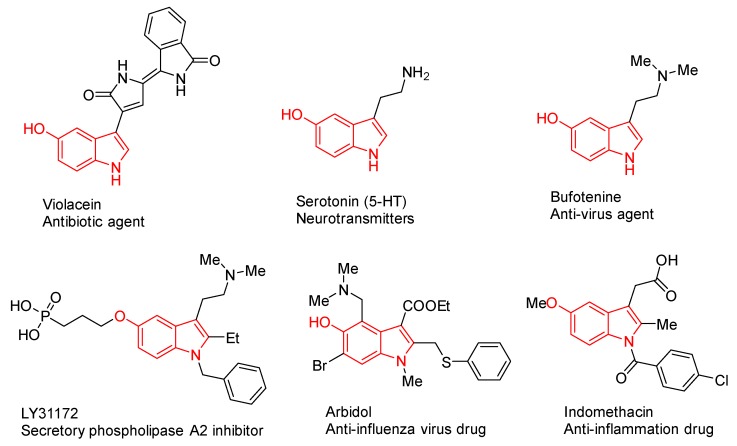
Selected natural products and pharmaceuticals containing 5-hydroxyindole scaffold.

The 5-hydroxyindole scaffold, ubiquitously present in natural products and pharmaceuticals, with demonstrated diverse biological activities [1,2], such as the selective secretory phospholipase A2 inhibitor LY31172 [3], the antibiotic agent violacein [4], the anti-influenza virus drug arbidol [5], the neurotransmitter serotonin [6], the anti-virus agent bufotenine [7], and the anti-inflammatory drug indomethacin [8] (Figure 1). To date, various protocols have been developed for its preparation [9,10,11,12,13], among which the Nenitzescu indole synthesis where 1,4-benzoquinone is condensed with enamines to afford *N*-substituted 5-hydroxyindoles has been proven to be the simplest strategy [14]. The related enamines are ubiquitous reagents in organic synthesis [15,16,17], which are generally obtained by condensation reaction using amines, β-diketones or β-ketoesters as starting materials [18,19,20,21]. 

Our group is devoted to the study and development of histone methyltransferase inhibitors. The enhancer of zeste homolog 2 (EZH2), known as the key catalyzed subunit of the polycomb repressor complex 2 (PRC2), can regulate trimethylated histone H3 lysine 27 (H3K27Me3) on chromatin, and subsequently silence tumor suppressor genes [22]. It is reported that an abnormally high expression of EZH2 was discovered in various malignant cells and other diverse biological processes [23]. In recent years, several EZH2 inhibitors (EZH2Is) have been the subject of clinical trials, summarized in Table 1 [24,25,26,27,28,29]. 

In 2020, tazemetostat was the first agent in its class to receive approved by U.S. Food and Drug Administration (FDA) for the treatment of metastatic or locally advanced epithelioid sarcoma in adults and pediatric patients aged 16 years and older [25]. Tazemetostat is also being investigated as a chemotherapy agent for the treatment of several cancers, including follicular lymphoma, kidney cancer, diffuse large B-cell lymphoma, solid tumor, etc. [25]. These observations stimulated numerous EZH2 drug discovery programs and greatly encouraged us to develop more novel EZH2Is for the treatment of multiple cancer contexts. 

However, some agents’ relatively modest potency and pharmacokinetic properties remain drawbacks for future studies as optimal EZH2Is; for example, **GSK–126** is only administered by injection due to its poor pharmacokinetic properties [30]. Moreover, the single administration dosage of tazemetostat is up to 800 mg po bid [31]. Besides, the clinical therapeutic benefits of EZH2Is remain unsatisfactory and their applications are limited to only certain hematological malignancies. The abovementioned issues highlight the challenges that are associated with EZH2I therapy in the context of cancer. Therefore, the development of novel EZH2I structures could be a way to overcome these challenges. From the perspective of the structure of reported EZH2Is, while the initial structures have given invaluable insight into small molecule EZH2Is, a number of open questions remain. Most of the compounds take advantage of the structural characteristics of the pyridone moiety as an active pharmacophore that binds to EZH2 structures. This feature may serve as a boon for future inhibitor development. 

**Scheme 1:**
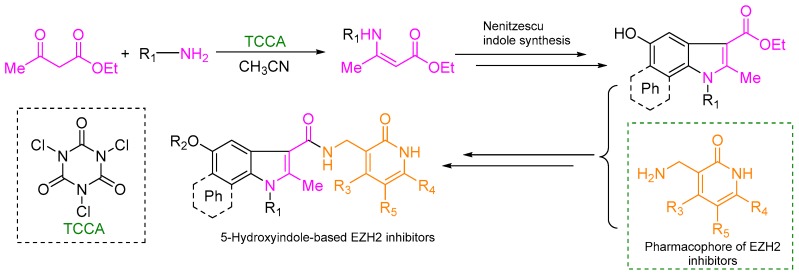
Synthetic route to target compounds via the key TCCA-catalyzed condensation and Nenitzescu synthesis.

Herein, based on the privileged 5-hydroxyindole scaffold and the main pyridone moiety pharmacophore, a novel series of 5-hydroxyindole-based EZH2Is has been designed and synthesized (Scheme 1), which then their antiproliferative effects against K562 cells in vitro and have been assessed and their ability to reduce cellular H3K27Me3 levels further characterized. Further a simple catalyst—1,3,5-trichloroisocyanuric acid (TCCA)—for promoting the condensation reactions of β-diketones or β-ketoesters and related amines during the preparation of compounds was accidentally discovered. Of note, TCCA has been previously reported as an inexpensive and relatively stable chlorination [32,33] and oxidation reagent [34,35] and our results therefore further expand the application scope of TCCA.

## 2. Results and Discussion 

### 2.1. Chemistry

#### 2.1.1. Condensation of Amines with β-Diketones or β-Ketoesters Catalyzed by TCCA

**Figure 2:**
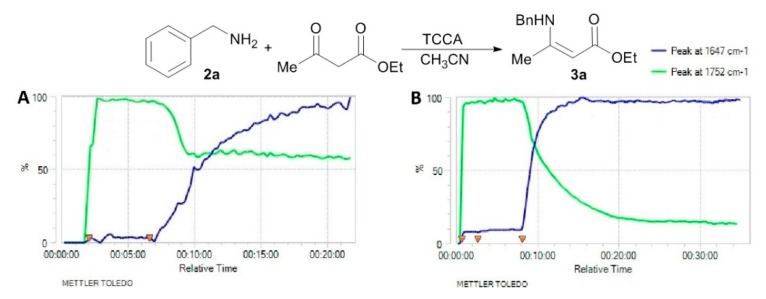
2D trends of the React IR experiment. (**A**) Ethyl acetoacetate (1.0 g, 7.68 mmol), benzylamine (0.82 g, 7.68 mmol) and MeCN (5 mL) were used; (**B**) Ethyl acetoacetate (1.0 g, 7.68 mmol), benzylamine (0.82 g, 7.68 mmol), TCCA (34.8 mg, 0.15 mmol) and MeCN (5 mL) were used. The reaction was conducted under ice bath conditions and allowed to naturally reach room temperature.

Generally, the condensation reactions of carbonyl compounds with amines are carried out via azeotropic removal of water due to their reversibility, which typically results in long reaction times, low yields and harsh reaction conditions, and these drawbacks limit their practical application. It was recently reported that some Lewis acids such as Zr(ClO_4_)_2_^.^6H_2_O, InBr_3_, Bi(O_2_CCF_3_)_3_, etc. could catalyze this transformation under mild conditions [36,37,38]. Herein, we systematically investigated the reaction conditions, including solvent, catalyst and molar ratio (seeing Supporting Information, Table S1). In this process, TCCA (at 2 mol%) could effectively accelerate the condensation reaction. The conversion of ethyl acetoacetate to the corresponding ethyl 3-(benzylamino)butyl-2-enoate was monitored by in situ React IR technology. As seen in Figure 2, less than 1 min after the addition of benzylamine to ethyl acetoacetate and TCCA in acetonitrile under ice bath conditions, a new sharp peak belonging to ethyl 3-(benzylamino)but-2-enoate appeared at 1647 cm^−1^, and the intensity of the peak increased gradually as the reaction proceeded. Of note also is the disappearance of the 1752 cm^−1^ band in parallel to the appearance of the 1647 cm^−1^ band. We believed that the new infrared absorption at 1647 cm^−1^ is attributed to the formation of a C-N bonds. The conversion of ethyl acetoacetate was less than 50% without any catalyst (Figure 2A), whereas ethyl acetoacetate was completely converted in 15 minutes when the reaction was catalyzed by TCCA (Figure 2B).

The reaction enjoys a wide substrate scope with respect to amine substrates, including primary aliphatic amines and aromatic amines (Table 2). This facile method was successfully used for β-diketones or β-ketoesters, and excellent yield and short reaction times were observed in all cases. The condensation reaction between aliphatic amines and β-ketoesters catalyzed by TCCA required short reaction times of only 10–20 min (entries 1–9). A more sterically encumbered phenyl group at the β-position of the β-ketoesters, and bulky amine substrate also reacted without incident (entries 8, 16). Since the aromatic amines have weaker nucleophilic activity, a long reaction time was also required (entries 3, 7, 8, 12–15). Moreover, electron-withdrawing groups on the benzene ring were not beneficial to the reaction (entry 15); for example, nitro groups, resulted in longer reaction times and lower conversion rates. Having established a facile method to the synthesis of intermediates, we next turned our efforts toward the synthesis of 5-hydroxyindole-based EZH2Is.

#### 2.1.2. Synthesis of 5-Hydroxyindole-Based EZH2Is

**Scheme 2:**
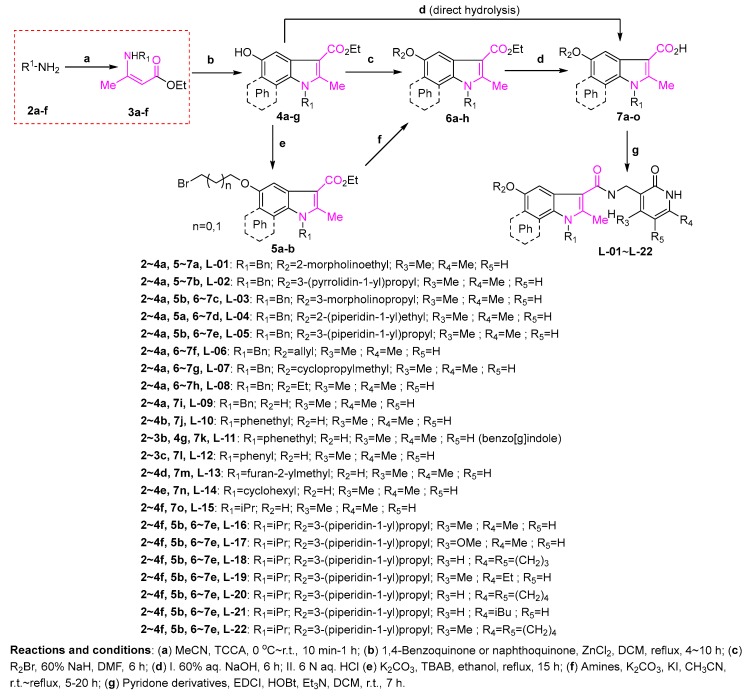
Synthetic route to 5-hydroxyindole-based EZH2Is.

With the established protocol in hand, a series of 5-hydroxyindoles were obtained, as depicted in Scheme 2. Initially, compounds **3a**–**h** were reacted with benzoquinone or naphthalene to yield 5-hydroxyindoles **4a**–**g** via an anhydrous ZnCl_2_-catalyzed Nenitzescu indole synthesis. The fragments **6a**–**e** were prepared from **4a** by alkylation and ammonolysis. Then, esters **6a**–**e** were hydrolyzed and acidified to afford carboxylic acids **7a**–**e**. Meanwhile, 5-hydroxyindole **4a** was transformed via alkylation with brominated alkanes in the presence of NaH into ethers **6f**–**h,** respectively, which were hydrolyzed and acidified to afford carboxylic acids **7f**–**h**. On the other hand, carboxylic acids **7i**–**o** were obtained from **4a**–**g** by direct hydrolysis and acidification. Finally, carboxylic acids **7a**–**o** were assembled with pyridone derivatives in the presence of EDCI and HOBt to furnish the target compounds **L–01**~**L–22**. The synthesis of the pyridone derivatives is described in the Supporting Information.

### 2.2. In Vitro Bioactivity Assay and SAR

Following the synthesis, the antiproliferative effects of the 5-hydroxyindole-based EZH2Is on K562 cells were then evaluated by a cell counting kit-8 (CCK-8) assay using tazemetostat as positive control. The results showed that most of the target compounds exhibited a similar antiproliferative effect compared with the control (Table 3). Different lengths of aliphatic chains were introduced to evaluate the appropriate distance between the indole scafford and the basic functional group. The results showed that the length of chain has no obvious effect on the inhibitory activity; for instance, compounds **L–01** (containing a two-carbon linker) and **L–03** (containing a three-carbon linker) showed comparable IC_50_ values. Additionally, it appeared that the terminal amine was essential for maintaining the activity. The hydroxy group at the C5 position of an indole substituted with an amine side chain was beneficial for the antiproliferative effect; for example, **L–01** and **L–06** had IC_50_ values of 83.8 μM and above 100 μM, respectively. Furthermore, a hydrophobic piperidine group (compounds **L–04** and **L–05**) had a beneficial effect on the IC_50_ value compared with that of hydrophilic morpholine moiety (compounds **L–01** and **L–03**). 

As a refinement, some 5-hydroxylindoles bearing different substituents at N1 were synthesized and investigated. Thus, all other *N*-substituted compounds (**L–10**, **L–12**~**L–15**), containing phenethyl (**L–10**), cyclohexyl (**L–14**) or (isopropyl, **L–15**) produced stronger inhibitory activities than benzyl (**L–09**) and phenyl (**L–12**). Particularly, it was noteworthy that the tricyclic compound **L–11** showed inhibition activity with an IC_50_ value of 55.2 μM. Among the different aliphatic alkanes (methyl, ethyl, propyl, isopropyl) on the pyridone ring, the results suggested a similar biochemical potency. Compounds **L–20** and **L–22** (with cyclohexane on the pyridone ring ) and **L–18** (with a cyclopentane on pyridone ring) showed less inhibition activities, while compounds **L–19** and **L–21** showed medium antiproliferative effect, with IC_50_ values of 68 μM and 52 μM, respectively.

**Figure 3:**
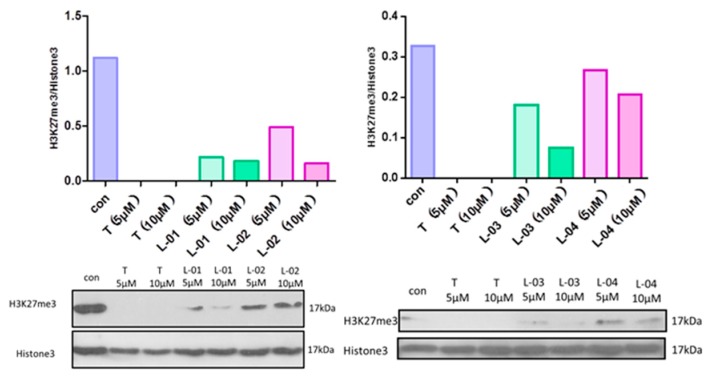
Effect of **L–01**~**L–04** on cellular H3K27Me3 levels as determined by western blot analysis. Western blot analysis of H3K27Me3 and Histone3 from whole cell extracts on K562 cells after treatment of 72 h. The letter T is abbreviation of tazemetostat.

To correlate the K562 cell inhibition results with EZH2, we assessed the cellular H3K27Me3 levels in K562 cells after being treated with compounds **L–01**~**L–04** and tazemetostat (Figure 3). Western blot data showed that **L–01**~**L–04** and tazemetostat significantly reduced cellular H3K27Me3 levels in K562 cells at concentrations of 5 μM and 10 μM in a dose-dependent manner. Thus, our results provide clear evidence that these compounds could inhibit the EZH2 protein at low molarity, which is consistent with the inhibition of K562 cell growth.

### 2.3. Molecular Docking

**Figure 4:**
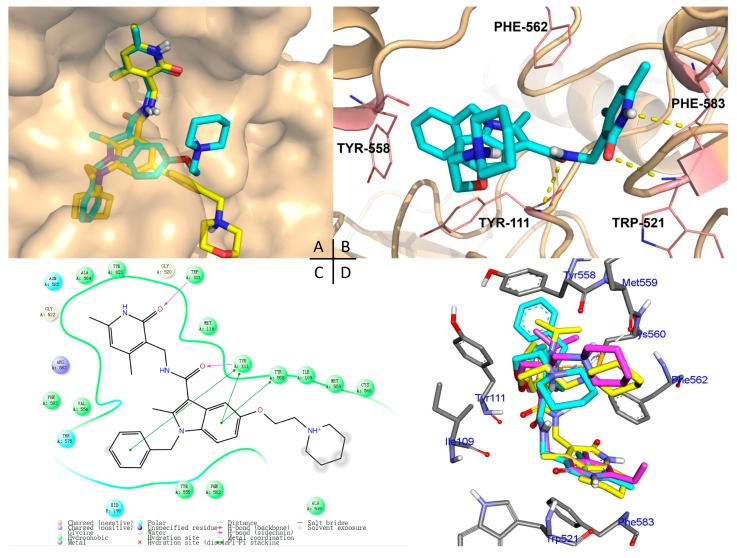
Molecular modeling of 5-hydroxyindole-based inhibitors bound to EZH2 (Note: PDB ID 4W2R). (**A**) Binding pocket surface of **L**–**04** (light blue) and tazemetostat (yellow); (**B**) Binding pose of **L**–**04**. Hydrogen bond contacts are shown with dashed lines and small molecules are shown as sticks; (**C**) Binding interaction of **L**–**04**; (**D**) Overlay of **L**–**04** (light blue), **L**–**19** (pink) and **L**–**22** (light yellow) docking poses.

We used molecular modeling to determine a binding mode of some representative inhibitors (**L–04**, **L–19**, **L–22** and tazemetostat) in the binding site of EZH2 (PDB ID: 4W2R). Initially, we docked **L–04** and tazemetostat to their respective binding conformations. As indicated in Figure 4A, **L–04** could maintain similar binding patterns compared with tazemetostat. The main pyridone moiety pharmacophore could form a hydrogen bond with Trp^521^. The carbonyl oxygen of the amide linker warhead engages EZH2 via tight hydrogen bonds with the catalytic tyrosine residue Tyr^111^. The indole scaffold and phenyl ring form face-to-face contacts with Tyr^111^ and Tyr^558^. The C5 position of the indole substituted with the amine side chain projects into the solvent region, where the protonated piperidine did not form additional hydrogen bonds (Figure 4B,C). 

Therefore, the substituents at the C5 position of indole could be modified to improve the pharmacokinetic properties; for example, compared with unsubstituted compound **L–09**, **L–04** bearing the basic chain showed lower binding energy. Small substituents such as C5 or C6-methyl or ethyl groups on the pyridone ring were well compatible, while big substituents such as 5 or 6-membered rings lost the hydrogen bond with Trp^521^, resulting in an invalid interaction mode with the EZH2 protein (Figure 4D). From the docking score perspective, **L–18** and **L–20** containing 5 or 6-membered ring substituents on the pyridone ring resulted in a higher binding energy. In all, our data suggests that **L–04** is an ideal template to develop improved EZH2Is using molecular modeling to rationally design new agents to treat multiple cancers.

## 3. Materials and Methods

### 3.1. General Information 

All reagents and starting materials were obtained from commercial sources and used as received. Melting points were measured with an X-4 melting point apparatus (Beijing Taike Instrument Co., Ltd., Beijing, China) and are uncorrected. ^1^H- and ^13^C-NMR spectra were recorded on an Ascent 600 MHz spectrometer (Bruker Billerica, MA, USA) using Me_4_Si (TMS) as the internal standard. Electrospray ionization mass spectra (ESI-MS) was recorded using an 1100 Series MSD Trap SL (Agilent, Santa Clara, CA, USA). High-resolution mass spectrometry (HRMS) results were recorded using an Agilent Technologies 6530 Accurate-Mass Q-TOF LC/MS. The reactions were monitored by thin-layer chromatography (TLC; HG/T2354-92, GF254), and terminated as judged by the consumption of starting material. The TLC plates were visualized with a UV lamp (Gongyi Yuhua Instrument Co., Ltd, Zhengzhou, China). React IR experiments were conducted using a ReactIR 15 (SN: 23267) equipped with an MCT DiComp (Diamond) probe detector (SN: 23146) connected via an AgX × 9.5 mm × 1.5 m fiber (silver halide) detector (Mettler Toledo, Zurich, Switzerland. The iC IR 4.3 Reaction Analysis Software was used during data collection and analysis.

### 3.2. Syntheses 

#### 3.2.1. General Procedure for the Syntheses of Ethyl Enoates **3a**–**p**

TCCA (714/34.8 mg, 31/0.15 mmol) was added to ethyl acetoacetate (20/1 g, 154/7.68 mmol) or *tert*-butyl 3-oxobutanoate (1.2 g, 7.59 mmol) or acetylacetone (0.77 g, 7.69 mmol) or ethyl benzoylacetate (1.48 g, 7.71 mmol) in acetonitrile (20 mL) under ice bath cooling, and the appropriate amine (0.23/0.012 mol) was added dropwise. The mixture was stirred under ice bath conditions and allowed to naturally warm up to room temperature for the indicated time. After the reaction was complete, the mixture was poured into water, extracted with dichloromethane (3 × 50 mL), washed with water (3 × 50 mL) and brine (50 mL). The organic solution was dried over magnesium sulfate, filtered, evaporated under reduced pressure and used in the following reaction without any further purification. 

*Ethyl 3-(benzylamino)but-2-enoate* (**3a**). Light yellow solid (32.0 g, 95%). M.p. 49–50 °C.^1^H-NMR (CDCl_3_) δ 8.95 (brs, 1H, NH), 7.34 (t, *J* = 7.6 Hz, 2H, ArH), 7.27*–*7.26 (m, 3H, ArH), 4.53 (s, 1H, C=C-H), 4.43 (d, *J* = 6.3 Hz, 2H, OCH_2_), 4.10 (q, *J* = 6.4 Hz, 2H, NCH_2_), 1.92 (s, 3H, CH_3_), 1.26 (t, *J* = 7.1 Hz, 3H, CH_3_). HRMS (ESI) calcd for C_13_H_17_NO_2_Na [M+Na]^+^: 242.1151, found: 242.1151.

*Ethyl 3-(phenethylamino)but-2-enoate* (**3b**). Colorless liquid (35.1 g, 98%). ^1^H-NMR (CDCl_3_) δ 8.65 (brs, 1H, NH), 7.30 (t, *J* = 7.3 Hz, 2H, ArH), 7.24*–*7.19 (m, 3H, ArH), 4.42 (s, 1H, C=C-H), 4.08 (q, *J* = 7.1 Hz, 2H, OCH_2_), 3.45*–*3.42 (m, 2H, NCH_2_), 2.85 (t, *J* = 7.6 Hz, 2H, PhCH_2_), 1.82 (s, 3H, CH_3_), 1.25 (t, *J* = 7.1 Hz, 3H, CH_3_). HRMS (ESI) calcd for C_14_H_19_NO_2_Na [M+Na]^+^: 256.1308, found: 256.1307.

*Ethyl 3-(phenylamino)but-2-enoate* (**3c**). Colorless liquid (29.0 g, 92%). ^1^H-NMR (DMSO-*d_6_*) δ 10.38 (s, 1H, NH), 7.39–7.34 (m, 2H, ArH), 7.19–7.17 (m, 3H, ArH), 4.69 (d, *J* = 0.4 Hz, 1H, C=C-H), 4.06 (q, *J* = 7.1 Hz, 2H, CH_2_), 2.01 (s, 3H, CH_3_), 1.20 (t, *J* = 7.1 Hz, 3H, CH_3_). HRMS (ESI) calcd for C_12_H_16_NO_2_ [M+H]^+^: 206.1176, found: 206.1170.

*Ethyl 3-((furan-2-ylmethyl)amino)but-2-enoate* (**3d**). Colorless liquid (30.2 g, 94%). ^1^H-NMR (CDCl_3_) δ 8.80 (brs, 1H, NH), 7.35 (d, *J* = 1.4 Hz, 1H, ArH), 6.31–6.30 (m, 1H, ArH), 6.19 (d, *J* = 3.2 Hz, 1H, ArH), 4.52 (s, 1H, C=C-H), 4.37 (d, *J* = 6.3 Hz, 2H, NCH_2_), 4.08 (q, *J* = 7.1 Hz, 2H, OCH_2_), 1.99 (s, 3H, CH_3_), 1.24 (t, *J* = 7.1 Hz, 3H, CH_3_). HRMS (ESI) calcd for C_11_H_15_NO_3_Na [M+Na]^+^: 232.0949, found: 232.0949.

*Ethyl 3-(cyclohexylamino)but-2-enoate* (**3e**). Colorless liquid (30.8 g, 95%). ^1^H-NMR (CDCl_3_) δ 8.63 (brs, 1H, NH), 4.39*–*4.37 (m, 1H, C=C-H), 4.10*–*4.05 (m, 2H, OCH_2_), 3.32*–*3.27 (m, 1H, NCH), 1.94*–*1.92 (m, 3H, CH_3_), 1.88*–*1.85 (m, 2H, CH_2_), 1.77*–*1.73 (m, 2H, CH_2_), 1.60–1.20 (m, 9H, CH_2_, CH_3_). HRMS (ESI) calcd for C_12_H_21_NO_2_Na [M+Na]^+^: 234.1465, found: 234.1461.

*Ethyl 3-(isopropylamino)but-2-enoate* (**3f**). Colorless liquid (25.3 g, 96%). ^1^H-NMR (CDCl_3_) δ 8.50 (brs, 1H, NH), 4.39 (s, 1H, C=C-H), 4.10–4.06 (m, 2H, OCH_2_), 3.70–3.66 (m, 1H, CH), 1.94 (s, 3H, CH_3_), 1.26–1.23 (m, 3H, CH_3_), 1.21-1.20 (m, 6H, 2´CH_3_). HRMS (ESI) calcd for C_9_H_18_NO_2_ [M+H]^+^: 172.1332, found: 172.1335.

*Ethyl 3-((4-ethoxyphenyl)amino)but-2-enoate* (**3g**). White solid (1.82 g, 95%). M.p. 53–54 °C. ^1^H-NMR (CDCl_3_) δ 10.14 (brs, 1H, NH), 7.01 (d, *J* = 8.8 Hz, 2H, ArH), 6.84 (d, *J* = 8.9 Hz, 2H, ArH), 4.64 (s, 1H, C=C-H), 4.16*–*4.12 (14.2 Hz, 7.1 Hz, 2 H, OCH_2_), 4.01 (q, *J* = 7.0 Hz, 2H, OCH_2_), 1.88 (s, 3H, CH_3_), 1.41 (t, *J* = 7.0 Hz, 3H, CH_3_), 1.28 (t, *J* = 7.1 Hz, 3H, CH_3_). HRMS (ESI) calcd for C_14_H_19_NO_3_Na [M+Na]^+^: 272.1257, found: 272.1252.

*Ethyl 3-((2,6-diisopropylphenyl)amino)but-2-enoate* (**3h**). White solid (2.0 g, 90%). M.p. 79–81 °C. ^1^H-NMR (CDCl_3_) δ 9.84 (brs, 1H, NH), 7.30*–*7.26 (m, 1H, ArH), 7.17 (d, *J* = 7.7 Hz, 2H, ArH), 4.68 (brs, 1 H, C=C-H), 4.17 (q, *J* = 7.1 Hz, 2 H, OCH_2_), 3.13*–*3.07 (m, 2H, 2´CH), 1.62 (s, 3H, CH_3_), 1.31 (t, *J* = 7.1 Hz, 3H, CH_3_), 1.22 (d, *J* = 6.9 Hz, 6H, 2´CH_3_), 1.12 (d, *J* = 6.8 Hz, 6H, 2´CH_3_). HRMS (ESI) calcd for C_18_H_27_NO_2_Na [M+Na]^+^: 312.1934, found: 312.1933.

*Ethyl 3-((1,3-dihydroxypropan-2-yl)amino)but-2-enoate* (**3i**). White solid (1.45 g, 94%). M.p. 74–75 ^o^C. ^1^H-NMR (CDCl_3_) δ 8.76 (d, *J* = 9.4 Hz, 1H, NH), 4.53 (s, 1H, C=C-H), 4.09 (q, *J* = 7.1 Hz, 2H, OCH_2_), 3.79*–*3.64 (m, 4H, 2´CH_2_), 3.69*–*3.65 (m, 1H, CH), 1.98 (s, 3H, CH_3_), 1.25 (t, *J* = 7.1 Hz, 3H, CH_3_). HRMS (ESI) calcd for C_9_H_17_NO_4_Na [M+Na]^+^: 226.1050, found: 226.1044.

*tert-Butyl 3-(benzylamino)but-2-enoate* (**3j**). White solid (1.96 g, 99%). M.p. 59~60 °C. ^1^H-NMR (CDCl_3_) δ 8.89 (brs, 1H, NH), 7.37*–*7.32 (m, 2H, ArH), 7.30*–*7.24 (m, 3H, ArH), 4.46 (s, 1H, C=C-H), 4.42 (d, *J* = 6.4 Hz, 2H, NCH_2_), 1.87 (s, 3H, CH_3_), 1.47 (s, 9H, 3´CH_3_). HRMS (ESI) calcd for C_15_H_21_NO_2_Na [M+Na]^+^: 270.1465, found: 270.1461.

*tert-Butyl 3-((2-hydroxyethyl)amino)but-2-enoate* (**3k**). White crystals (1.65 g, 94%). M.p. 51–53 °C. ^1^H-NMR (CDCl_3_) δ 8.59 (brs, 1H, NH), 4.43 (s, 1H, C=C-H), 3.74 (t, *J* = 5.3 Hz, 2H, CH_2_), 3.37 (q, *J* = 5.6 Hz, 2H, CH_2_), 1.92 (s, 3H, CH_3_), 1.46(s, 9H, 3´CH_3_). HRMS (ESI) calcd for C_10_H_19_NO_3_ Na [M+Na]^+^: 224.1257, found: 224.1252.

*tert-Butyl 3-((4-ethoxyphenyl)amino)but-2-enoate* (**3l**). White crystals (1.96 g, 93%). ^1^H-NMR (CDCl_3_) δ 10.10 (brs, 1H, NH), 7.01 (d, *J =* 8.8 Hz, 2H, ArH), 6.83 (d, *J =* 8.8 Hz, 2H, ArH), 4.58 (s, 1H, C=C-H), 4.01 (q, *J =* 7.0 Hz, 2H, OCH_2_), 1.86 (s, 3H, CH_3_), 1.50 (s, 9H, 3´CH_3_), 1.41 (t, *J =* 7.0 Hz, 3H, CH_3_). HRMS (ESI): calcd for C_16_H_23_NO_3_Na [M+Na]^+^: 300.1576; found: 300.1567. 

*tert-Butyl 3-(phenylamino)but-2-enoate* (**3m**). White crystals (1.63 g, 92%). ^1^H-NMR (CDCl_3_) δ 10.34 (brs, 1H, NH), 7.30 (t, *J =* 7.8 Hz, 2H, ArH), 7.13 (t, *J =* 7.4 Hz, 1H, ArH), 7.08 (d, *J =* 7.6 Hz, 2H, ArH), 4.62 (s, 1H, C=C-H), 1.50 (s, 9H, 3´CH_3_). HRMS (ESI): calcd for C_14_H_19_NO_2_Na [M+Na]^+^: 256.1313, found: 256.1307.

*4-(Phenylamino)pent-3-en-2-one* (**3n**). White crystals (1.21 g, 90%). M.p. 49–51 °C. ^1^H-NMR (CDCl_3_) δ 12.47 (brs, 1H, NH), 7.35–7.33 (m, 2H, ArH), 7.20 (t, *J* = 7.4 Hz, 1H, ArH), 7.11 (d, *J* = 7.5 Hz, 2H, ArH), 5.19 (brs, 1H, C=C-H), 2.10 (s, 3H, CH_3_), 2.00 (s, 3H, CH_3_). HRMS (ESI) calcd for C_11_H_13_NONa [M+Na]^+^: 198.0889, found: 198.0885. 

*4-((4-Nitrophenyl)amino)pent-3-en-2-one* (**3o**). Yellow solid (1.46 g, 86%). M.p. 143–144 °C. ^1^H-NMR (CDCl_3_) δ 12.78 (brs, 1H, NH), 8.21*–*8.20 (dd, *J* = 7.2 Hz, 2.0 Hz, 2H, ArH), 7.19*–*7.18 (dd, *J* = 7.2 Hz, 2.0 Hz, 2H, ArH), 5.33 (brs, 1H, C=C-H), 2.19 (s, 3H, CH_3_), 2.15 (s, 3H, CH_3_). HRMS (ESI) calcd for C_11_H_13_N_2_O_3_ [M+H]^+^: 221.0921, found: 221.0922. 

*Ethyl 3-(benzylamino)-3-phenylacrylate* (**3p**). White solid (1.99 g, 92%). M.p. 71–72 °C. ^1^H-NMR (CDCl_3_) δ 8.90 (brs, 1H, NH), 7.39*–*7.33 (m, 5H, ArH), 7.29 (t, *J* = 7.2 Hz, 2H, ArH), 7.23 (t, *J* = 7.3 Hz, 1H, ArH), 7.18 (d, *J* = 7.2 Hz, 2H, ArH), 4.67 (s, 1H, C=C-H), 4.26 (d, *J* = 6.5 Hz, 2H, NCH_2_), 4.15 (q, *J* = 7.1 Hz, 2H, OCH_2_), 1.28 (t, *J* = 7.1 Hz, 3H, CH_3_). HRMS (ESI) calcd for C_18_H_19_NO_2_Na [M+Na]^+^: 304.1308, found: 304.1307.

#### 3.2.2. General Procedure for the Syntheses of Ethyl 5-Hydroxyindolecarboxylates **4a**–**g**

Anhydrous ZnCl_2_ (1.26 g, 9.25 mmol) was added to a stirred suspension of *p*-benzoquinone (10 g, 92.5 mmol) or naphthoquinone (14.6 g, 92.5 mmol) in dry DCM (50 mL). After heating at reflux, a solution of crotonamine (92.5 mmol) in dry DCM (50 mL) was added into the mixture over 40 min and stirred at reflux for a further 45 min. The mixture was cooled to room temperature and held at 4 °C for 30 min to allow product precipitation. The solid was filtered and washed with DCM, water and acetonitrile to give a corresponding products **4a**–**g**. 

*Ethyl 1-benzyl-5-hydroxy-2-methyl-1H-indole-3-carboxylate* (**4a**). The title product was obtained from ethyl enoate **3a** and *p*-benzoquinone according to the general procedure described above as an off white solid (14.3 g, 50%). ^1^H-NMR (DMSO-*d_6_*) δ 8.96 (s, 1H, OH), 7.40 (d, *J* = 2.3 Hz, 1H, ArH), 7.33–7.23 (m, 4H, ArH), 7.01–7.00 (d, *J* = 7.3 Hz, 2H, ArH), 6.63 (dd, *J* = 8.7 Hz, 2.4 Hz, 1H, ArH), 5.44 (s, 2H, NCH_2_), 4.27 (q, *J* = 7.1 Hz, 2H, OCH_2_), 2.64 (s, 1H, CH_3_), 1.36 (t, *J* = 7.1 Hz, 3H, CH_3_). ^13^C-NMR (DMSO-*d_6_*) δ 165.6 (C=O_ester_), 153.3 (HOAr-C_indole_), 145.4 (ArC), 137.8 (ArC), 130.8 (ArC), 129.2 (ArC), 127.7 (ArC), 127.6 (ArC), 126.6 (ArC), 112.1 (ArC), 111.3 (C-C=O), 106.0 (ArC), 103.1 (ArC), 59.2 (OCH_2_), 46.3 (NCH_2_benzyl), 15.0 (CH_2_CH_3_), 12.3 (CH_3indole_). HRMS (ESI) calcd for C_19_H_19_NO_3_Na [M+Na]^+^: 332.1257, found: 332.1255.

*Ethyl 5-hydroxy-2-methyl-1-phenethyl-1H-indole-3-carboxylate* (**4b**). The title product was obtained from ethyl enoate **3b** and *p*-benzoquinone according to the general procedure described above as an off white solid (21.5 g, 72%). M.p. 175–177 °C. ^1^H-NMR (DMSO-*d_6_*) δ 8.97 (s, 1H, OH), 7.38 (d, *J =* 2.2 Hz, 1H, ArH), 7.33 (d, *J =* 8.7 Hz, 1H, ArH), 7.27–7.20 (m, 3H, ArH), 7.12 (d, *J =* 7.1 Hz, 2H, ArH), 6.68–6.66 (dd, *J =* 8.6 Hz, 2.3 Hz, 1H, ArH), 4.32 (t, *J =* 7.3 Hz, 2H, OCH_2_), 4.24 (q, *J =* 7.1 Hz, 2H, NCH_2_), 2.95 (t, *J =* 7.3 Hz, 2H, CH_2_), 2.4 (s, 3H, CH_3_), 1.34 (t, *J =* 7.1 Hz, 3H, CH_3_). ^13^C-NMR (DMSO-*d_6_*) δ 165.6 (C=O_ester_), 153.1 (HOAr-C_indole_), 145.5 (ArC), 138.7 (ArC), 130.1 (ArC), 129.4 (ArC), 128.8 (ArC), 127.7 (ArC), 127.0 (ArC), 111.8 (ArC), 111.0 (C-C=O), 106.0 (ArC), 102.4 (ArC), 59.1 (OCH_2_), 44.8 (NCH_2_), 35.6 (CH_2_Ph), 15.0 (CH_2_CH_3_), 11.7 (CH_3indole_). HRMS (ESI) calcd for C_20_H_21_NO_3_Na [M+Na]^+^: 346.1414, found: 346.1403.

*Ethyl 5-hydroxy-2-methyl-1-phenyl-1H-indole-3-carboxylate* (**4c**). The title product was obtained from ethyl enoate **3c** and *p*-benzoquinone according to the general procedure described above as a brown solid (16.1 g, 59%). M.p. 203–205 °C. ^1^H-NMR (DMSO-*d_6_*) δ 9.06 (s, 1H, OH), 7.63 (t, *J* = 7.4 Hz, 2H, ArH), 7.57 (t, *J* = 7.3 Hz, 1H, ArH), 7.47 (d, *J* = 2.3 Hz, 1H, ArH), 7.45 (d, *J* = 7.3 Hz, 2H, ArH), 6.78 (d, *J* = 8.7 Hz, 1H, ArH), 6.65–6.63 (dd, *J* = 8.8 Hz, 2.4 Hz, 1H, ArH), 4.32 (q, *J* = 7.1 Hz, 2H, OCH_2_), 2.49 (s, 3H, CH_3_), 1.38 (t, *J* = 7.1 Hz, 3H, CH_3_). ^13^C-NMR (DMSO-*d_6_*) δ 165.6 (C=O_ester_), 153.6 (HOAr-C_indole_), 145.3 (ArC), 136.5 (ArC), 131.9 (ArC), 130.3 (ArC), 129.3 (ArC), 128.4 (ArC), 127.5 (ArC), 112.5 (ArC), 111.2 (ArC), 105.9 (ArC), 104.0 (ArC), 59.4 (OCH_2_), 15.0 (CH_2_CH_3_), 13.3 (CH_3indole_). HRMS (ESI) calcd for C_18_H_17_NO_3_Na [M+Na]^+^: 318.1101, found: 318.1100.

*Ethyl 1-(furan-2-ylmethyl)-5-hydroxy-2-methyl-1H-indole-3-carboxylate* (**4d**). The title product was obtained from ethyl enoate **3d** and *p*-benzoquinone according to the general procedure described above as an off white solid (15.8 g, 57%). M.p. 207–211 °C. ^1^H-NMR (DMSO-*d_6_*) δ 8.95 (s, 1H, OH), 7.55 (d, *J* = 0.8 Hz, 1H, ArH), 7.44 (d, *J* = 8.7 Hz, 1H, ArH), 7.36 (d, *J* = 2.2 Hz, 1H, ArH), 6.68–6.66 (dd, *J* = 8.8 Hz, 2.3 Hz, 1H, ArH), 6.46 (d, *J* = 3.1 Hz, 1H, ArH), 6.39–3.38 (m, 1H, ArH), 5.39 (s, 2H, NCH_2_), 4.26 (q, *J* = 7.1 Hz, 2H, OCH_2_), 2.77 (s, 3H, CH_3_), 1.34 (t, *J* = 7.1 Hz, 3H, CH_3_). ^13^C-NMR (DMSO-*d_6_*) δ 165.5 (C=O_ester_), 153.2 (HOAr-C_indole_), 150.5 (ArC), 145.3 (ArC), 143.5 (ArC), 130.4 (ArC), 127.6 (ArC), 111.9 (ArC), 111.3 (ArC), 111.0 (ArC), 109.0 (ArC), 105.9 (ArC), 103.1 (ArC), 59.2 (OCH_2_), 15.0(CH_2_CH_3_), 12.2 (CH_3indole_). HRMS (ESI) calcd for C_17_H_17_NO_4_Na [M+Na]^+^: 322.1050, found: 322.1049.

*Ethyl 1-cyclohexyl-5-hydroxy-2-methyl-1H-indole-3-carboxylate* (**4e**). The title product was obtained from ethyl enoate **3e** and *p*-benzoquinone according to the general procedure described above as a pink solid (16.3 g, 67%). M.p. 245–247 °C. ^1^H-NMR (DMSO-*d_6_*) δ 8.90 (s, 1H, OH), 7.50 (d, *J* = 8.8 Hz, 1H, ArH), 7.40 (d, *J* = 2.3 Hz, 1H, ArH), 6.64–6.62 (dd, *J* = 8.8 Hz, 2.4 Hz, 1H, ArH), 4.28–4.24 (m, 3H, NCH, OCH_2_), 2.73 (s, 3H, CH_3_), 2.22–2.20 (m, 2H, CH_2_), 1.86–1.84 (m, 2H, CH_2_), 1.76–1.75 (m, 2H, CH_2_), 1.70–1.68 (m, 1H, CH_2_), 1.50–1.44 (m, 2H), 1.38–1.33 (m, 4H, CH_3_, CH_2_). ^13^C-NMR (DMSO-*d_6_*) δ 165.7 (C=O_ester_), 152.5 (HOAr-C_indole_), 145.0 (ArC), 129.0 (ArC), 128.6 (ArC), 113.5 (ArC), 111.4 (ArC), 106.1 (ArC), 102.5 (ArC), 59.1 (OCH_2_), 55.5 (NCH_2_), 30.8 (CH_2_), 26.1 (CH_2_), 25.2 (CH_2_), 15.0 (CH_2_CH_3_), 12.4 (CH_3indole_). HRMS (ESI) calcd for C_18_H_23_NO_3_Na [M+Na]^+^: 324.1570, found: 324.1567.

*Ethyl 5-hydroxy-1-isopropyl-2-methyl-1H-indole-3-carboxylate* (**4f**). The title product was obtained from ethyl enoate **3f** and *p*-benzoquinone according to the general procedure described above as a white solid (16.4 g, 68%). M.p. 168–170 °C. ^1^H-NMR (DMSO-*d_6_*) δ 8.86 (s, 1H, OH), 7.42 (d, *J* = 8.8 Hz, 1H, ArH), 7.38 (d, *J* = 2.2 Hz, 1H, ArH), 6.63*–*6.61 (dd, *J* = 8.8 Hz, 2.3 Hz, 1H, ArH), 4.77*–*4.73 (m, 1H, NCH), 2.69 (s, 3H, CH_3_), 1.57 (s, 6H, 2´CH_3_), 1.52 (d, *J* = 6.9 Hz, 3H, CH_3_). ^13^C-NMR (DMSO-*d_6_*) δ 165.3 (C=O_ester_), 152.5, 144.4 (ArC), 128.7 (ArC), 112.7 (ArC), 111.4 (ArC), 106.2 (ArC), 103.8 (ArC), 59.1 (OCH_2_), 47.0 (NCH_2_), 26.1 (CH_3_), 15.0 (CH_2_CH_3_), 12.5 (CH_3indole_). HRMS (ESI) calcd for C_17_H_23_NO_3_Na [M+Na]^+^: 312.1570, found: 312.1571.

*Ethyl 5-hydroxy-2-methyl-1-phenethyl-1H-benzo[g]indole-3-carboxylate* (**4g**). The title product was obtained from ethyl enoate **3b** and naphthoquinone according to the general procedure described above for the ethyl 5-hydroxyindolecarboxylate as a white solid (21.4 g, 62%). ^1^H-NMR (DMSO-*d_6_*) δ 9.77 (s, 1H, OH), 8.45 (d, *J* = 8.6 Hz, 1H, ArH_naphthalene_), 8.32 (dd, *J* = 8.3 Hz, 0.8 Hz, 1H, ArH_naphthalene_), 7.73 (s, 1H, ArH_naphthalene_), 7.67–7.64 (m, 1H, ArH_naphthalene_), 7.46 (t, *J* = 7.6 Hz, 1H, ArH_naphthalene_), 7.31 (t, *J* = 7.0 Hz, 2H, PhH), 7.27–7.25 (m, 1H, PhH), 7.15 (d, *J* = 7.0 Hz, 2H, PhH), 4.77 (t, *J* = 7.3 Hz, 2H, NCH_2_), 4.30 (q, *J* = 7.1 Hz, 2H, OCH_2_), 3.14 (t, *J* = 7.3 Hz, 2H, PhCH_2_), 2.49 (s, 3H, CH_3_), 1.39 (t, *J* = 7.1 Hz, 3H, CH_3_). ^13^C-NMR (DMSO-*d_6_*) δ 165.6 (C=O_ester_), 148.8 (HOAr-C), 143.6 (ArC), 138.3 (ArC), 129.3 (ArC), 129.1 (ArC), 127.2 (ArC), 126.9 (ArC), 125.0 (ArC), 124.0 (ArC), 123.6 (ArC), 123.1 (ArC), 123.0 (ArC), 122.7 (ArC), 120.7 (ArC), 104.2 (ArC), 102.3 (ArC), 59.4 (OCH_2_), 47.17 (NCH_2_), 35.7 (PhCH_2_), 15.0 (CH_3_), 11.9 (CH_3_). HRMS (ESI) calcd for C_24_H_23_NO_3_Na [M+Na]^+^: 396.1570, found: 396.1564. 

#### 3.2.3. General Procedure for the Syntheses of Compounds **5a**–**b**

*Ethyl 1-benzyl-5-(2-bromoethoxy)-2-methyl-1H-indole-3-carboxylate* (**5a**). 1,2-Dibromoethane (32 g, 0.17 mol) was added to a stirred mixture of **4a** (10 g, 32.3 mmol) and K_2_CO_3_ (28.2 g, 0.20 mol) in ethanol (200 mL), and the reaction was refluxed for 15 h. The mixture was cooled to room temperature, and filtered, washed with ethanol. The filtrate was evaporated under reduced pressure. The residue mixture was added water, after stirring 10 min, the suspension solid was filtered, washed with water, and dried to afford target product. The crude product was purified by column chromatography on silica gel (EA:PE = 1:50) as a white solid (10.0 g, 71%). ^1^H-NMR (CDCl_3_) δ 7.71 (d, *J* = 2.5 Hz, 1H, ArH), 7.29–7.08 (m, 4H, ArH), 6.96 (d, *J* = 6.9 Hz, 2H, ArH), 6.85 (dd, *J* = 8.8 Hz, 2.5 Hz, 1H, ArH), 5.33 (s, 2H, NCH_2_), 4.43–4.37 (m, 4H, 2 × OCH_2_), 3.67 (t, *J* = 6.4 Hz, 2H, Br-CH_2_), 2.71 (s, 3H, CH_3_), 1.46 (t, *J* = 7.1 Hz, 3H, CH_3_). ESI-MS m/z: 416.6 [M+H]^+^. 

*Ethyl 1-benzyl-5-(3-bromopropoxy)-2-methyl-1H-indole-3-carboxylate* (**5b**). The title compound was obtained as a white solid (12 g, 82%) by the same procedure described for compound **5a** but using 1,3-dibromopropane instead of 1,2-dibromoethane.

#### 3.2.4. General Procedure for Ammonolysis. Syntheses of **6a**–**e**

To a solution of **5a** (5 g, 12.0 mmol) or **5b** (5.16 g, 12.0 mmol) in acetonitrile (20 mL), KI (4.0 g, 24.0 mmol) were added, and the reaction mixture was heated to reflux for 30 min. After cooling to r.t., the amine (36 mmol) and anhydrous K_2_CO_3_ (6.6 g, 48.0 mmol) were added, and the mixture was heated to reflux for the indicated time while monitoring the disappearance of starting material by TLC. The cooled mixture was evaporated under reduced pressure, and water was added to the residue. After stirring for 10 min, the solid suspension was filtered, washed with water, and dried to afford target products **6a**–**e**. 

#### 3.2.5. General Procedure for the Syntheses of O-Substituted 5-Hydroxylindoles **6f**–**h**

5-Hydroxylindole **4a** (0.8 g, 2.59 mmol) was added to a stirred suspension of NaH (60% dispersion in mineral oil, 0.20 g, 517 mmol) in DMF (5 mL) under ice bath cooling. After 20 min, a haloalkane (3.88 mmol) was added dropwise, and the mixture was allowed to react for 6 h. The mixture was poured into water, and the resulting precipitate was filtered off, washed with water, and dried to afford the intermediates **6f**–**h**, which were used in the following reactions without any further purification. 

#### 3.2.6. General Hydrolysis Procedure: Syntheses of Compounds **7a**–**o**

Carboxylate compound **4a**–**g** or **6a**–**h** (2.0 mmol) were suspended in a mixture of EtOH (10 mL) and 60% NaOH aqueous (30 mmol), and refluxed for 3 h. The ethanol was removed under vacuum, and the mixture was cooled and acidified. The solid was filtered and dried to afford intermediates **7a**–**o**. 

#### 3.2.7. General Procedure for the Syntheses of the Target Compounds **L–01**~**L–22**

To a solution of indole derivatives (1 mmol), EDCI (0.29 g, 1.5 mmol), HOBt (0.20 g, 1.5 mmol) in dry dichloromethane (10 mL) Et_3_N (0.76 g, 5 mmol) was added and the mixture was stirred at room temperature for 30 min. Then, the corresponding pyridone derivatives (1 mmol) was added, and the resulting mixture was reacted at room temperature for indicated time while monitoring by TLC. When the reaction was complete the reaction mixture was poured into water, extracted with dichloromethane (15 × 3 mL), and washed with water (3 × 15 mL) and brine (15 mL). The organic solution was dried with anhydrous sodium sulfate, filtered and evaporated under reduced pressure. The solid residue was purified by column chromatography on silica gel. 

*1-Benzyl-N-((4,6-dimethyl-2-oxo-1,2-dihydropyridin-3-yl)methyl)-2-methyl-5-(2-morpholinoethoxy)-1H-indole-3-carboxamide* (**L–01**). The title product was obtained from **7a** and 3-(amino- methyl)-4,6-dimethylpyridin-2(1*H*)-one hydrochloride according to the general procedure described above as a white solid (0.29 g, 55%). M.p.158–160 °C. ^1^H-NMR (DMSO-d_6_) δ 11.45 (s, 1H, NHpyridone), 7.65 (t, *J* = 5.1 Hz, 1H, NHCH_2_), 7.32–7.21 (m, 5H, ArH), 6.98 (d, *J* = 7.4 Hz, 2H, ArH), 6.74 (dd, *J* = 8.8 Hz, 2.3 Hz, 1H, ArH), 5.89 (s, 1H, ArH_pyridone_), 5.41 (s, 2H, NCH_2benzyl_), 4.33 (d, *J* = 5.4 Hz, 2H, NHCH_2_), 4.10 (t, *J* = 5.6 Hz, 2H, OCH_2_), 3.58 (t, *J* = 4.5 Hz, 4H, 2 × OCH_2_), 2.70 (t, *J* = 5.6 Hz, 2H, NCH_2_), 2.50 (s, 3H, CH_3_), 2.49 (t, *J* = 10.0 Hz, 4H, 2 × NCH_2_), 2.26 (s, 3H, CH_3_), 2.12 (s, 3H, CH_3_). ^13^C-NMR (DMSO-d_6_) δ 165.1 (C=O_indole_), 163.9 (C=O_pyridone_), 154.1 (ArCH_pyridone_), 148.9 (ArCH_indole_), 142.1 (ArCH_pyridone_), 140.9 (N-C=C_indole_), 138.1 (ArCH_benzyl_), 131.4 (ArCH_benzyl_), 129.1 (ArCH_indole_), 127.7 (ArCH_benzyl_), 126.6 (ArCH_indole_), 126.5 (ArCH_pyridone_), 123.0 (ArCH_benzyl_), 111.8 (ArCH_indole_), 111.3 (N-C=C_indole_), 108.7 (ArCH_pyridone_), 108.1 (ArCH_indole_), 103.1 (ArCH_indole_), 66.7 (CH_2_OCH_2_), 66.4 (NCH_2_CH_2_O), 57.7 (NCH_2_CH_2_O), 54.2 (CH_2_NCH_2_), 46.2 (NCH_2_Ph), 35.6 (NHCH_2_), 19.4 (CH_3pyridone_), 18.7 (CH_3pyridone_), 11.9 (CH_3indole_). ESI-HRMS: m/z [M+Na]^+^ calcd for C_31_H_36_O_4_N_4_Na: 551.2629; found: 551.2621.

*1-Benzyl-N-((4,6-dimethyl-2-oxo-1,2-dihydropyridin-3-yl)methyl)-2-methyl-5-(3-(pyrrolidin-1-yl)propoxy)-1H-indole-3-carboxamide* (**L–02**). The title product was obtained from **7b** and 3-(aminomethyl)-4,6-dimethylpyridin-2(1*H*)-one hydrochloride according to the general procedure described above as a white solid (0.23 g, 34%). M.p.195-196 °C. ^1^H-NMR (DMSO-d_6_) δ 11.57 (brs, 1H, NH_pyridone_), 7.65 (t, *J* = 5.2 Hz, 1H, NHCH_2_), 7.32–7.18 (m, 5H, ArH), 6.98 (d, *J* = 7.4 Hz, 2H, ArH), 6.73–6.71 (dd, *J* = 8.8 Hz, 2.2 Hz, 1H, ArH), 5.90 (s, 1H, ArH_pyridone_), 5.41 (s, 2H, NCH_2benzyl_), 4.33 (d, *J* = 5.3 Hz, 2H, NHCH_2_), 4.03 (t, *J* = 6.2 Hz, 2H, OCH_2_), 2.71 (brs, 2H, CH_2_), 2.63 (brs, 4H, 2 × CH_2_), 2.53 (s, 3H, CH_3_), 2.27 (s, 3H, CH_3_), 2.12 (s, 3H, CH_3_), 1.96–1.94 (m, 2H, CH_2_), 1.73 (brs, 4H, 2 × CH_2_). ^13^C-NMR (DMSO-d_6_) δ 165.1 (C=O_indole_), 163.9 (C=O_pyridone_), 154.2 (ArCH_pyridone_), 148.8 (ArCH_indole_), 143.1 (ArCH_pyridone_), 140.8 (N-C=C_indole_), 138.1 (ArCH_benzyl_), 131.4 (ArCH_benzyl_), 129.1 (ArCH_indole_), 127.7 (ArCH_benzyl_), 126.6 (ArCH_indole_), 126.3 (ArCH_pyridone_), 123.0 (ArCH_benzyl_), 111.9 (ArCH_indole_), 111.3 (N-C=C_indole_), 108.7 (ArCH_pyridone_), 108.1 (ArCH_indole_), 103.0 (ArCH_indole_), 66.4 (OCH_2_), 53.9 (NCH_2_CH_2_), 52.6 (CH_2_NCH_2tetrahydropyrrole_), 46.2 (NCH_2_Ph), 35.6 (NHCH_2_), 28.1 (CH_2_CH_2_CH_2_), 23.5 (CH_2_CH_2tetrahydropyrrole_), 19.4 (CH_3pyridone_), 18.7 (CH_3pyridone_), 11.9 (CH_3indole_). ESI-HRMS: m/z [M+H]^+^ calcd for C_32_H_39_O_3_N_4_: 527.3017; found: 527.3046.

*1-Benzyl-N-((4,6-dimethyl-2-oxo-1,2-dihydropyridin-3-yl)methyl)-2-methyl-5-(3-morpholinopropoxy)-1H-indole-3-carboxamide* (**L–03**). The title product was obtained from **7c** and 3-(aminomethyl)-4,6-dimethylpyridin-2(1*H*)-one hydrochloride according to the general procedure described above as a yellow solid (0.30 g, 86%). M.p. 158-160 °C. ^1^H-NMR (DMSO-d_6_) δ 11.53 (s, 1H, NH_pyridone_), 7.65 (t, *J* = 5.1 Hz, 1H, NHCH_2_), 7.31–7.21 (m, 5H, ArH), 6.98 (d, *J* = 7.5 Hz, 2H, ArH), 6.72 (dd, *J* = 8.8 Hz, 2.3 Hz, 1H, ArH), 5.89 (s, 1H, ArH_pyridone_), 5.41 (s, 2H, NCH_2benzyl_), 4.33 (d, *J* = 5.3 Hz, 2H, NHCH_2_), 4.10 (t, *J* = 6.3 Hz, 2H, OCH_2_), 3.57 (t, *J* = 4.4 Hz, 4H, 2 × OCH_2_), 2.50 (s, 3H, CH_3_), 2.40 (t, *J* = 7.2 Hz, 2H, CH_2_), 2.37 (s, 4H, 2 × NCH_2_), 2.26 (s, 3H, CH_3_), 2.12 (s, 3H, CH_3_), 1.91–1.86 (m, 2H, CH_2_). ^13^C-NMR (DMSO-d_6_) δ 165.1 (C=O_indole_), 163.9 (C=O_pyridone_), 154.3 (ArCH_pyridone_), 148.9 (ArCH_indole_), 143.0 (ArCH_pyridone_), 140.8 (N-C=C_indole_), 138.2 (ArCH_benzyl_), 131.3 (ArCH_benzyl_), 129.1 (ArCH_benzyl_), 127.6 (ArCH_indole_), 126.6 (ArCH_indole_), 126.3 (ArCH_pyridone_), 123.0 (ArCH_benzyl_), 111.8 (ArCH_indole_), 111.3 (N-C=C_indole_), 108.7 (ArCH_pyridone_), 108.1 (ArCH_indole_), 103.0 (ArCH_indole_), 66.8 (CH_2_CH_2_O), 66.6 (CH_2_OCH_2_), 55.5 (CH_2_NCH_2_), 53.9 (NCH_2_CH_2_CH_2_O), 46.2 (NCH_2_Ph), 35.6 (NHCH_2_), 26.6 (NCH_2_CH_2_CH_2_O), 19.4 (CH_3pyridone_), 18.7 (CH_3pyridone_), 12.0 (CH_3indole_). ESI-HRMS: m/z [M+Na]^+^ calcd for C_32_H_38_O_4_N_4_Na: 565.2785; found: 565.2797.

*1-Benzyl-N-((4,6-dimethyl-2-oxo-1,2-dihydropyridin-3-yl)methyl)-2-methyl-5-(2-(piperidin-1-yl)ethoxy-1H-indole-3-carboxamide* (**L–04**). The title product was obtained from **7d** and 3-(aminomethyl)-4,6-dimethylpyridin-2(1*H*)-one hydrochloride according to the general procedure described above as a white solid (0.26 g, 50%). M.p.215–217 °C. ^1^H-NMR (DMSO-d_6_) δ 11.54 (s, 1H, NH_pyridone_), 7.65 (t, *J* = 4.9 Hz, 1H, NHCH_2_), 7.32–7.21 (m, 5H, ArH), 6.98 (d, *J* = 7.5 Hz, 2H, ArH), 6.73 (m, 1H, ArH), 5.89 (s, 1H, ArH_pyridone_), 5.41 (s, 2H, NCH_2benzyl_), 4.33 (d, *J* = 5.1Hz, 2H, NHCH_2_), 4.06 (t, *J* = 5.6 Hz, 2H, OCH_2_), 2.66 (t, *J* = 5.6 Hz, 2H, NCH_2_), 2.53 (s, 3H, CH_3_), 2.43 (s, 4H, 2 × CH_2_), 2.26 (s, 3H, CH_3_), 2.12 (s, 3H, CH_3_), 1.49 (t, *J* = 5.3 Hz, 4H, 2 × CH_2_), 1.37 (m, 2H, CH_2_). ^13^C-NMR (DMSO-d_6_) δ 165.1 (C=O_indole_), 163.9 (C=O_pyridone_), 154.2 (ArCH_pyridone_), 148.7 (ArCH_indole_), 143.1 (ArCH_pyridone_), 140.9 (N-C=C_indole_), 138.1 (ArCH_benzyl_), 131.3 (ArCH_benzyl_), 129.1 (ArCH_benzyl_), 127.6 (ArCH_indole_), 126.6 (ArCH_indole_), 126.3 (ArCH_pyridone_), 123.0 (ArCH_benzyl_), 111.7 (ArCH_indole_), 111.3 (N-C=C_indole_), 108.7 (ArCH_pyridone_), 108.1 (ArCH_indole_), 103.1 (ArCH_indole_), 66.6 (NCH_2_CH_2_O), 58.1 (CH_2_NCH_2_), 54.9 (NCH_2_Ph), 46.2 (NCH_2_CH_2_O), 35.6 (NHCH_2_), 26.1 (CH_2_CH_2_CH_2piperidine_), 24.5 (CH_2_CH_2_CH_2piperidine_), 19.4 (CH_3pyridone_), 18.7 (CH_3pyridone_), 11.9 (CH_3indole_). ESI-HRMS: m/z [M+H]^+^ calcd for C_32_H_38_O_4_N_4_Na: 527.3017; found: 527.3018.

*1-Benzyl-N-((4,6-dimethyl-2-oxo-1,2-dihydropyridin-3-yl)methyl)-2-methyl-5-(3-(piperidin-1-yl)propoxy)-1H-indole-3-carboxamide* (**L–05**). The title product was obtained from **7e** and 3-(aminomethyl)-4,6-dimethylpyridin-2(1*H*)-one hydrochloride according to the general procedure described above as a white solid (0.20 g, 41%). M.p. 210–212 °C. ^1^H-NMR (DMSO-d_6_) δ 11.53 (s, 1H, NH_pyridone_), 7.68 (s, 1H, NHCH_2_), 7.31–7.21 (m, 5H, ArH), 6.98 (d, *J* = 7.5 Hz, 2H, ArH), 6.72 (dd, *J* = 8.8 Hz, 1.9 Hz, 1H, ArH), 5.89 (s, 1H, ArH_pyridone_), 5.41 (s, 2H, NCH_2benzyl_), 4.33 (d, *J* = 5.2 Hz, 2H, NHCH_2_), 3.99 (t, *J* = 6.2 Hz, 2H, OCH_2_), 2.53 (s, 3H, CH_3_), 2.38 (t, *J* = 7.1 Hz, 2H, NCH_2_), 2.33 (s, 4H, 2 × NCH_2_), 2.26 (s, 3H, CH_3_), 2.12 (s, 3H, CH_3_), 1.89–1.84 (m, 2H, CH_2_), 1.49 (t, *J* = 5.3 Hz, 4H, 2 × CH_2_), 1.37 (s, 2H, CH_2_). ^13^C-NMR (DMSO-d_6_) δ 165.1 (C=O_indole_), 163.9 (C=O_pyridone_), 154.3 (ArCH_pyridone_), 148.8 (ArCH_indole_), 143.1 (ArCH_pyridone_), 140.8 (N-C=C_indole_), 138.1 (ArCH_benzyl_), 131.3 (ArCH_benzyl_), 129.1 (ArCH_benzyl_), 127.6 (ArCH_indole_), 126.6 (ArCH_indole_), 126.3 (ArCH_pyridone_), 123.0 (ArCH_benzyl_), 111.8 (ArCH_indole_), 111.3 (N-C=C_indole_), 108.7 (ArCH_pyridone_), 108.1 (ArCH_indole_), 103.0 (ArCH_indole_), 66.8 (CH_2_O), 55.8 (NCH_2_CH_2_CH_2_O), 54.6 (CH_2_NCH_2_), 46.2 (NCH_2_Ph), 35.6 (NHCH_2_), 27.0 (NCH_2_CH_2_CH_2_O), 26.1 (CH_2_CH_2_CH_2piperidine_), 24.6 (CH_2_CH_2_CH_2piperidine_), 19.4 (CH_3pyridone_), 18.7 (CH_3pyridone_), 11.9 (CH_3indole_). ESI-HRMS: m/z [M+H]^+^ calcd for C_33_H_41_O_3_N_4_: 541.3173; found: 541.3179.

*5-(Allyloxy)-1-benzyl-N-((4,6-dimethyl-2-oxo-1,2-dihydropyridin-3-yl)methyl)-2-methyl-1H-indole-3-carboxamide* (**L–06**). The title product was obtained from **7f** and 3-(amino- methyl)-4,6-dimethylpyridin-2(1*H*)-one hydrochloride according to the general procedure described above as a white solid (0.35 g, 78%). M.p. 245–47 °C. ^1^H-NMR (DMSO-*d_6_*) δ 11.58 (brs, 1H, NH_pyridone_), 7.69 (m, 1H, NHCH_2_), 7.33–7.21 (m, 5H, ArH), 6.98 (d, *J* = 7.4 Hz, 2H, ArH), 6.77–6.75 (dd, *J* = 8.9 Hz, 2.3 Hz, 1H, ArH), 6.10–6.04 (m, 1H, ArH), 5.89 (s, 1H, ArH_pyridone_), 5.41 (s, 2H, NCH_2benzyl_), 5.39 (d, *J* = 1.6 Hz, 1H, ArH), 5.24–5.23 (m, ArH), 4.58 (d, *J* = 5.1 Hz, 2H, OCH_2_), 4.33 (d, *J* = 5.3 Hz, 2H, NHCH_2_), 2.54 (s, 3H, CH_3_), 2.26 (s, 3H, CH_3_), 2.12 (s, 3H, CH_3_). ^13^C-NMR (DMSO-*d_6_*) δ 165.0 (C=O_indole_), 163.9 (C=O_pyridone_), 153.9 (ArCH_pyridone_), 148.8 (ArCH_indole_), 143.1 (ArCH_pyridone_), 141.1 (N-C=C_indole_), 138.1 (ArCH_benzyl_), 134.8 (CH_2_=CH), 131.4 (ArCH_benzyl_), 129.1 (ArCH_benzyl_), 127.7 (ArCH_indole_), 126.6 (ArCH_indole_), 126.2 (ArCH_pyridone_), 122.9 (ArCH_benzyl_), 117.5 (CH_2_=CH), 111.8 (ArCH_indole_), 111.3 (N-C=C_indole_), 108.6 (ArCH_pyridone_), 108.1 (ArCH_indole_), 103.4 (ArCH_indole_), 69.1 (CH_2_O), 46.2 (NCH_2_Ph), 35.6 (NHCH_2_), 19.4 (CH_3pyridone_), 18.7 (CH_3pyridone_), 11.9 (CH_3indole_). ESI-HRMS: m/z [M+Na]^+^ calcd for C_28_H_29_O_3_N_3_Na: 478.2101; found: 478.2097.

*1-Benzyl-5-(cyclopropylmethoxy)-N-((4,6-dimethyl-2-oxo-1,2-dihydropyridin-3-yl)methyl)-2-methyl-1H-indole-3-carboxamide* (**L–07**). The titled product was obtained from **7g** and 3-(aminomethyl)-4,6-dimethylpyridin-2(1*H*)-one hydrochloride according to the general procedure described above as a white solid (0.30 g, 64%). M.p.136–138 °C. ^1^H-NMR (DMSO-d_6_) δ 11.53 (s, 1H, NH_pyridone_), 7.63 (t, *J* = 5.2 Hz, 1H, NHCH_2_), 7.32–7.21 (m, 5H, ArH), 6.98 (d, *J* = 7.4 Hz, 2H, ArH), 6.74–6.73 (dd, *J* = 8.8 Hz, 2.3 Hz, 1H, ArH), 5.89 (s, 1H, ArH_pyridone_), 5.41 (s, 2H, NCH_2benzyl_), 4.33 (d, *J* = 8.6 Hz, 2H, NHCH_2_), 3.81 (d, *J* = 6.9 Hz, 2H, OCH_2_), 2.53 (s, 3H, CH_3_), 2.26 (s, 3H, CH_3_), 2.12 (s, 3H, CH_3_), 1.24–1.19 (m, 1H, CH), 0.58–0.55 (m, 2H, CH_2_), 0.33 (q, *J* = 5.2 Hz, 2H, CH_2_). ^13^C-NMR (DMSO-d_6_) δ 165.1 (C=O_indole_), 163.9 (C=O_pyridone_), 154.3 (ArCH_pyridone_), 148.9 (ArCH_indole_), 143.1 (ArCH_pyridone_), 140.9 (N-C=C_indole_), 138.1 (ArCH_benzyl_), 131.3 (ArCH_benzyl_), 129.1 (ArCH_benzyl_), 127.6 (ArCH_indole_), 126.6 (ArCH_indole_), 126.3 (ArCH_pyridone_), 122.9 (ArCH_benzyl_), 111.8 (ArCH_indole_), 111.3 (N-C=C_indole_), 108.6 (ArCH_pyridone_), 108.1 (ArCH_indole_), 103.8 (ArCH_indole_), 72.9 (CH_2_O), 46.2 (NCH_2_Ph), 35.6 (NHCH_2_), 19.4 (CH_3pyridone_), 18.7 (CH_3pyridone_), 11.9 (CH_3indole_), 10.9 (CH), 3.6 (CH_2_CH_2_). ESI-HRMS: m/z [M+Na]^+^ calcd for C_29_H_31_O_3_N_3_Na: 492.2258; found: 492.2266.

*1-Benzyl-N-((4,6-dimethyl-2-oxo-1,2-dihydropyridin-3-yl)methyl)-5-ethoxy-2-methyl-1H-indole-3-carboxamide* (**L–08**). The title product was obtained from **7h** and 3-(amino- methyl)-4,6-dimethylpyridin-2(1*H*)-one hydrochloride according to the general procedure described above as a pink solid (0.30 g, 43%). M.p. 263–264 °C. ^1^H-NMR (DMSO-*d_6_*) δ 11.54 (s, 1H, NH_pyridone_), 7.65 (s, 1H, NHCH_2_), 7.32–7.21 (m, 5H, ArH), 6.98 (d, *J* = 7.6 Hz, 2H, ArH), 6.73 (d, *J* = 8.8 Hz, 1H, ArH), 5.89 (s, 1H, ArH_pyridone_), 5.41 (s, 2H, NCH_2benzyl_), 4.33 (d, *J* = 5.3 Hz, 2H, NHCH_2_), 4.03 (q, *J* = 6.8 Hz, 2H, CH_2_), 2.54 (s, 3H, CH_3_), 2.26 (s, 3H, CH_3_), 2.12 (s, 3H, CH_3_), 1.34 (t, *J* = 6.9 Hz, 3H, CH_3_). ^13^C-NMR (DMSO-*d_6_*) δ 165.1 (C=O_indole_), 164.0 (C=O_pyridone_), 154.2 (ArCH_pyridone_), 148.7 (ArCH_indole_), 143.2 (ArCH_pyridone_), 141.0 (N-C=C_indole_), 138.1 (ArCH_benzyl_), 131.3 (ArCH_benzyl_), 129.1 (ArCH_benzyl_), 127.6 (ArCH_indole_), 126.6 (ArCH_indole_), 126.2 (ArCH_pyridone_), 122.9 (ArCH_benzyl_), 111.8 (ArCH_indole_), 111.3 (N-C=C_indole_), 108.6 (ArCH_pyridone_), 108.1 (ArCH_indole_), 102.8 (ArCH_indole_), 63.8 (CH_2_O), 46.2 (NCH_2_Ph), 35.7 (NHCH_2_), 19.4 (CH_3pyridone_), 18.7 (CH_3pyridone_), 15.4 (CH_2_CH_3_), 11.9 (CH_3indole_). ESI-HRMS: m/z [M+Na]^+^ calcd for C_27_H_29_O_3_N_3_Na: 466.2101; found: 466.2107.

*1-Benzyl-N-((4,6-dimethyl-2-oxo-1,2-dihydropyridin-3-yl)methyl)-5-hydroxy-2-methyl-1H-indole-3-carboxamide* (**L–09**). The title product was obtained from **7i** and 3-(aminomethyl)-4,6-dimethylpyridin-2(1*H*)-one hydrochloride according to the general procedure described above as a white solid (0.40, 61%). M.p. 288–290 °C. ^1^H-NMR (DMSO-*d_6_*) δ 11.55 (s, 1H, NH_pyridone_), 8.85 (s, 1H, OH), 7.44 (t, *J* = 5.3 Hz, 1H, NHCH_2_), 7.29–7.20 (m, 4H, ArH), 7.13 (d, *J* = 2.0 Hz, 1H, ArH), 6.99 (d, *J* = 7.6 Hz, 2H, ArH), 6.61–6.59 (dd, *J* = 8.6 Hz, 2.0 Hz, 1H, ArH), 5.89 (s, 1H, ArH_pyridone_), 5.37 (s, 2H, NCH_2benzyl_), 4.33 (d, *J* = 5.4 Hz, 2H, NHCH_2_), 2.52 (s, 3H, CH_3_), 2.26 (s, 3H, CH_3_), 2.12 (s, 3H, CH_3_). ^13^C-NMR (DMSO-*d_6_*) δ 165.4 (C=O_indole_), 163.9 (C=O_pyridone_), 152.6 (ArCH_pyridone_), 148.9 (ArCH_indole_), 143.1 (ArCH_pyridone_), 140.7 (N-C=C_indole_), 138.2 (ArCH_benzyl_), 130.7 (ArCH_benzyl_), 129.1 (ArCH_benzyl_), 127.6 (ArCH_indole_), 126.6 (ArCH_indole_), 122.9 (ArCH_benzyl_), 111.6 (ArCH_indole_), 111.0 (N-C=C_indole_), 108.1 (ArCH_pyridone_), 108.0 (ArCH_indole_), 104.4 (ArCH_indole_), 46.1 (NCH_2_Ph), 35.6 (NHCH_2_), 19.4 (CH_3pyridone_), 18.7 (CH_3pyridone_), 12.0 (CH_3indole_). ESI-HRMS: m/z [M+Na]^+^ calcd for C_25_H_25_O_3_N_3_Na: 438.1788; found: 438.1798.

*N-((4,6-Dimethyl-2-oxo-1,2-dihydropyridin-3-yl)methyl)-5-hydroxy-2-methyl-1-phenethyl-1H-indole-3-carboxamide* (**L–10**)**.** The title product was obtained from **7j** and 3-(amino- methyl)-4,6-dimethylpyridin-2(1*H*)-one hydrochloride according to the general procedure described above as a white solid (0.25 g, 58%). M.p.242–245 °C. ^1^H-NMR (DMSO-*d_6_*) δ 11.54 (brs, 1H, NH_pyridone_), 8.84 (brs, 1H, OH), 7.32 (t, *J* = 5.0 Hz, 1H, NHCH_2_), 7.28–7.19 (m, 4H, ArH), 7.16 (d, *J* = 7.1 Hz, 2H, ArH), 7.10 (d, *J* = 1.7 Hz, 1H, ArH), 6.65–6.63 (dd, *J* = 8.6 Hz, 1.9 Hz, 1H, ArH), 5.89 (s, 1H, ArH_pyridone_), 4.30 (d, *J* = 5.3 Hz, 2H, NHCH_2_), 4.26 (t, *J* = 7.3 Hz, 2H, NCH_2_), 2.91 (t, *J* = 7.3 Hz, 2H, PhCH_2_), 2.37 (s, 3H, CH_3_), 2.25 (s, 3H, CH_3_), 2.12 (s, 3H, CH_3_). ^13^C-NMR (DMSO-*d_6_*) δ 165.4 (C=O_indole_), 163.9 (C=O_pyridone_), 152.4 (ArCH_pyridone_), 148.8 (ArCH_indole_), 143.1 (ArCH_pyridone_), 140.8 (N-C=C_indole_), 138.9 (PhCH), 130.1 (PhCH), 129.4 (PhCH), 128.8 (ArCH_indole_), 126.9 (ArCH_indole_), 126.5 (ArCH_pyridone_), 123.0 (PhCH), 111.4 (ArCH_indole_), 110.8 (N-C=C_indole_), 108.1 (ArCH_pyridone_), 107.4 (ArCH_indole_), 104.3 (ArCH_indole_), 44.6 (NCH_2_CH_2_Ph), 35.8 (NCH_2_CH_2_Ph), 35.6 (NHCH_2_), 19.4 (CH_3pyridone_), 18.6 (CH_3pyridone_), 11.6 (CH_3indole_). ESI-HRMS: m/z [M+Na]^+^ calcd for C_26_H_27_O_3_N_3_Na: 452.1945; found: 452.1980.

*N-((4,6-Dimethyl-2-oxo-1,2-dihydropyridin-3-yl)methyl)-5-hydroxy-2-methyl-1-phenethyl-1H-benzo- [g]indole-3-carboxamide* (**L–11**). The title product was obtained from **7k** and 3-(amino- methyl)-4,6-dimethylpyridin-2(1*H*)-one hydrochloride according to the general procedure described above as a white solid (0.35 g, 64%). M.p. 240 °C (decomposes). ^1^H-NMR (DMSO-*d_6_*) δ 11.36 (brs, 1H, NH_pyridone_), 8.42 (d, *J*=8.6 Hz, 1H, ArH), 8.29 (d, *J*=8.3 Hz, 1H, ArH), 7.62 (t, *J*=7.5 Hz, 1H, NHCH_2_), 7.44-7.41 (m, 2H, ArH), 7.37 (s, 1H, ArH), 7.34-7.29 (m, 2H, ArH), 7.27-7.25 (m, 1H, ArH), 7.21 (d, *J*=7.4 Hz, 2H, ArH), 5.89 (s, 1H, ArH_pyridone_), 4.70 (t, *J*=7.4 Hz, 2H, NCH_2_), 4.34 (d, *J*=5.0 Hz, 2H, NHCH_2_), 3.08 (t, *J*=7.3 Hz, 2H, PhCH_2_), 2.37 (s, 3H, CH_3_), 2.27 (s, 3H, CH_3_), 2.12 (s, 3H, CH_3_). ^13^C-NMR (DMSO-*d_6_*) δ 165.6 (C=O_indole_), 163.9 (C=O_pyridone_), 149.2 (ArCH_pyridone_), 148.0 (ArCH_indole_), 143.1 (ArCH_pyridone_), 138.5 (N-C=C_indole_), 137.6 (PhCH), 129.3 (PhCH), 129.1 (ArCH_indole_), 127.2 (ArCH_indole_), 126.7 (ArCH_pyridone_), 124.0 (ArCH), 124.0 (ArCH), 123.5 (ArCH), 122.9 (ArCH), 122.8 (ArCH), 122.7 (ArCH), 122.6 (ArCH), 120.6 (ArCH), 110.3 (N-C=C_indole_), 108.1 (ArCH_pyridone_), 101.2 (ArCH_indole_), 47.1 (NCH_2_CH_2_Ph), 36.0 (NCH_2_CH_2_Ph), 35.6 (NHCH_2_), 19.4 (CH_3pyridone_), 18.7 (CH_3pyridone_), 11.7 (CH_3indole_). ESI-HRMS: m/z [M+Na]^+^ calcd for C_30_H_29_O_3_N_3_Na: 502.2101; found: 502.2124. 

*N-((4,6-Dimethyl-2-oxo-1,2-dihydropyridin-3-yl)methyl)-5-hydroxy-2-methyl-1-phenyl-1H-indole-3- carboxamide* (**L–12**). The title product was obtained from **7l** and 3-(amino- methyl)-4,6-dimethylpyridin-2(1*H*)-one hydrochloride according to the general procedure described above as a white solid (0.15 g, 38%). M.p. 285–287 °C. ^1^H-NMR (DMSO-*d_6_*) δ 11.56 (brs, 1H, NH_pyridone_), 8.94 (s, 1H, OH), 7.62–7.59 (m, 2H, ArH), 7.54–7.50 (m, 2H, ArH), 7.40 (d, *J* = 7.3 Hz, 2H, ArH), 7.18 (d, *J* = 2.1 Hz, 1H), 6.78 (d, *J* = 8.7 Hz, 1H, ArH), 6.61–6.59 (dd, *J* = 8.7 Hz, 2.2 Hz, 1H, ArH), 5.90 (s, 1H, ArH_pyridone_), 4.35 (d, *J* = 5.4 Hz, 2H, NHCH_2_), 2.38 (s, 3H, CH_3_), 2.27 (s, 3H, CH_3_), 2.12 (s, 3H, CH_3_). ^13^C-NMR (DMSO-*d_6_*) δ 165.3 (C=O_indole_), 163.9 (C=O_pyridone_), 152.9 (ArCH_pyridone_), 149.0 (ArCH_indole_), 143.1 (ArCH_pyridone_), 140.3 (N-C=C_indole_), 137.0 (PhCH), 131.7 (ArCH_indole_), 130.2 (PhCH), 128.8 (ArCH_indole_), 128.4 (PhCH), 126.7 (ArCH_pyridone_), 122.9 (PhCH), 122.1 (ArCH_indole_), 111.0 (N-C=C_indole_), 109.1 (ArCH_indole_), 108.1 (ArCH_pyridone_), 104.4 (ArCH_indole_), 35.6 (NHCH_2_), 19.4 (CH_3pyridone_), 18.7 (CH_3pyridone_), 13.0 (CH_3indole_). ESI-HRMS: m/z [M+Na]^+^ calcd for C_24_H_23_O_3_N_3_Na: 424.1632; found: 424.1662.

*N-((4,6-Dimethyl-2-oxo-1,2-dihydropyridin-3-yl)methyl)-1-(furan-2-ylmethyl)-5-hydroxy-2-methyl-1H-indole-3-carboxamide* (**L–13**). The title product was obtained from **7m** and 3-(amino- methyl)-4,6-dimethylpyridin-2(1*H*)-one hydrochloride according to the general procedure described above as a white solid (0.30 g, 75%). M.p. 290–292 °C. ^1^H-NMR (DMSO-*d_6_*) δ 11.54 (s, 1H, NH_pyridone_), 8.85 (s, 1H, OH), 7.53 (s, 1H, NHCH_2_), 7.39 (dd, *J* = 13.6 Hz, 5.2 Hz, 2H, ArH), 7.08 (d, *J* = 2.0 Hz, 1H, ArH), 6.64-6.63 (dd, *J* = 8.6 Hz, 2.0 Hz, 1H), 6.40–6.36 (m, 2H, ArH), 5.88 (s, 1H, ArH_pyridone_), 5.32 (s, 2H, NCH_2_), 4.31 (d, *J* = 5.3 Hz, 2H, NHCH_2_), 2.64 (s, 3H, CH_3_), 2.25 (s, 3H, CH_3_), 2.11 (s, 3H, CH_3_). ^13^C-NMR (DMSO-*d_6_*) δ 165.3 (C=O_indole_), 163.9 (C=O_pyridone_), 152.5 (ArCH_pyridone_), 151.0 (ArCH_furan_), 148.9 (ArCH_indole_), 143.3 (ArCH_pyridone_), 143.1 (ArCH_furan_), 140.5 (N-C=C_indole_), 130.4 (ArCH_furan_), 126.5 (ArCH_indole_), 122.9 (ArCH_furan_), 111.5 (ArCH_indole_), 111.0 (N-C=C_indole_), 108.6 (ArCH_indole_), 108.1 (ArCH_pyridone_), 108.0 (ArCH_indole_), 104.3 (ArCH_indole_), 46.2 (NCH_2_), 35.5 (NHCH_2_), 19.4 (CH_3pyridone_), 18.6 (CH_3pyridone_), 11.9 (CH_3indole_). ESI-HRMS: m/z [M+Na]^+^ calcd for C_23_H_23_O_4_N_3_Na: 428.1581; found: 428.1587.

*1-Cyclohexyl-N-((4,6-dimethyl-2-oxo-1,2-dihydropyridin-3-yl)methyl)-5-hydroxy-2-methyl-1H-indole-3-carboxamide* (**L–14**). The title product was obtained from **7n** and 3-(amino- methyl)-4,6-dimethylpyridin-2(1*H*)-one hydrochloride according to the general procedure described above as a white solid (0.36 g, 90%). M.p. 268–270 °C. ^1^H-NMR (DMSO-*d_6_*) δ 11.53 (s, 1H, NH_pyridone_), 8.80 (s, 1H, OH), 7.44 (t, *J* = 5.3 Hz, 1H, NHCH_2_), 7.32 (t, *J* = 5.3 Hz, 1H, ArH), 7.05 (d, *J* = 1.9 Hz, 1H, ArH), 6.60–6.58 (dd, *J* = 2.0 Hz, 1H, ArH), 5.88 (s, 1H, ArH_pyridone_), 4.31 (d, *J* = 5.3 Hz, 2H, NHCH_2_), 4.20 (brs, 1H, NCH), 2.57 (s, 3H, CH_3_), 2.25 (s, 3H, CH_3_), 2.11 (s, 3H, CH_3_), 2.19–2.17 (m, 2H, CH_2_), 1.86 (d, *J* = 13.0 Hz, 2H, CH_2_), 1.74–1.67 (m, 3H, CH_2_+1/2CH_2_), 1.48–1.30 (m, 3H, CH_2_+1/2CH_2_). ^13^C-NMR (DMSO-*d_6_*) δ 165.7 (C=O_indole_), 163.9 (C=O_pyridone_), 151.8 (ArCH_pyridone_), 148.9 (ArCH_indole_), 143.0 (ArCH_pyridone_), 140.1 (N-C=C_indole_), 129.0 (ArCH_indole_), 127.5 (ArCH_pyridone_), 122.9 (ArCH_indole_), 113.1 (ArCH_indole_), 111.0 (N-C=C_indole_), 108.1 (ArCH_pyridone_), 107.9 (ArCH_indole_), 104.2 (ArCH_indole_), 55.2 (NCH), 35.5 (NHCH_2_), 31.0 (CH_2cyclohexyl_), 26.2 (CH_2cyclohexyl_), 25.3 (CH_2cyclohexyl_), 19.4 (CH_3pyridone_), 18.6 (CH_3pyridone_), 12.5 (CH_3indole_). ESI-HRMS: m/z [M+Na]^+^ calcd for C_24_H_29_O_3_N_3_Na: 430.2101; found: 430.2116.

*N-((4,6-Dimethyl-2-oxo-1,2-dihydropyridin-3-yl)methyl)-5-hydroxy-1-isopropyl-2-methyl-1H-indole-3-carboxamide* (**L–15**). The titled product was obtained from **7o** and 3-(amino- methyl)-4,6-dimethylpyridin-2(1*H*)-one hydrochloride according to the general procedure described above as a white solid (0.24 g, 65%). M.p. 234–236 °C. ^1^H-NMR (DMSO-*d_6_*) δ 11.53 (s, 1H, NH_pyridone_), 8.80 (s, 1H, OH), 7.39 (d, *J* = 8.8 Hz, 1H, ArH), 7.32 (t, *J* = 5.2 Hz, 1H, NHCH_2_), 7.06 (d, *J* = 2.1 Hz, 1H, ArH), 6.61–6.59 (dd, *J* = 8.8 Hz, 2.2 Hz, 1H, ArH), 5.88 (s, 1H, ArH_pyridone_), 4.71–4.66 (m, 1H, NCH), 4.31 (d, *J* = 5.3 Hz, 2H, NHCH_2_), 2.56 (s, 3H, CH_3_), 2.25 (s, 3H, CH_3_), 2.11 (s, 3H, CH_3_), 1.50 (d, *J* = 7.0 Hz, 6H, 2 × CH_3_). ^13^C-NMR (DMSO-*d_6_*) δ 165.6 (C=O_indole_), 163.8 (C=O_pyridone_), 151.9 (ArCH_pyridone_), 148.9 (ArCH_indole_), 143.0 (ArCH_pyridone_), 139.9 (N-C=C_indole_), 128.7 (ArCH_indole_), 127.5 (ArCH_indole_), 122.9 (ArCH_indole_), 112.5 (ArCH_indole_), 111.1 (N-C=C_indole_), 108.1 (ArCH_pyridone_), 107.7 (ArCH_pyridone_), 104.3 (ArCH_indole_), 46.8 (NCH), 35.5 (NHCH_2_), 21.5 (CH_3_), 19.4 (CH_3pyridone_), 18.6 (CH_3pyridone_), 12.4 (CH_3indole_). ESI-HRMS: m/z [M+Na]^+^ calcd for C_21_H_25_O_3_N_3_Na: 390.1788; found: 390.1821.

*N-((4,6-Dimethyl-2-oxo-1,2-dihydropyridin-3-yl)methyl)-1-isopropyl-2-methyl-5-(3-(piperidin-1-yl)- propoxy)-1H-indole-3-carboxamide* (**L–16**). The title product was obtained from **7e** and **3** according to the general procedure described above as a white solid (80 mg, 40%). M.p. 200–202 °C. ^1^H-NMR (CDCl_3_) δ 12.06 (brs, 1H, NH_pyridone_), 7.46 (t, *J* = 5.7 Hz, 1H, NHCH_2_), 7.34–7.32 (m, 2H, ArH), 6.73 (dd, *J* = 7.1 Hz, 2.2 Hz, 1H, ArH), 5.87 (s, 1H, ArH_pyridone_), 4.70–4.65 (m, 1H, NCH), 4.58 (d, *J* = 6.0 Hz, 2H, NHCH_2_), 4.12 (t, *J* = 6.6 Hz, 2H, OCH_2_), 2.72 (brs, 2H, CH_2_), 2.71 (s, 3H, CH_3_), 2.60 (brs, 4H, CH_2_NCH_2_), 2.41 (s, 3H), 2.23 (s, 3H, CH_3_), 2.08–2.06 (m, 2H, CH_2_), 1.71–1.70 (m, 4H, 2 × CH_2_), 1.58 (d, *J* = 7.0 Hz, 6H, 2 × CH_3_), 1.48 (brs, 2H, CH_2_). ^13^C-NMR (CDCl_3_) δ 165.9 (C=O_indole_), 165.0 (C=O_pyridone_), 153.8 (ArCH_pyridone_), 149.3 (ArCH_indole_), 142.3 (ArCH_pyridone_), 141.0 (N-C=C_indole_), 129.3 (ArCH_indole_), 126.7 (ArCH_indole_), 123.8 (ArCH_indole_), 112.3 (ArCH_indole_), 111.6 (N-C=C_indole_), 109.5 (ArCH_pyridone_), 108.0 (ArCH_pyridone_), 102.2 (ArCH_indole_), 66.9 (CH_2_O), 55.8 (NCH_2_CH_2_CH_2_O), 54.2 (CH_2_NCH_2_), 46.9 (NCH), 35.6 (NHCH_2_), 26.1 (NCH_2_CH_2_CH_2_O), 24.8 (CH_2piperidine_), 23.7 (CH_2piperidine_), 21.4 (CH_3_), 19.6 (CH_3pyridone_), 19.0 (CH_3pyridone_), 12.1 (CH_3indole_). ESI-HRMS: m/z [M+H]^+^ calcd for C_29_H_41_O_3_N_4_: 493.3173; found: 493.3174.

*1-Isopropyl-N-((4-methoxy-6-methyl-2-oxo-1,2-dihydropyridin-3-yl)methyl)-2-methyl-5-(3-(piperidin-1-yl)propoxy)-1H-indole-3-carboxamide* (**L–17**). The title product was obtained from **7e** and pyridone **25** according to the general procedure described above as a white solid (100 mg, 48%). M.p. 181–188 °C. ^1^H-NMR (CDCl_3_) δ 7.66 (t, *J* = 5.0 Hz, 1H, NHCH_2_), 7.42 (d, *J* = 1.9 Hz, 1H, ArH), 7.35 (d, *J* = 8.9 Hz, 1H, ArH), 6.74–6.72 (dd, *J* = 8.9 Hz, 2.2 Hz, 1H, ArH), 5.90 (s, 1H, ArH_pyridone_), 4.71–4.67 (m, 1H, NCH), 4.63 (d, *J* = 5.5 Hz, 2H, OCH_2_), 4.15 (t, *J* = 6.1 Hz, 2H, NCH_2_), 3.88 (s, 3H, OCH_3_), 2.90 (brs, 2H, CH_2_), 2.74–2.62 (m, 6H, CH_2_), 2.32 (s, 3H, CH_3_), 2.14 (t, *J* = 6.8 Hz, 2H, CH_2_), 1.77 (brs, 4H, 2 × CH_2_), 1.59 (d, *J* = 7.0 Hz, 6H, 2 × CH_3_), 1.50 (brs, 2H, CH_2_). ^13^C-NMR (CDCl_3_) δ 165.8 (C=O_indole_), 165.7 (ArCOCH_3pyridone_), 165.4 (C=O_pyridone_), 153.5 (ArCH_pyridone_), 146.0 (ArCH_indole_), 141.5 (N-C=C_indole_), 129.5 (ArCH_indole_), 126.7 (ArCH_indole_), 112.3 (ArCH_indole_), 111.4 (N-C=C_indole_), 108.2 (ArCH_pyridone_), 107.5 (ArCH_indole_), 103.0 (ArCH_indole_), 94.5 (ArCH_pyridone_), 66.8 (CH_2_O), 56.1 (OCH_3_), 55.6 (NCH_2_CH_2_CH_2_O), 53.9 (CH_2_NCH_2_), 46.9 (NCH), 32.7 (NHCH_2_), 25.4 (NCH_2_CH_2_CH_2_O), 24.1 (CH_2piperidine_), 23.2 (CH_2piperidine_), 21.4 (CH_3_), 19.5 (CH_3pyridone_), 12.2 (CH_3indole_). ESI-HRMS: m/z [M+H]^+^ calcd for C_29_H_41_O_4_N_4_: 509.3122; found: 509.3127.

*1-Isopropyl-2-methyl-N-((2-oxo-2,5,6,7-tetrahydro-1H-cyclopenta[b]pyridin-3-yl)methyl)-5-(3-(piperidin-1-yl)propoxy)-1H-indole-3-carboxamide* (**L–18**). The title product was obtained from **7e** and pyridone **17** according to the general procedure described above as a white solid (65 mg, 31%). M.p. 141–144 °C. ^1^H-NMR (CDCl_3_) δ 7.45 (s, 1H, OH), 7.43 (t, *J* = 5.5 Hz, 1H, NHCH_2_), 7.35–7.33 (m, 2H, ArH), 6.73–6.71 (dd, *J* = 8.9 Hz, 1.9 Hz, 1H, ArH), 4.70–4.66 (m, 1H, NCH), 4.50 (d, *J* = 5.9 Hz, 2H, OCH_2_), 4.20 (t, *J* = 6.7 Hz, 2H, NCH_2_), 2.95 (brs, 2H, CH_2_), 2.85 (t, *J* = 7.2 Hz, 2H, CH_2_), 2.82 (brs, 2H, CH_2_), 2.71 (s, 3H, CH_3_), 2.68 (t, *J* = 7.3 Hz, 2H, CH_2_), 2.22 (t, *J* = 6.4 Hz, 2H, CH_2_), 2.10–2.05 (m, 2H, CH_2_), 1.82 (brs, 4H, 2 × CH_2_), 1.58 (d, *J* = 7.0 Hz, 6H, 2 × CH_3_), 1.55 (brs, 2H, CH_2_). ^13^C-NMR (CDCl_3_) δ 166.0 (C=O_indole_), 164.5 (C=O_pyridone_), 153.7 (ArCH_pyridone_), 147.9 (ArCH_indole_), 141.3 (ArCH_pyridone_), 137.2 (N-C=C_indole_), 129.3 (ArCH_indole_), 126.6 (ArCH_indole_), 126.3 (ArCH_indole_), 119.0 (ArCH_indole_), 112.4 (N-C=C_indole_), 111.8 (ArCH_pyridone_), 108.1 (ArCH_pyridone_), 102.2 (ArCH_indole_), 66.9 (CH_2_O), 55.6 (NCH_2_CH_2_CH_2_O), 54.0 (CH_2_NCH_2_), 46.9 (NCH), 40.5 (NHCH_2_), 31.5 (CH_2pyridone_), 29.6 (CH_2pyridone_), 25.6 (NCH_2_CH_2_CH_2_O), 24.2 (CH_2piperidine_), 23.3 (CH_2piperidine_), 23.0 (CH_2pyridone_), 21.4 (CH_3_), 12.0 (CH_3indole_). ESI-HRMS: m/z [M+H]^+^ calcd for C_30_H_41_O_3_N_4_: 505.3173; found: 505.3188.

*N-((6-Ethyl-4-methyl-2-oxo-1,2-dihydropyridin-3-yl)methyl)-1-isopropyl-2-methyl-5-(3-(piperidin-1-yl)propoxy)-1H-indole-3-carboxamide* (**L–19**). The title product was obtained from **7e** and pyridone **22** according to the general procedure described above as a yellow solid (75 mg, 36%). M.p. 180–182 ^o^C. ^1^H-NMR (CDCl_3_) δ 7.42 (t, *J* = 5.5 Hz, 1H, NHCH_2_), 7.34–7.33 (m, 2H, ArH), 6.74–6.72 (dd, *J* = 8.9 Hz, 1.9 Hz, 1H, ArH), 5.90 (s, 1H, ArH_pyridone_), 4.71–4.64 (m, 1H, NCH), 4.59 (d, *J* = 6.1 Hz, 2H, OCH_2_), 4.10 (t, *J* = 6.2 Hz, 2H, NCH_2_), 2.71 (s, 3H, CH_3_), 2.69 (brs, 2H, CH_2_), 2.55 (brs, 4H, 2 × CH_2_), 2.52 (q, *J* = 7.6 Hz, 2H, CH_2_), 2.43 (s, 3H), 2.03 (brs, 2H, CH_2_), 1.67 (brs, 4H, 2 × CH_2_), 1.58 (s, 6H, 2 × CH_3_), 1.45 (brs, 2H, CH_2_), 1.18 (t, *J* = 7.5 Hz, 3H, CH_3_). ^13^C-NMR (CDCl_3_) δ 165.9 (C=O_indole_), 165.1 (C=O_pyridone_), 153.9 (ArCH_pyridone_), 149.5 (ArCH_indole_), 148.1 (ArCH_pyridone_), 141.0 (N-C=C_indole_), 129.3 (ArCH_indole_), 126.7 (ArCH_indole_), 123.7 (ArCH_indole_), 112.3 (ArCH_indole_), 111.5 (N-C=C_indole_), 108.0 (ArCH_pyridone_), 107.8 (ArCH_pyridone_), 102.4 (ArCH_indole_), 66.9 (CH_2_O), 55.9 (NCH_2_CH_2_CH_2_O), 54.2 (CH_2_NCH_2_), 46.9 (NCH), 35.7 (NHCH_2_), 26.1 (NCH_2_CH_2_CH_2_O), 26.0 (CH_2_CH_3pyridone_), 24.9 (CH_2piperidine_), 23.7 (CH_2piperidine_), 21.4 (CH_3_), 19.8 (CH_3pyridone_), 12.7 (CH_2_CH_3pyridone_), 12.2 (CH_3indole_). ESI-HRMS: m/z [M+H]^+^ calcd for C_30_H_43_O_3_N_4_: 507.3330; found: 507.3330.

*1-Isopropyl-2-methyl-N-((2-oxo-1,2,5,6,7,8-hexahydroquinolin-3-yl)methyl)-5-(3-(piperidin-1-yl)propoxy)-1H-indole-3-carboxamide* (**L–20**). The title product was obtained from **7e** and pyridone **16** according to the general procedure described above as a white solid (52 mg, 25%). M.p. 127–130 °C. ^1^H-NMR (CDCl_3_) δ 11.61 (brs, 1H, NH_pyridone_), 7.41 (brs, 2H, ArH), 7.36–7.34 (m, 2H, ArH), 6.74–6.73 (dd, *J* = 8.9 Hz, 1.9 Hz, 1H, ArH), 4.73–4.67 (m, 1H, NCH), 4.49 (d, *J* = 5.8 Hz, 2H, OCH_2_), 4.21 (t, *J* = 5.8 Hz, 2H, NCH_2_), 3.06 (brs, 2H, CH_2_), 2.86 (brs, 3H, CH_2_), 2.73 (s, 3H, CH_3_), 2.65 (brs, 2H, CH_2_), 2.46–2.45 (m, 3H, CH_2_), 2.25 (brs, 2H, CH_2_), 1.85 (brs, 4H, CH_2_), 1.71–1.70 (m, 5H, CH_2_), 1.61 (brs, 3H, CH_2_), 1.59 (d, *J* = 7.0 Hz, 6H, 2 × CH_3_). ^13^C-NMR (CDCl_3_) δ 165.9 (C=O_indole_), 163.7 (C=O_pyridone_), 153.6 (ArCH_pyridone_), 141.9 (ArCH_indole_), 141.6 (ArCH_pyridone_), 141.5 (N-C=C_indole_), 129.5 (ArCH_indole_), 126.6 (ArCH_indole_), 125.9 (ArCH_pyridone_), 113.9 (ArCH_indole_), 112.4 (ArCH_indole_), 111.8 (N-C=C_indole_), 107.9 (ArCH_pyridone_), 102.9 (ArCH_indole_), 67.0 (CH_2_O), 55.6 (NCH_2_CH_2_CH_2_O), 53.8 (CH_2_NCH_2_), 46.9 (NCH), 40.2 (NHCH_2_), 29.7 (CH_2pyridone_), 26.7 (CH_2pyridone_), 26.1 (NCH_2_CH_2_CH_2_O), 23.8 (CH_2piperidine_), 22.4 (CH_2pyridone_), 21.7 (CH_2pyridone_), 21.4 (CH_3_), 12.1 (CH_3indole_). ESI-HRMS: m/z [M+H]^+^ calcd for C_31_H_43_O_3_N_4_: 519.3330; found: 519.3329.

*N-((6-Isobutyl-2-oxo-1,2-dihydropyridin-3-yl)methyl)-1-isopropyl-2-methyl-5-(3-(piperidin-1-yl)propoxy)-1H-indole-3-carboxamide* (**L–21**). The title product was obtained from **7e** and pyridone **20** according to the general procedure described above as a white solid (26 mg, 12%). M.p. 99–101 °C. ^1^H-NMR (CDCl_3_) δ 12.23 (brs, 1H, NH_pyridone_), 7.47 (d, *J* = 6.9 Hz, 1H, ArH), 7.37–7.35 (m, 2H, ArH), 7.31 (t, *J* = 5.5 Hz, 1H, ArH), 6.75–6.74 (dd, *J* = 8.9 Hz, 2.0 Hz, 1H, ArH), 6.00 (d, *J* = 6.1 Hz, 1H, ArH_pyridone_), 4.71–4.65 (m, 1H, NCH), 4.53 (d, *J* = 5.8 Hz, 2H, OCH_2_), 4.09 (t, *J* = 6.1 Hz, 2H, NCH_2_), 2.74 (brs, 2H, CH_2_), 2.72 (s, 3H, CH_3_), 2.61 (brs, 4H, 2 × CH_2_), 2.40 (d, *J* = 7.2 Hz, 2H, CH_2_), 2.08–2.07 (m, 2H, CH_2_), 2.00–1.93 (m, 1H, CH), 1.71 (brs, 4H, 2 × CH_2_), 1.59 (d, *J* = 7.0 Hz, 6H, 2 × CH_3_), 1.47 (brs, 2H, CH_2_), 0.85 (d, *J* = 6.6 Hz, 6H, 2 × CH_3_). ^13^C-NMR (CDCl_3_) δ 166.1 (C=O_indole_), 165.0 (C=O_pyridone_), 153.8 (ArCH_pyridone_), 148.1 (ArCH_pyridone_), 141.1 (N-C=C_indole_), 139.5, 129.4 (ArCH_indole_), 126.8 (ArCH_indole_), 125.7 (ArCH_indole_), 111.5 (N-C=C_indole_), 108.0 (ArCH_pyridone_), 105.9 (ArCH_pyridone_), 102.7 (ArCH_indole_), 66.9 (CH_2_O), 55.8 (NCH_2_CH_2_CH_2_O), 54.1 (CH_2_NCH_2_), 46.9 (NCH), 42.1 (NHCH_2_), 39.9 (CH_2_CH), 29.7 (CH(CH_3_)_2_), 28.5 (CH_2piperidine_), 25.9 (NCH_2_CH_2_CH_2_O), 24.7 (CH_2piperidine_), 23.6 (CH_2piperidine_), 22.1 (CH(CH_3_)_2_), 21.4 (CH_3_), 12.2 (CH_3indole_). ESI-HRMS: m/z [M+H]^+^ calcd for C_31_H_45_O_3_N_4_: 521.3486; found: 521.3488.

*1-Isopropyl-2-methyl-N-((4-methyl-2-oxo-1,2,5,6,7,8-hexahydroquinolin-3-yl)methyl)-5-(3-(piperidin-1-yl)propoxy)-1H-indole-3-carboxamide* (**L–22**). The title product was obtained from **7e** and pyridone **15** according to the general procedure described above as a white solid (38 mg, 17%). M.p. 194–196 °C. ^1^H-NMR (CDCl_3_) δ 13.10 (brs, 1H, NH_pyridone_), 7.55 (t, *J* = 5.8 Hz, 1H, NHCH_2_), 7.34–7.31 (m, 2H, ArH), 6.74–6.72 (dd, *J* = 8.9 Hz, 2.2 Hz, 1H, ArH), 4.70–4.65 (m, 1H, NCH), 4.63 (d, *J* = 5.9 Hz, 2H, OCH_2_), 4.00 (t, *J* = 6.4 Hz, 2H, NCH_2_), 3.02 (t, *J* = 5.9 Hz, 2H, CH_2_), 2.71 (s, 3H, CH_3_), 2.42–2.34 (m, 8H, 4 × CH_2_), 2.14 (s, 3H, CH_3_), 1.86–1.84 (m, 2H, CH_2_), 1.75–1.71 (m, 4H, 2 × CH_2_), 1.58 (d, *J* = 7.0 Hz, 6H, 2 × CH_3_), 1.56–1.54 (m, 4H, 2 × CH_2_), 1.39 (brs, 2H, CH_2_). ^13^C-NMR (CDCl_3_) δ 165.9 (C=O_indole_), 163.7 (C=O_pyridone_), 154.0 (ArCH_pyridone_), 150.1 (ArCH_pyridone_), 140.7 (N-C=C_indole_), 140.4 (ArCH_indole_), 130.9 (ArCH_indole_), 128.8 (ArCH_indole_), 126.8 (ArCH_indole_), 122.8 (ArCH_pyridone_), 114.4 (ArCH_indole_), 111.3 (N-C=C_indole_), 108.1 (ArCH_pyridone_), 102.3 (ArCH_indole_), 66.9 (CH_2_O), 56.0 (NCH_2_CH_2_CH_2_O), 54.4 (CH_2_NCH_2_), 46.9 (NCH), 35.0 (NHCH_2_), 27.4 (NCH_2_CH_2_CH_2_O), 26.7 (CH_2pyridone_), 25.5 (CH_2piperidine_), 25.1 (CH_2piperidine_), 24.2 (CH_2piperidine_), 22.4 (CH_2pyridone_), 22.3 (CH_2pyridone_), 21.4 (CH_3_), 19.2 (CH_3pyridone_), 16.7 (CH_2pyridone_), 12.2 (CH_3indole_). ESI-HRMS: m/z [M+H]^+^ calcd for C_32_H_45_O_3_N_4_: 533.3486; found: 533.3495.

#### 3.2.8. React IR Experiment

The ReactIR 15 DiComp probe was inserted in a 50 mL, three-neck flask equipped with a magnetic stirrer. IR spectra were obtained every 15 s. Data collection began at the start of the experiment. The solution of ethyl acetoacetate (1.0 g, 7.68 mmol) and TCCA (34.8 mg, 0.15 mmol) in MeCN (5 mL) was cooled to 0 °C, and benzylamine (0.82 g, 7.68 mmol) was added dropwise. The resulting solution was stirring under ice bath and naturally elevated to at room temperature for indicated time. After the reaction was completed, the workup was consistent with the general procedure 3.1.1.

### 3.3. In Vitro Biological Activity Assays

#### 3.3.1. Cell Growth Inhibition Assay 

The human chronic myeloid leukemia cell line K562 was purchased from the American Type Culture Collection (ATCC, Perry Pkwy, Gaithersburg, USA). The cells were cultured in RPMI 1640 medium supplemented with 10% fetal bovine serum, 1% penicillin and streptomycin at 37 °C in a humid atmosphere containing 5% CO_2_ in air. In the cell growth experiments, cells were seeded with 5000 cells/well density in the culture medium containing 100 μL diluent series of test compounds (100, 10, 1, 0.1 μM) in 96 well cell culture plates. After treatment for 48 hours, cell growth was measured by WST-8 assay based on lactate dehydrogenase using the Spectramax Paradox Multimode detection platform. The CCK-8 reagent was added to each well of 10 μL, and the cells were incubated for another 1–4 hours and read at 450 nm. The readings were standardized relative to the cells treated by the carrier, and IC_50_ was calculated by nonlinear regression analysis using SPSS statistics 20.0 software (IBM, Chicago, Michigan, USA).

#### 3.3.2. Western Blot Analysis 

The cells were treated with test compounds (10 and 5 μM), and the extract was prepared by adding radio immunoprecipitation assay (RIPA) lysis buffer to the cells under ice bath. The protein concentrations were determined by the bicinchoninic acid (BCA) assay. Protein samples were separated by sodium lauryl sulfate-polyacrylamide gel electrophoresis (SDS-PAGE) and transferred to a polyvinylidene fluoride (PVDF) membrane. Cell membranes were blocked with 5% milk for 1 hour at room temperature and overnight at 4 °C with designated primary antibodies. After washing with tris buffered saline Tween (TBST) three times for 30 min, incubated with horseradish peroxidase-labeled secondary antibody for 1 h at room temperature, and then washing with TBST three times for 30 min to enhance chemiluminescence. The band intensity in Figure 3 was quantified by the Odyssey software (Li-Cor, Lincoln, Nebraska, USA) and histone H3 was used as a housekeeping protein for normalization.

### 3.4. Molecular Docking

We processed EZH2 protein (PDB code: 4W2R) using the protein preparation wizard in Schrödinger suite [40,41,42]. First, the complex’s protonation states were adjusted for consistency with pH 7.4. Second, all hydrogen atoms and the total structure for the protein and small molecules were evaluated for energy accuracy using an all-atom force field, OPLS_2005; restrained minimization; and heavy atom convergence to a 0.3 Å RMSD. Third, the above prepared EZH2 protein and co-crystallized molecule were used to generate a receptor grid file. The position of co-crystallized molecule was used to determine the active site location (x = 30.57, y = 18.29, and z = 54.62) and size (inner box = 10 Å × 10 Å × 10 Å; outer box = 20 Å × 20 Å × 20 Å). The grid was generated using the OPLS_2005 force field. The glide docking was then carried out between the prepared protein and molecules. The docking result was exported and analyzed using Discovery Studio 2019 and PyMol. 

## 4. Conclusions

In summary, we have synthesized a series of 5-hydroxyindole scaffold compounds bearing as main pharmacophore a pyridone moiety. According to molecular modeling and in vitro biological activity assays, the preliminary structure-activity relationships were determined and summarized. Compound **L–04** improved both the H3K27Me3 reduction and antiproliferation parameters (IC_50_ = 52.6 μM) on K562 cells. It bears mentioning that **L–21** had comparable inhibitory effect (IC_50_ = 51.8 μM) to **L–04**; however, molecular docking indicated that **L–21** (with the N1 position substituted with an isopropyl group) had reduced binding energy due to the absence of π-π interactions created by the benzyl group. In all, we believe that **L–04** should remain a potential candidate to design more EZH2Is, and future in vivo studies using patient-derived tumor xenograft models are required to further assess the utility of this compound. 

During the preparation of compounds, we discovered a novel catalytic activity of TCCA in condensation reactions. Different amines were condensed with β-diketones or β-ketoesters in the presence of TCCA to afford the corresponding product in short times with high yields, which displayed some significant advantages and provided an alternative condensation strategy. In addition, we established a model for **L–04** binding to EZH2 using molecular docking, which will be utilized to direct future analogue design and synthesis. These findings further support the therapeutic potential of EZH2Is as anti-cancer agents.

## Figures and Tables

**Table 1:** Representative EZH2Is in clinical trials.

Compound	Structure	Highest Phase	Tumor Type	Organization
GSK–126 [24]	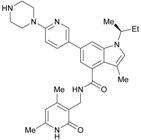	Discontinued	Solid tumors, relapsed and refractory diffuse large B-cell lymphoma (DLBCL)	GlaxoSmithKline
Tazemetostat (EPZ-6438) [25]	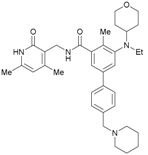	Launched	Metastatic / local advanced epithelioid sarcoma	Epizyme
CPI–1205 [26]	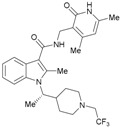	Phase I/II	Metastatic castration-resistant prostate cancer (mCRPC)	Constellation Pharmaceuticals
Valemetostat (DS–3201) [27]	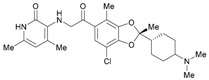	Phase I	Acute myeloid and lymphocytic leukemia	Daiichi Sankyo
PF–06821497 [28]	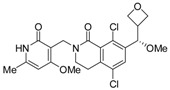	Phase I	Elapsed or refractory small cell lung cancer (SCLC), CRPC, DLBCL and FL	Pfizer
SHR–2554 [29]	undisclosed	Phase I	Relapsed or refractory mature lymphoid neoplasms	Jiangsu Hengrui

**Table 2:**
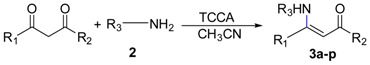
Scope with respect to substrates using TCCA as a catalyst ^a.^

Entry	R_1_	R_2_	R_3_	Product	Time (min)	Yield (%) ^b^
1	Me	OEt	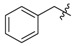	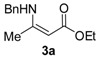	10	95
2	Me	OEt	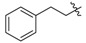	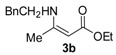	10	98
3	Me	OEt	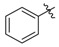	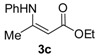	20	92
4	Me	OEt	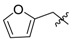	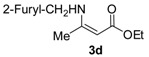	10	94
5	Me	OEt	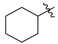	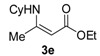	10	95
6	Me	OEt	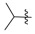	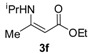	15	96
7	Me	OEt	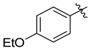	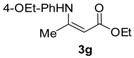	40	95
8	Me	OEt	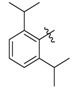	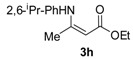	60	90
9	Me	OEt	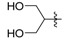	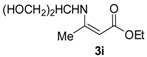	15	94
10	Me	OtBu	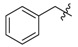	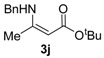	10	99
11	Me	OtBu	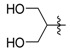	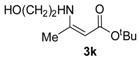	10	94
12	Me	OtBu	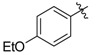	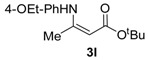	40	93
13	Me	OtBu	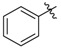	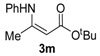	40	92
14	Me	Me	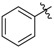	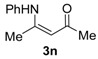	40	90
15	Me	Me	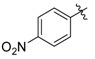	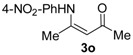	60	86
16	Ph	OEt	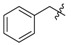	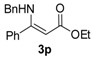	50	92

^a^ Unless otherwise noted, β-diketones or β-ketoesters (7.68 mmol), amine (7.68 mmol), TCCA (0.15 mmol) and CH_3_CN (5 mL) were used; the reactions were conducted under ice bath and naturally elevated to room temperature. ^b^ Crude yield.

**Table 3:** Antiproliferative effects of 5-hydroxyindole-based inhibitors against K562 cells.

Compd.	IC50 (μM) ± SEM ^a^	cLogP ^b^	Compd.	IC50 (μM) ± SEM ^a^	cLogP ^b^
**L–01**	83.8 ± 1.8	3.94	**L–13**	>100	3.53
**L–02**	72.6 ± 1.6	5.10	**L–14**	63.9 ± 3.2	4.40
**L–03**	86.1 ± 2.7	4.33	**L–15**	56.3 ± 1.7	3.47
**L–04**	52.6 ± 0.4	5.10	**L–16**	>100	5.02
**L–05**	71.1 ± 3.2	5.49	**L–17**	>100	4.72
**L–06**	>100	4.80	**L–18**	>100	4.89
**L–07**	>100	5.02	**L–19**	68.1 ± 1.2	5.27
**L–08**	>100	4.63	**L–20**	>100	5.28
**L–09**	>100	3.94	**L–21**	51.8 ± 1.6	5.60
**L–10**	58.7 ± 3.8	4.13	**L–22**	>100	5.59
**L–11**	55.2 ± 1.8	5.29	**Tazemetostat**	59.2 ± 0.6	4.73
**L–12**	85.5 ± 1.0	3.88			

^a^ IC_50_: 50% inhibitory concentration (determined by standard CCK-8 assay) after treatment of 48 h. Each experiment was carried out in triplicate. ^b^ Predicted by pkCSM [39].

## Supplementary Materials

The following are available online, ^1^H-NMR and HR-MS spectra of β-aminopropenones and β-aminopropionates; Preparation of pyridone derivatives; ^1^H-NMR, ^13^C-NMR and HR-MS spectra of intermediates and EZH2 inhibitors; Table S1: Reaction conditions.
