# Supplementary material for: 5-Hydroxyindole-Based EZH2 Inhibitors Assembled via TCCA-Catalyzed Condensation and Nenitzescu Reactions
Source: Molecules. 2020 Apr 28;25(9):2059. doi: 10.3390/molecules25092059 (PMC7248849; doi:10.3390/molecules25092059)
Supplement: Supplementary file 1 [file molecules-25-02059-s001.pdf]

5-Hydroxyindole-based EZH2 Inhibitors Assembled via TCCA-catalyzed Condensation and Nenitzescu Reactions

Fangyu Du [1,†], Qifan Zhou [1,†], Wenjiao Sun [1], Cheng Yang [2], Chunfu Wu [2], Lihui Wang [2,*] and Guoliang Chen [1,*]

[1] Key Laboratory of Structure-Based Drug Design & Discovery of Ministry of Education, School of Pharmaceutical Engineering, Shenyang Pharmaceutical University, No. 103 Wenhua Road, Shenhe District, Shenyang 110016, China
[2] Department of Pharmacology, Shenyang Pharmaceutical University, No. 103 Wenhua Road, Shenhe District, Shenyang 110016, China
[†] Authors contributed equally to this work.
[*] Correspondence: chenguoliang@syphu.edu.cn, (G.C.); lhwang@syphu.edu.cn, (L.W.)

Table of contents

Table S1. Identification of reaction conditions 2

Spectrum of β-aminopropenones and β-aminopropionates 3

Preparation of pyridone derivatives 18

Spectrum of intermediates and EZH2 inhibitors 24

Table S1. Identification of reaction conditions [a]

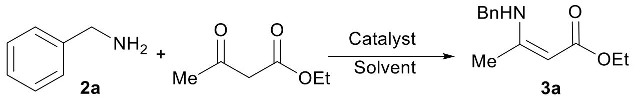

Entry	Solvent	Catalyst (mol%)	Time (min/[h])	Yield (%) [b]
1	--	--	[24]	56
2	DCM	HOAc (10)	[15]	85
3	DCM	TCCA (2.0)	30	50
4	EA	TCCA (2.0)	35	48
5	THF	TCCA (2.0)	50	--
6	CH_3CN	TCCA (2.0)	20	93
7	CH_3CN	TCCA (0.2)	60	43
8	CH_3CN	TCCA (0.5)	60	57
9	CH_3CN	TCCA (1.0)	60	65
10	CH_3CN	TCCA (1.5)	10	86
11	CH_3CN	TCCA (3.0)	10	95
12 [c]	CH_3CN	TCCA (2.0)	20	94

[a] Unless otherwise noted, ethyl acetoacetate (0.5 g, 3.84 mmol) and benzylamine (0.41 g, 3.84 mmol) were used; and the reactions were conducted under ice bath and naturally elevated to at room temperature. [b] Separated by column chromatography. [c] The loading of **2a** was 30 g.

We systematically investigated the reaction conditions under various reaction conditions. In this process, TCCA acted as a powerful dehydrating agent that could effectively accelerate the reaction. While processed with ethyl acetoacetate and benzylamine using TCCA as a catalyst, the condensation reaction was completed within 10~60 min, which greatly shorten the reaction time (Table S1, entries 3-12). However, the conventional reaction conditions, which were directly condensed or catalyzed by acetic acid, required a long reaction time and even experienced an insufficient conversion (entries 1 and 2). Screening various solvents such as dichloromethane, ethyl acetate, tetrahydrofuran, acetonitrile as reaction reagents, and acetonitrile was proved to be the best in terms of output (entries 3-7). It was noted that when tetrahydrofuran was used as a solvent, the reaction was a severely exothermic process, leading to black sticky substance. Subsequently, efforts to optimize the quantity of TCCA at room temperature in acetonitrile were successful (Table 1, entries 7-11). It was found that the reaction can be quickly completed in 10 minutes with only 2 mol% TCCA, and the corresponding β-enediamide ester (95%) was obtained almost quantitatively (entry 10). Interestingly, increasing or decreasing the amount of TCCA failed to further improve the conversion. In contrast, the

reaction was incomplete with a low yield (57-82%) with less than 2 mol% of TCCA, even if the time was prolonged to 1 h (entries 7-9). Finally, to demonstrate the scalability of our method, the reaction was carried out at 230 mmol in 15 minutes, providing 47 g of 3a in 47% yield (entry 12).

Spectrum of β-aminopropenones and β-aminopropionates

Spectrum of 3a

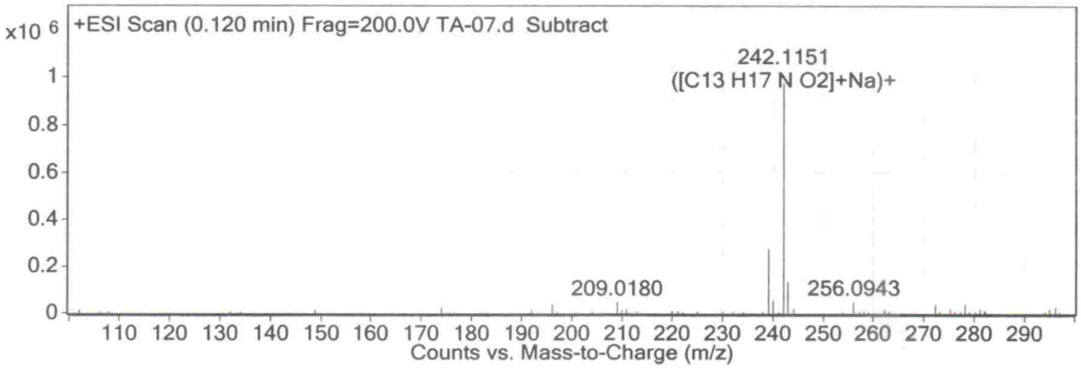

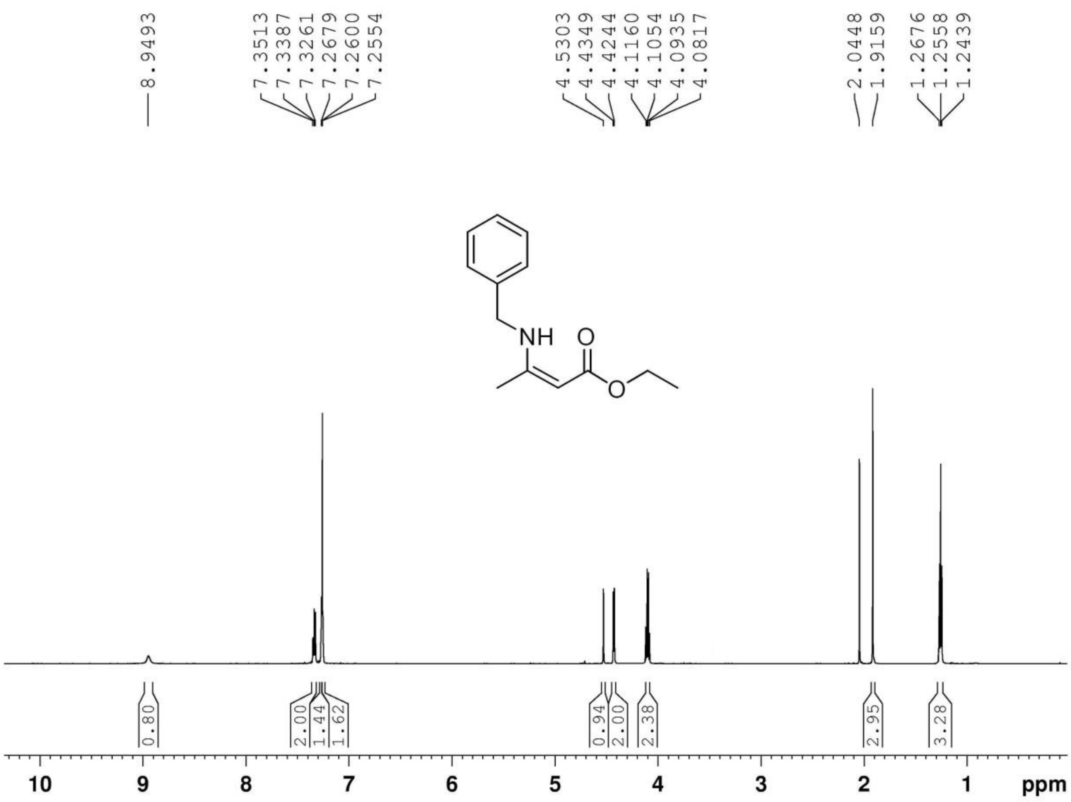

Spectrum of 3b

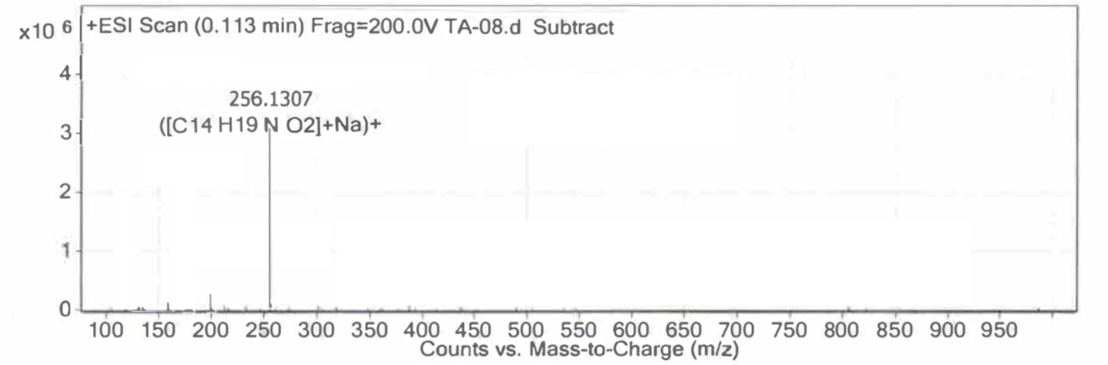

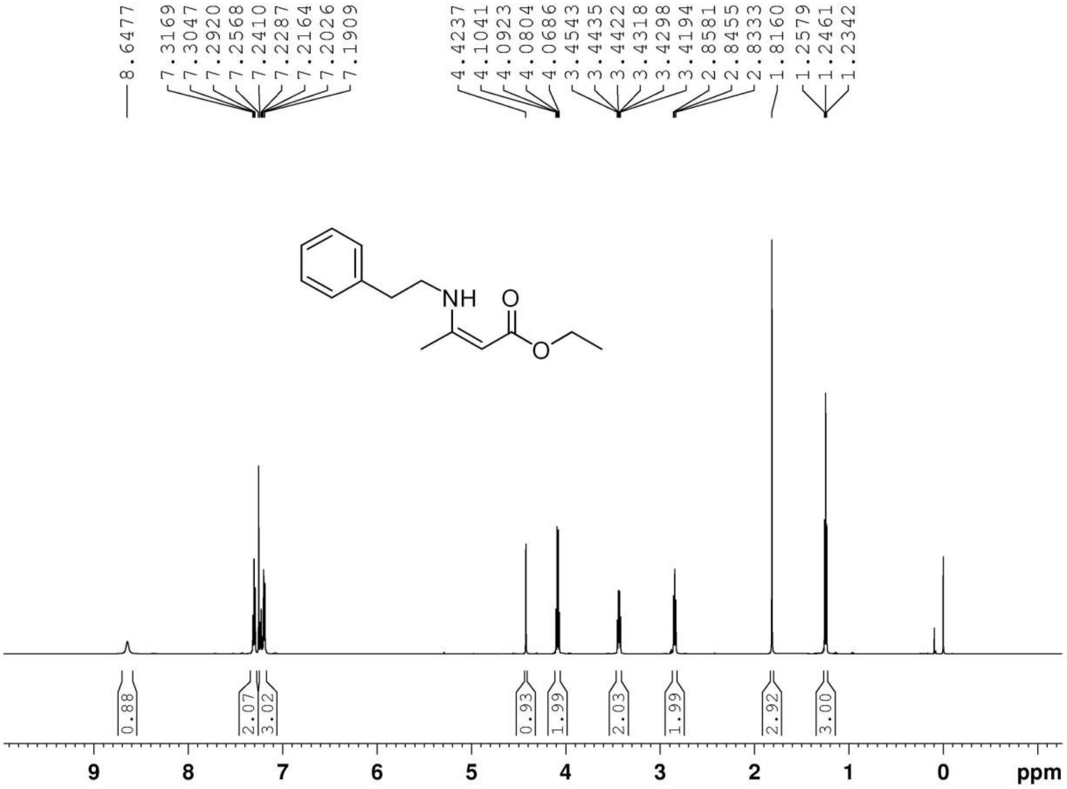

Compound 3c

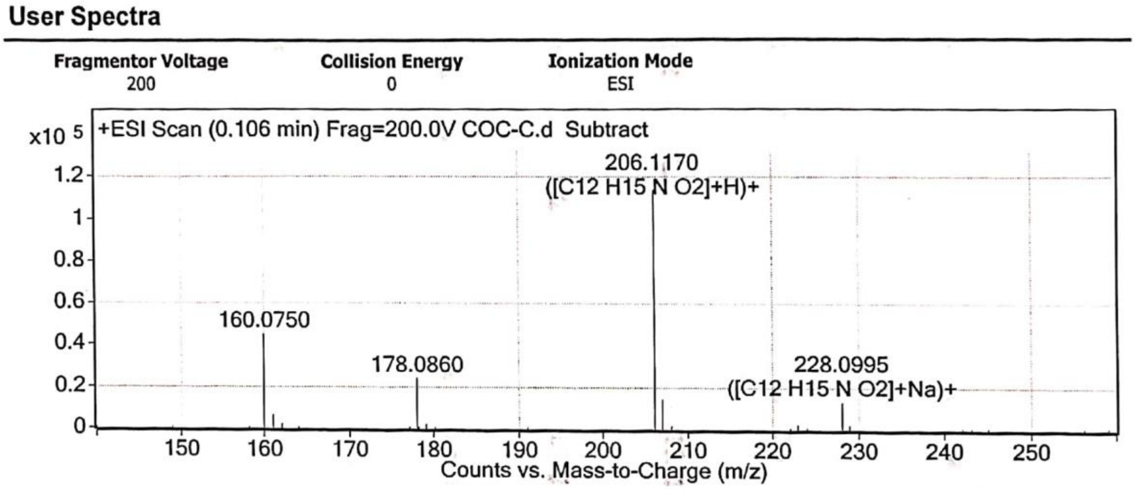
User Spectra
Fragmentor Voltage
200
Collision Energy
0
Ionization Mode
ESI
x10 5
+ESI Scan (0.106 min) Frag=200.0V COC-C.d Subtract
206.1170
([C12 H15 N O2]+H)+
160.0750
178.0860
228.0995
([C12 H15 N O2]+Na)+
Counts vs. Mass-to-Charge (m/z)

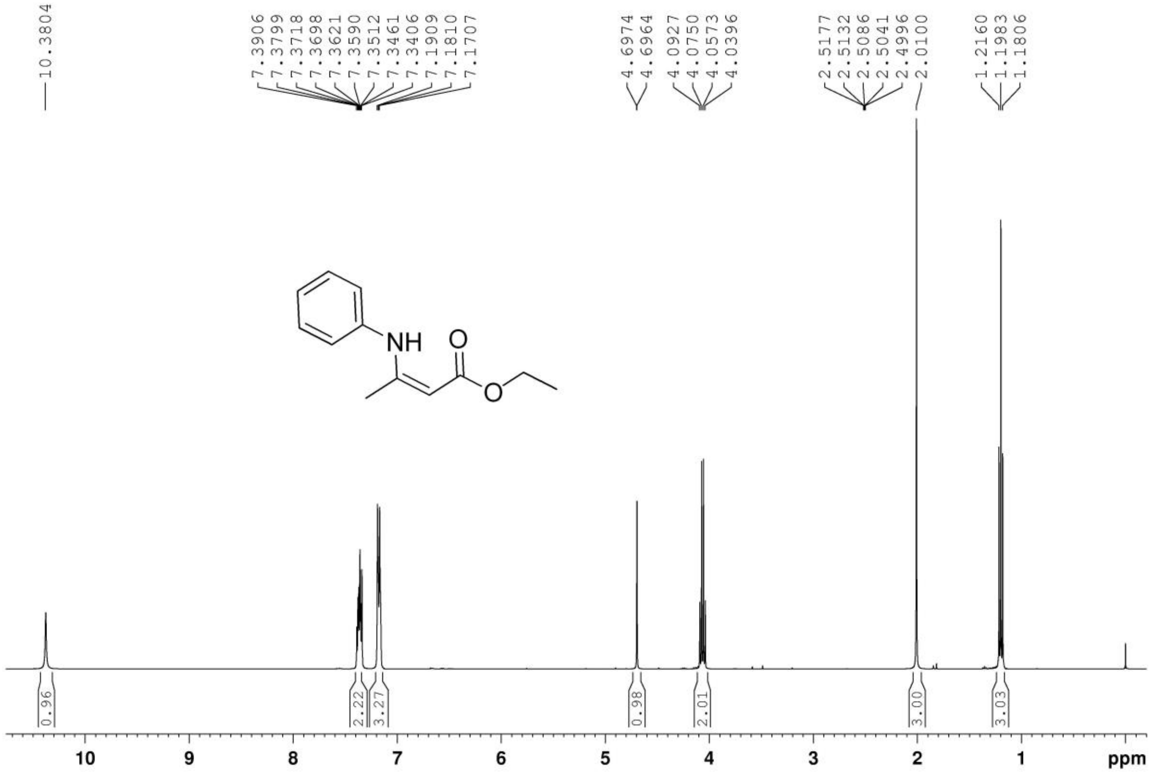
10.3804
7.3906
7.3799
7.3718
7.3698
7.3621
7.3590
7.3512
7.3461
7.3406
7.1909
7.1810
7.1707
4.6974
4.6964
4.0927
4.0750
4.0573
4.0396
2.5177
2.5132
2.5086
2.5041
2.4996
2.0100
1.2160
1.1983
1.1806
NH
O
O
0.96
2.22
3.27
0.98
2.01
3.00
3.03
ppm

Compound 3d

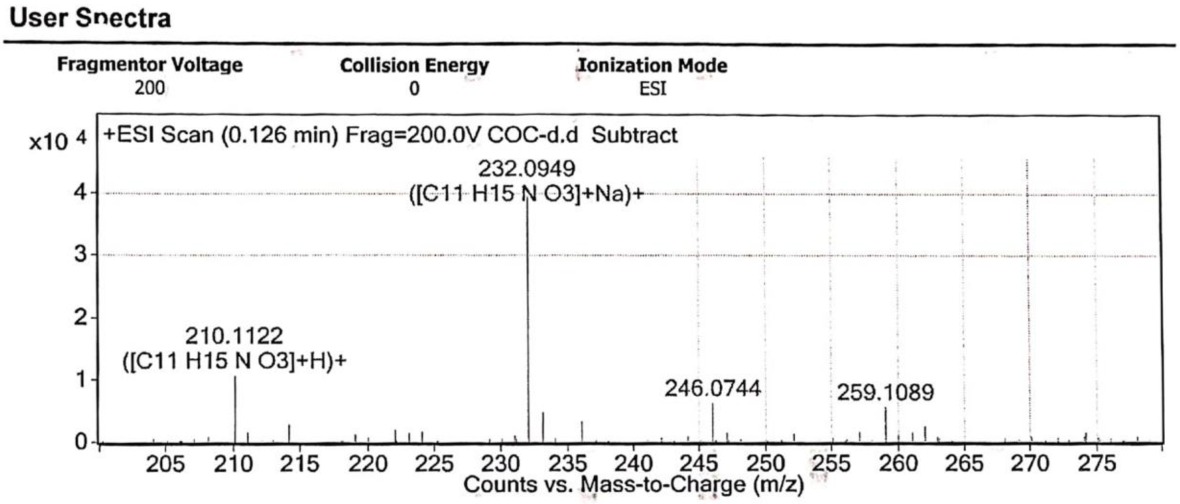
User Spectra
Fragmentor Voltage
200
Collision Energy
0
Ionization Mode
ESI
x10 4
+ESI Scan (0.126 min) Frag=200.0V COC-d.d Subtract
232.0949
([C11 H15 N O3]+Na)+
210.1122
([C11 H15 N O3]+H)+
246.0744
259.1089
Counts vs. Mass-to-Charge (m/z)

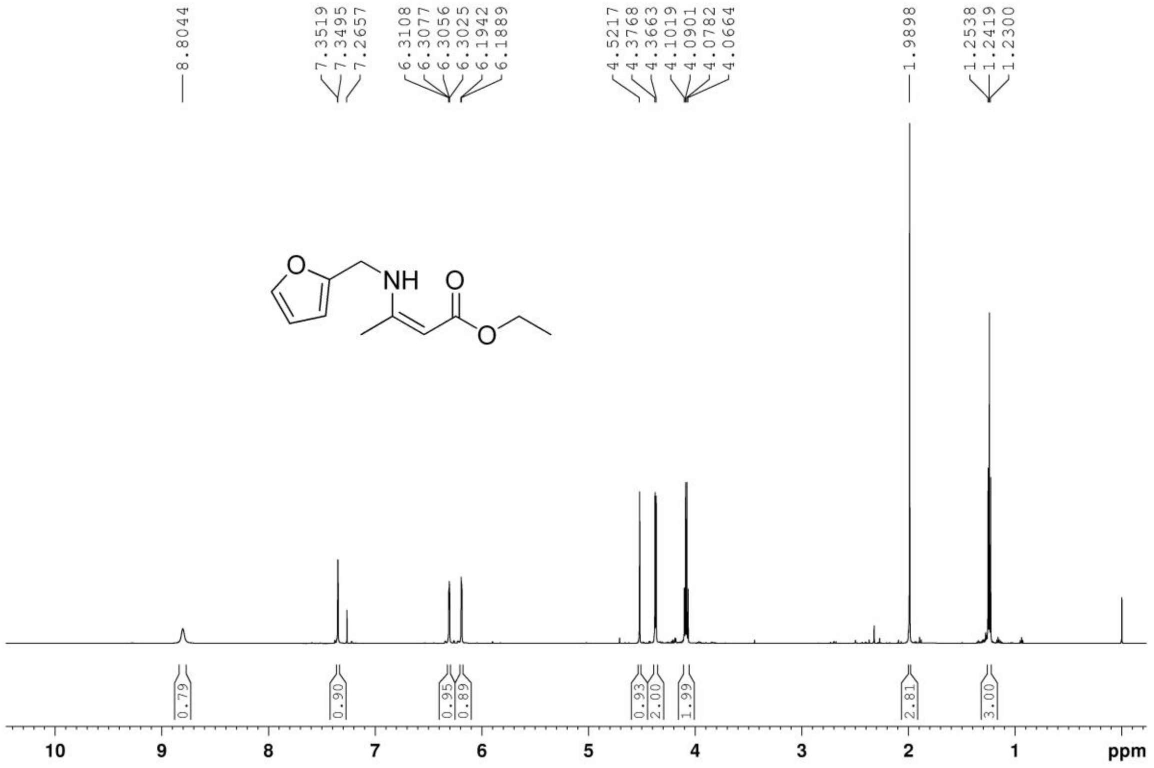
8.8044
7.3519
7.3495
7.2657
6.3108
6.3077
6.3056
6.3025
6.1942
6.1889
4.5217
4.3768
4.3663
4.1019
4.0901
4.0782
4.0664
1.9898
1.2538
1.2419
1.2300
O
NH
O
O
0.79
0.90
0.95
0.89
0.93
2.00
1.99
2.81
3.00
ppm

Compound 3e

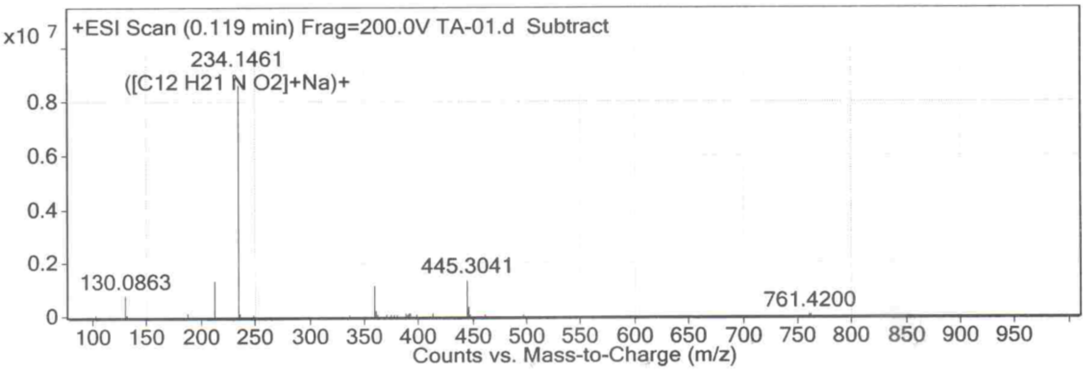

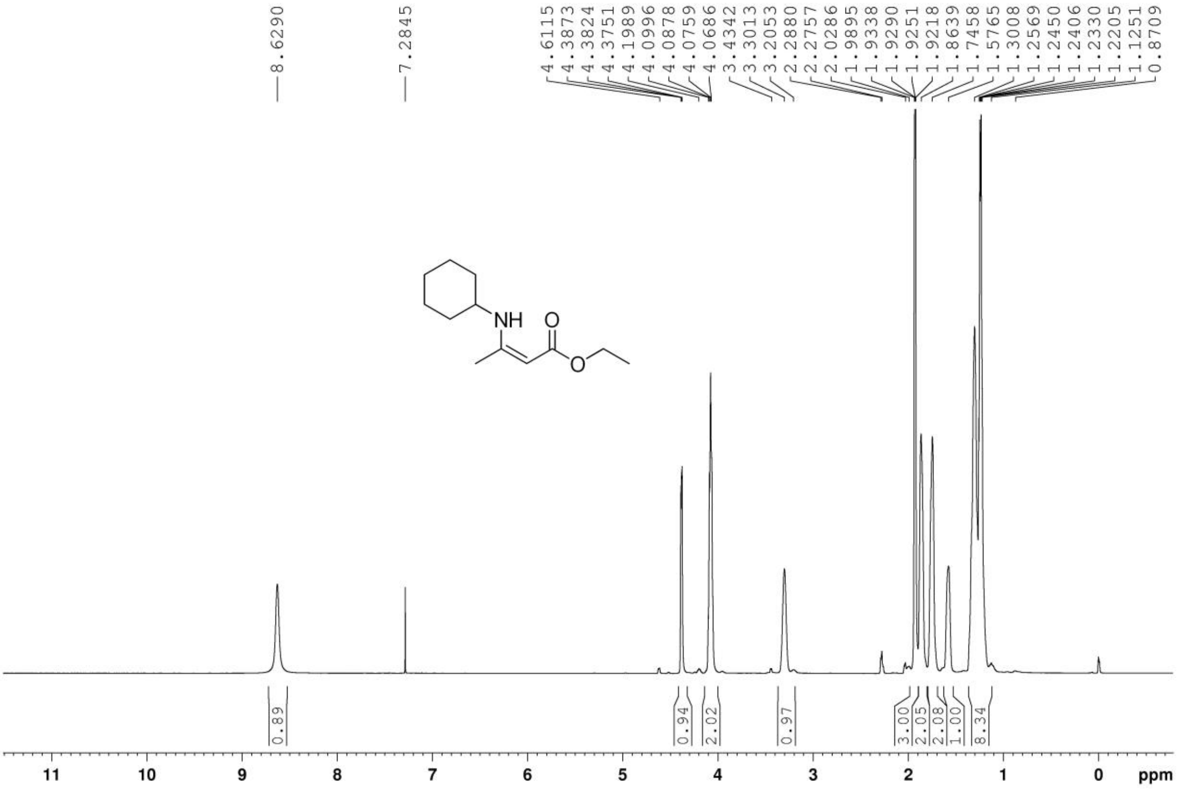

Spectrum of 3f

User Spectra

Fragmentor Voltage	Collision Energy	Ionization Mode
200	0	ESI

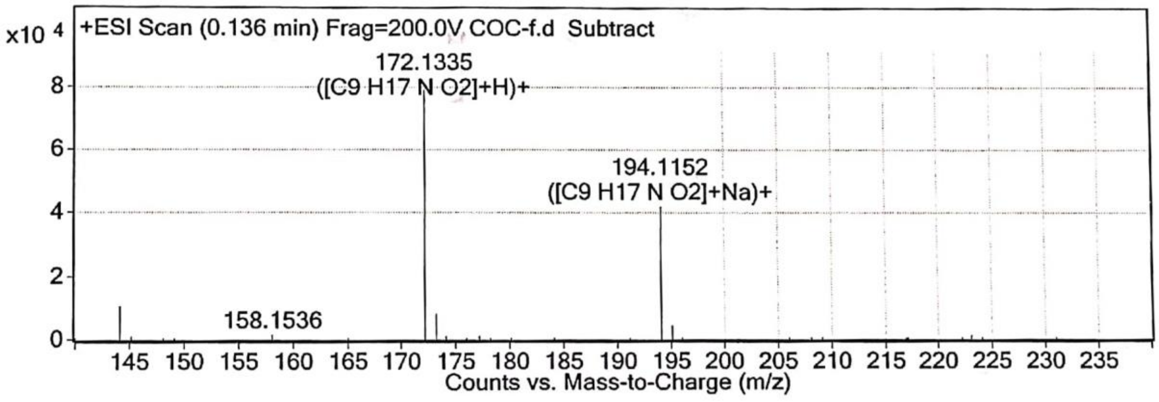

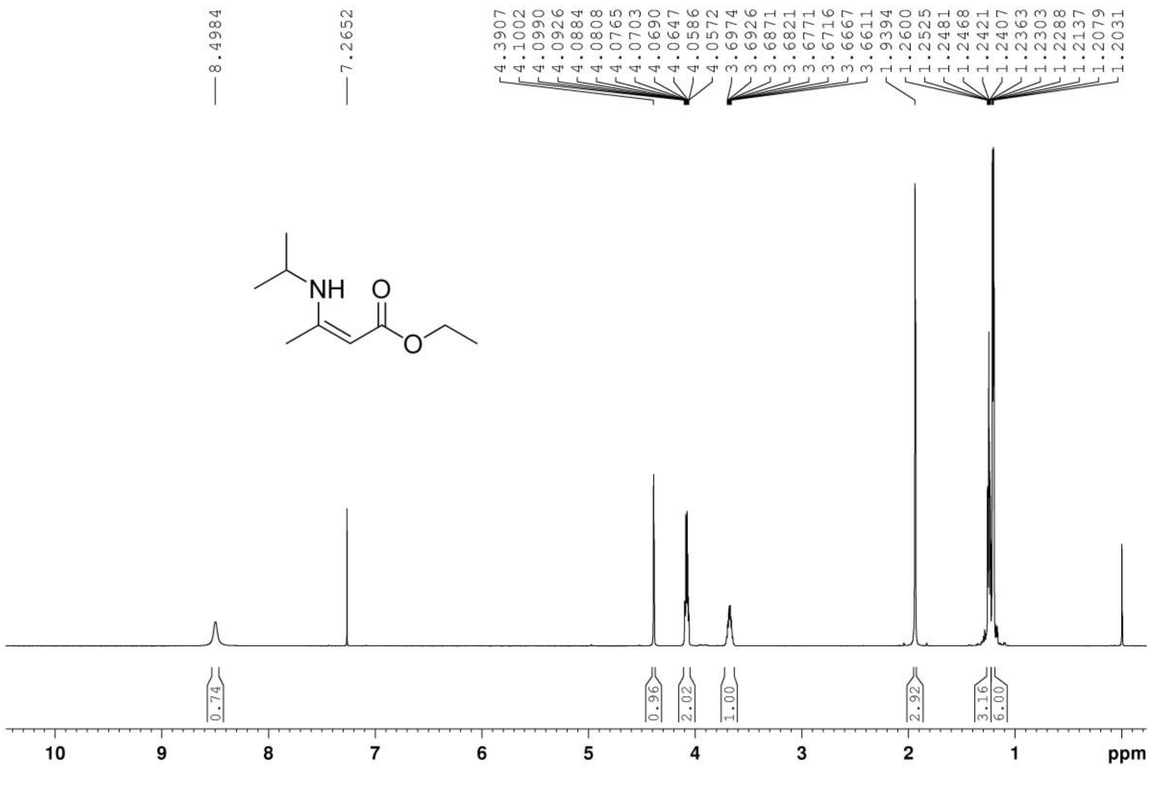

Spectrum of 3g

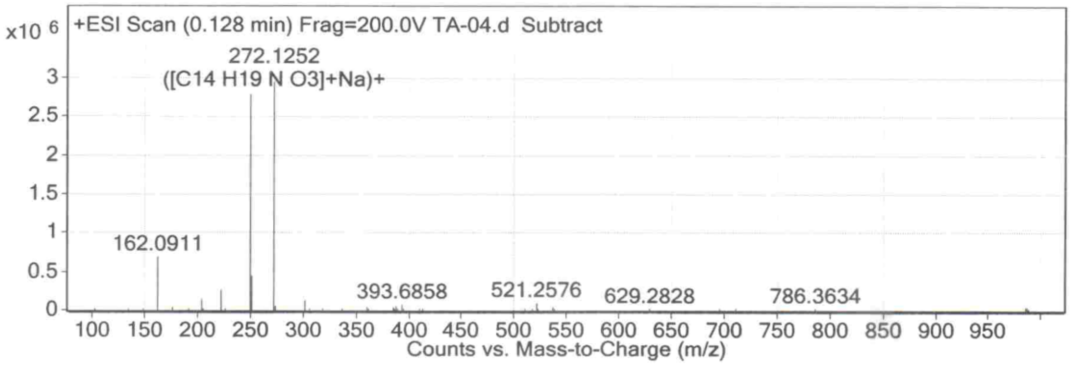

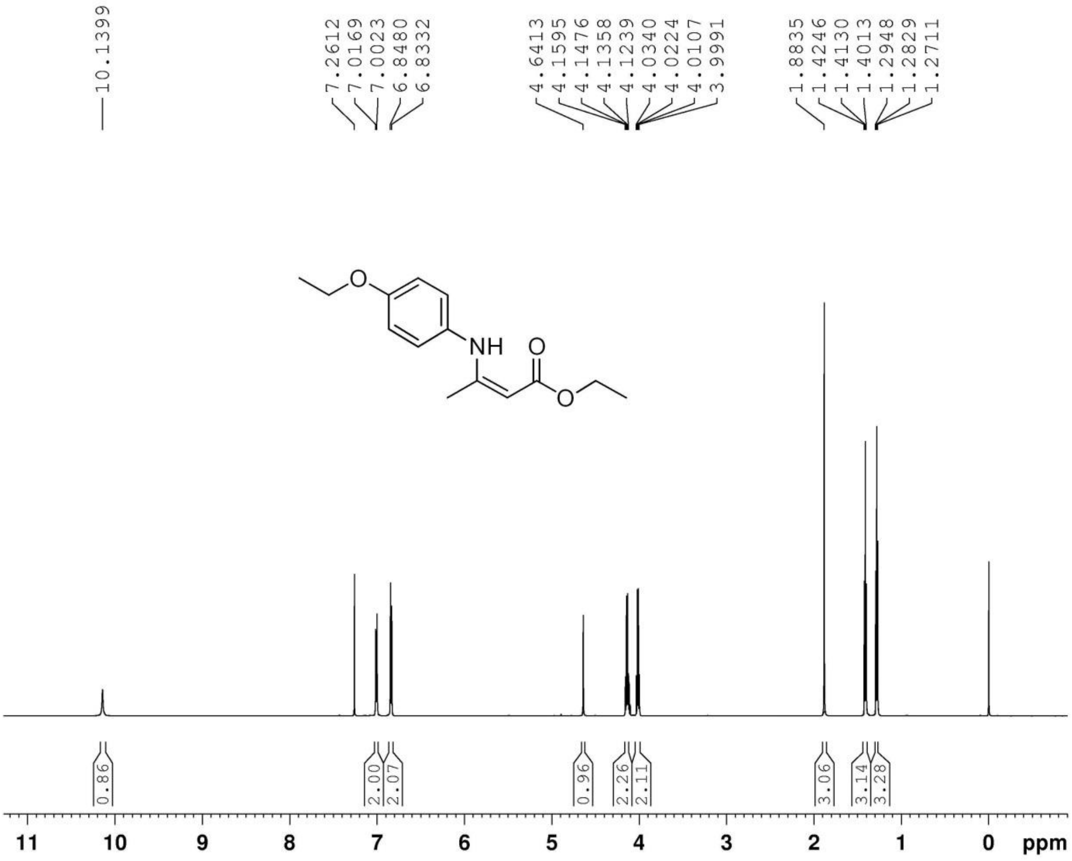

Spectrum of 3h

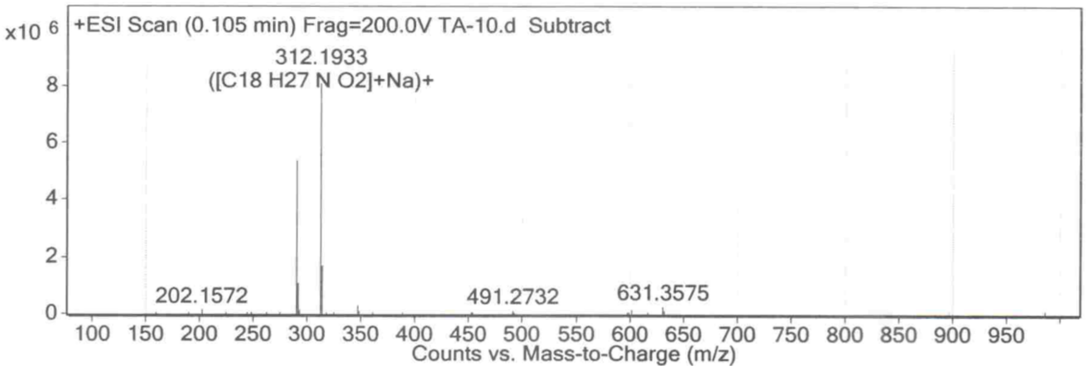

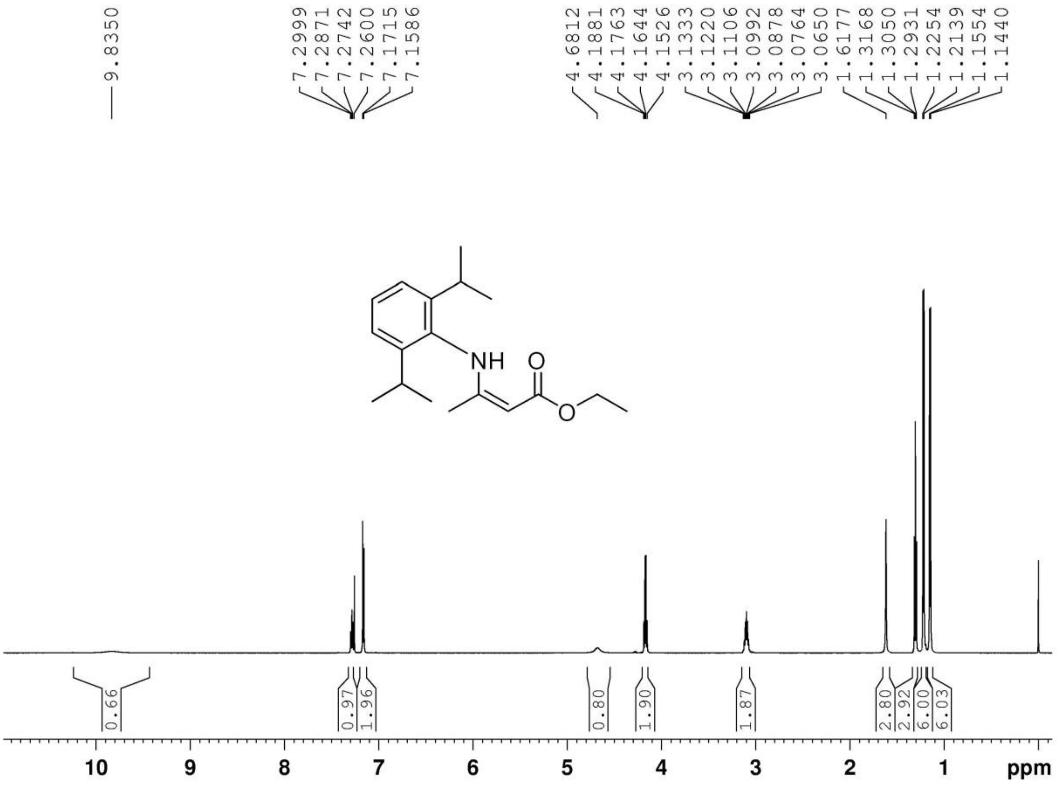

Spectrum of 3i

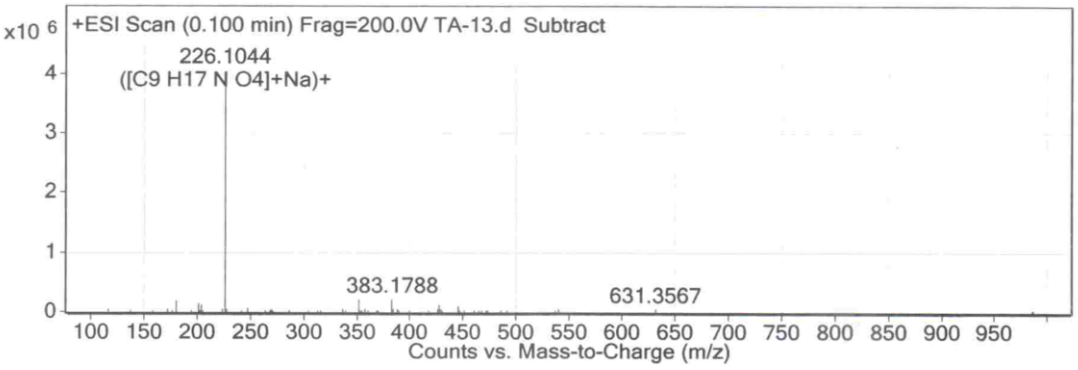

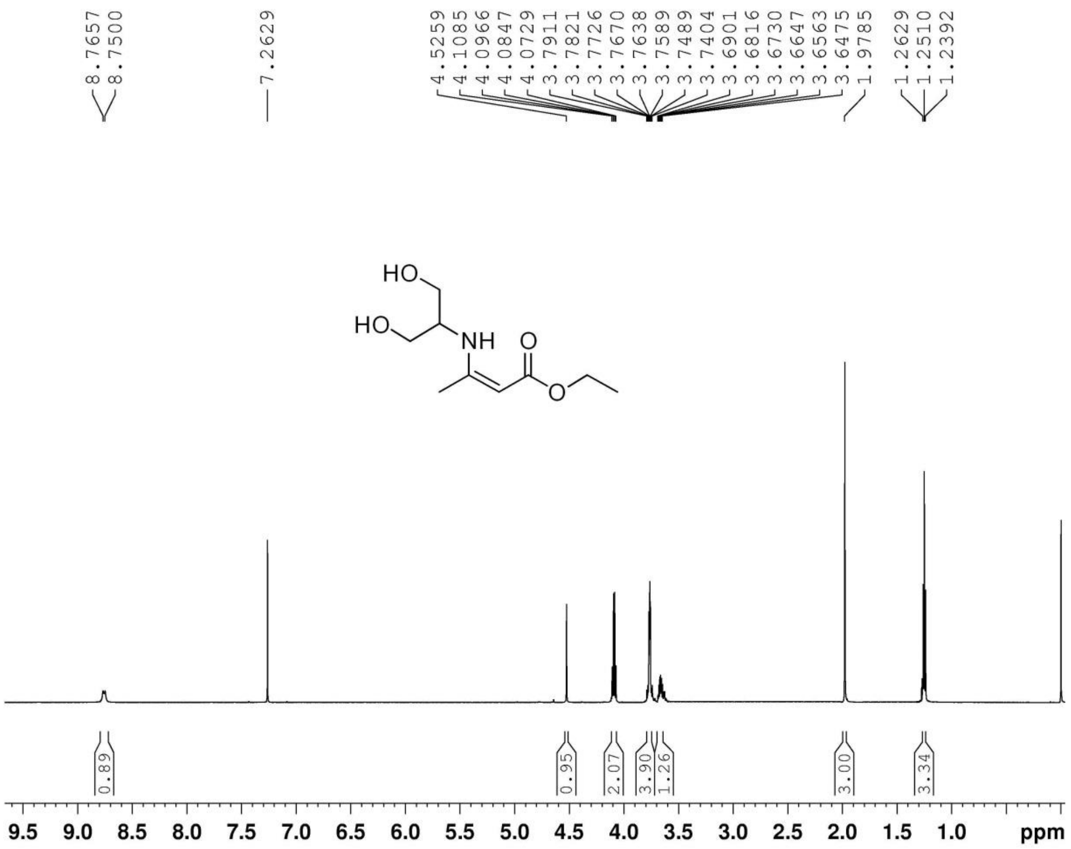

Spectrum of 3j

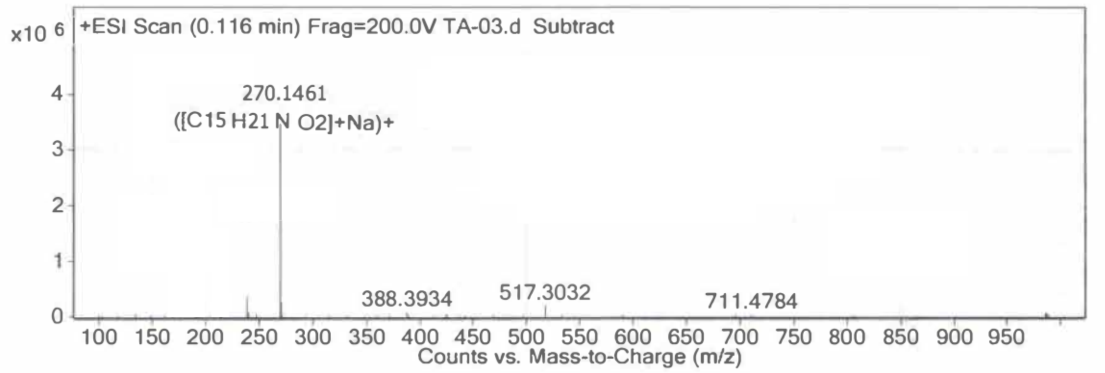

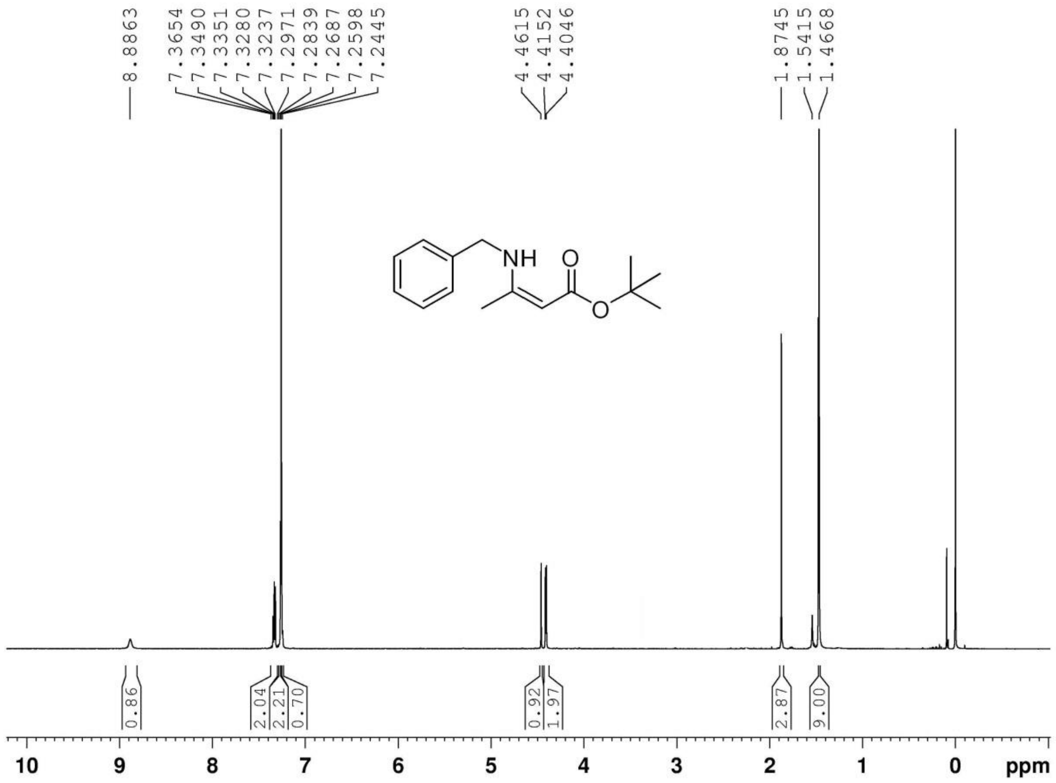

Spectrum of 3k

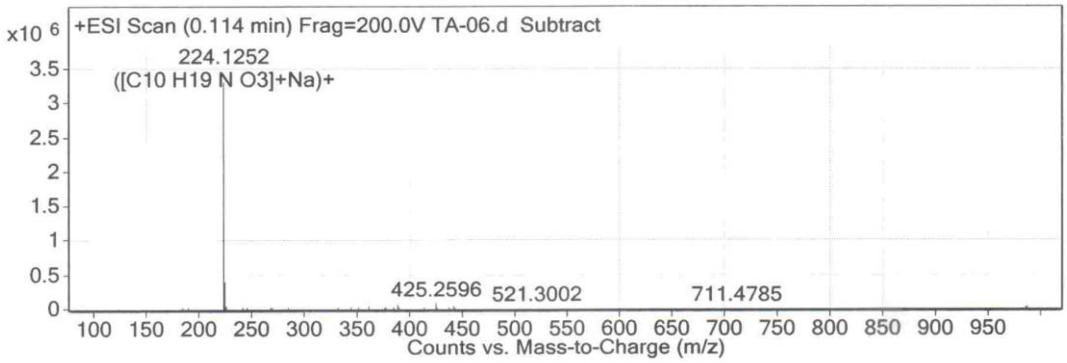

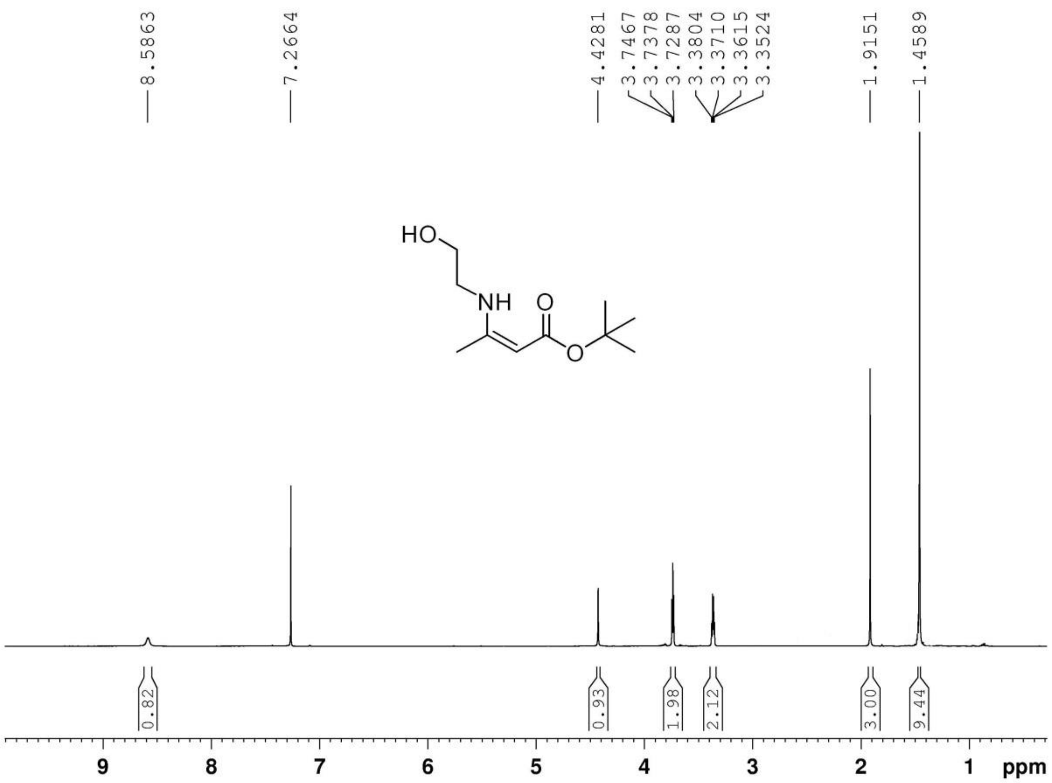

Spectrum of 3l

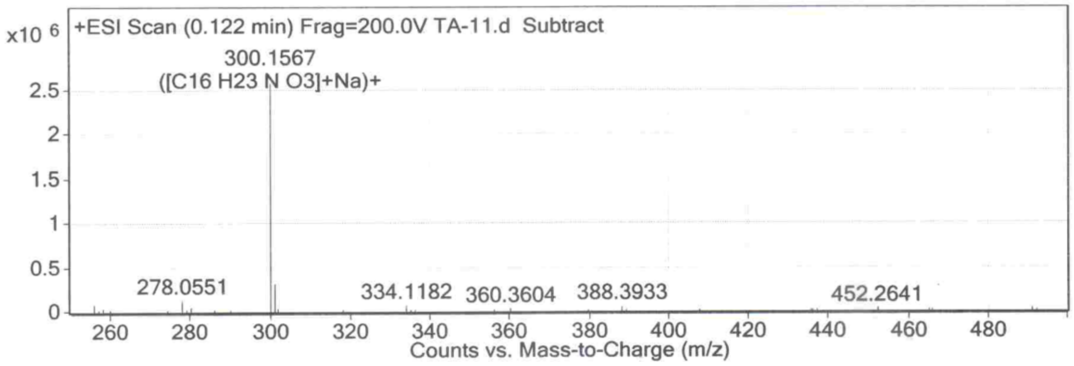

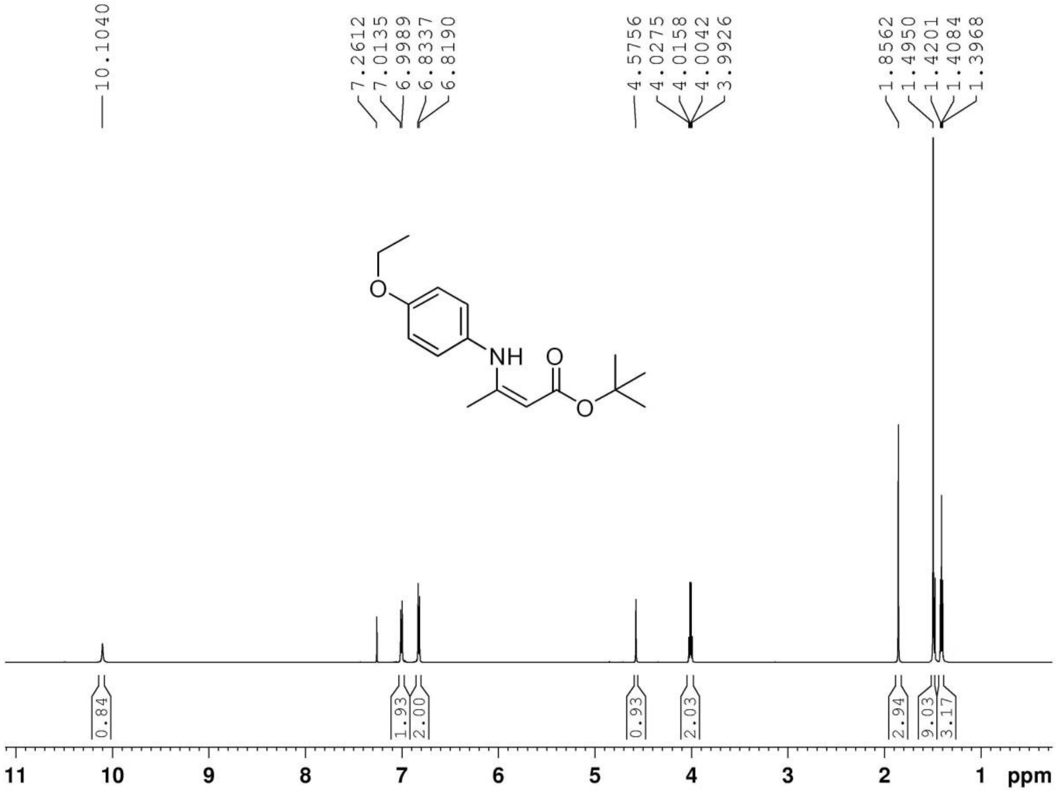

Spectrum of 3m

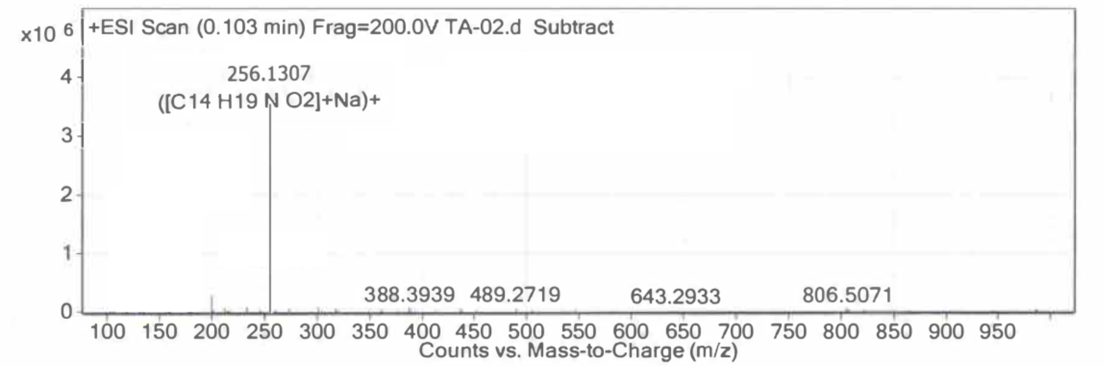

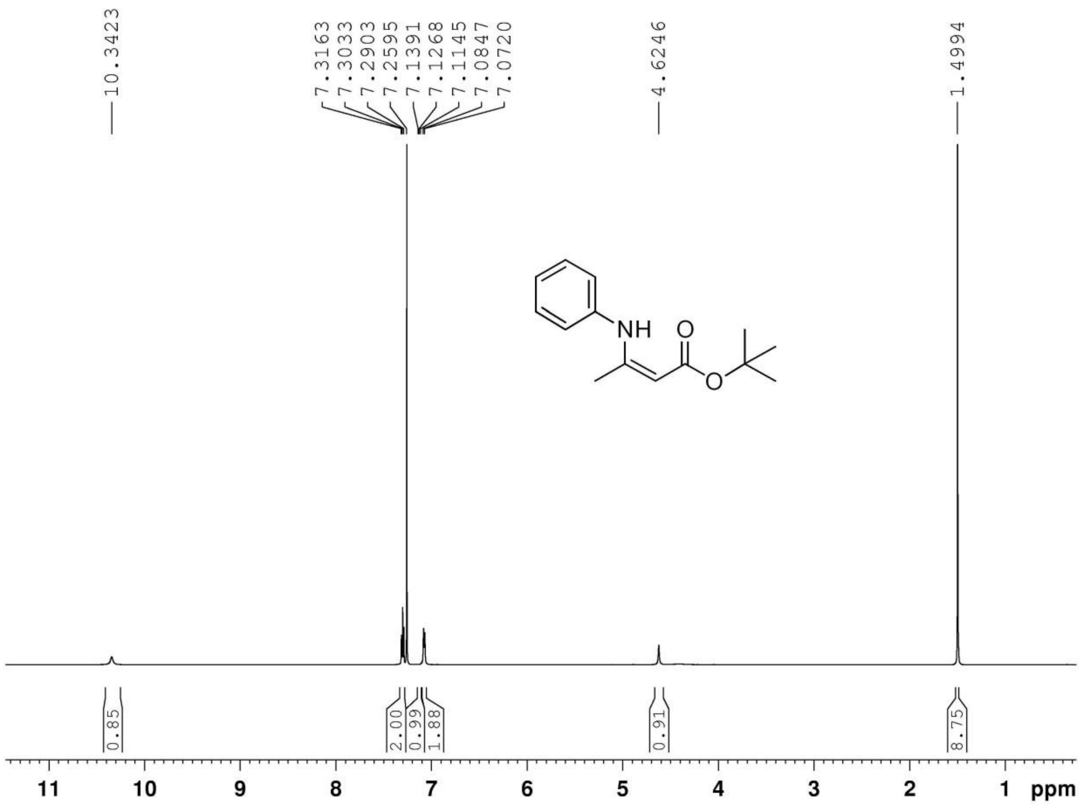

spectrum of 3n

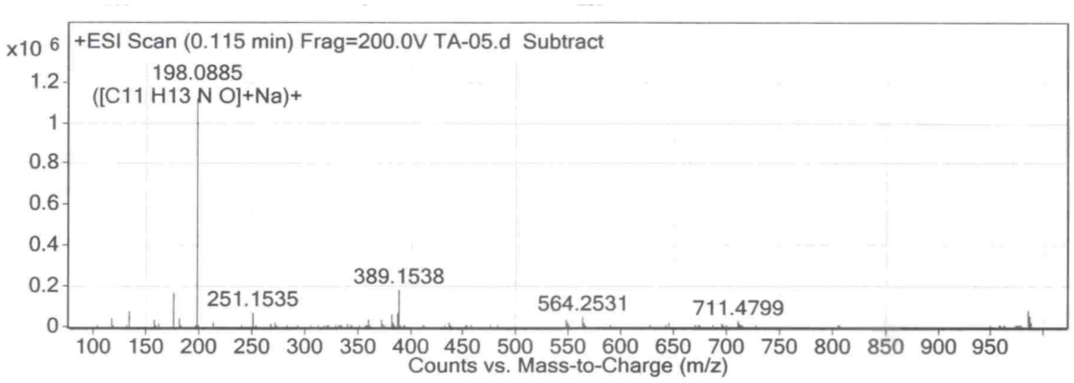

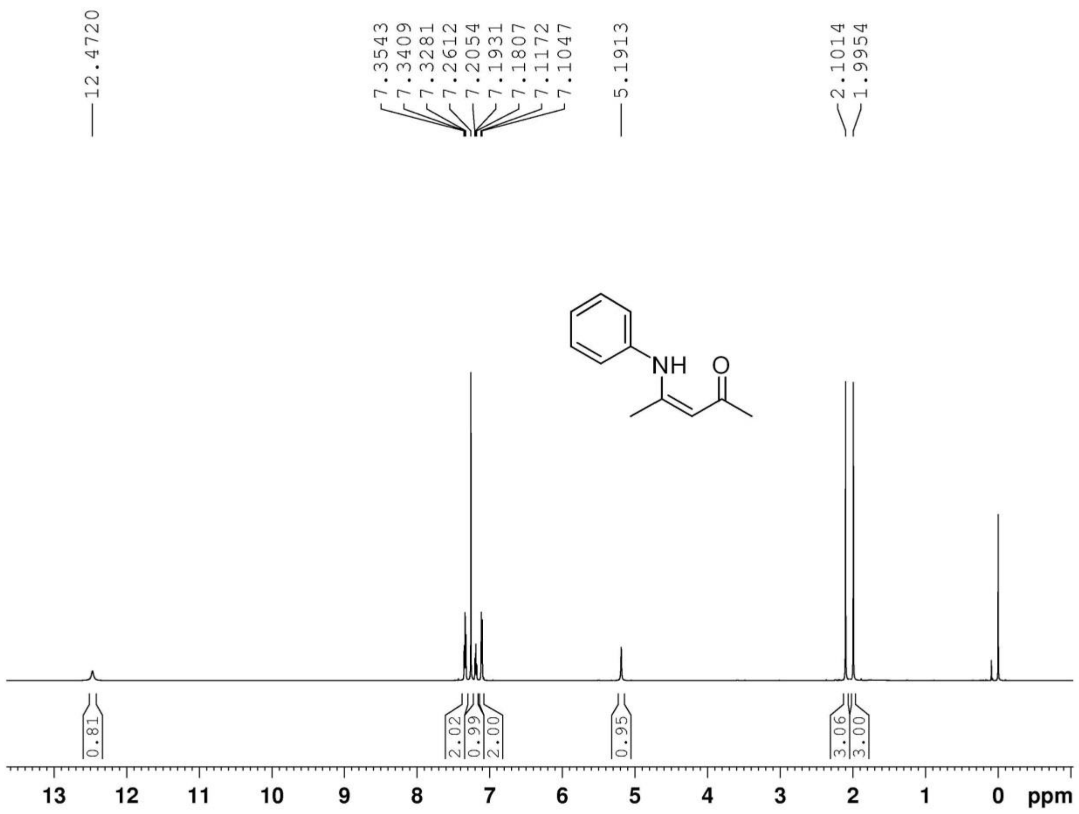

Spectrum of 3o

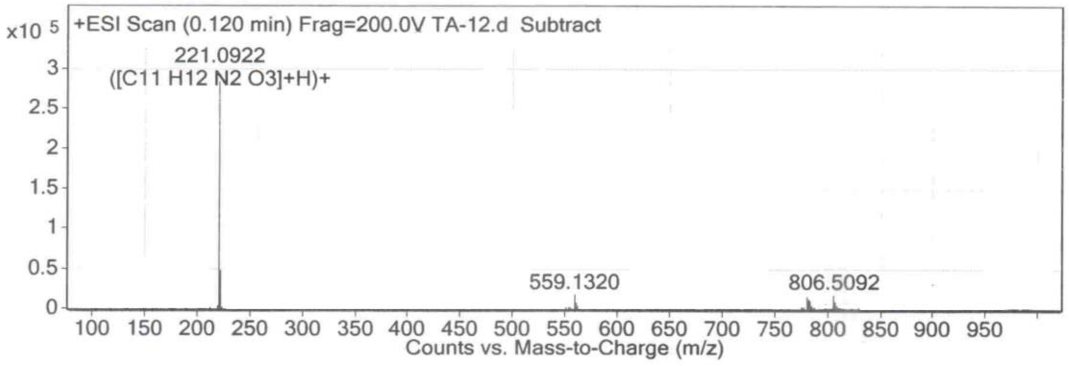

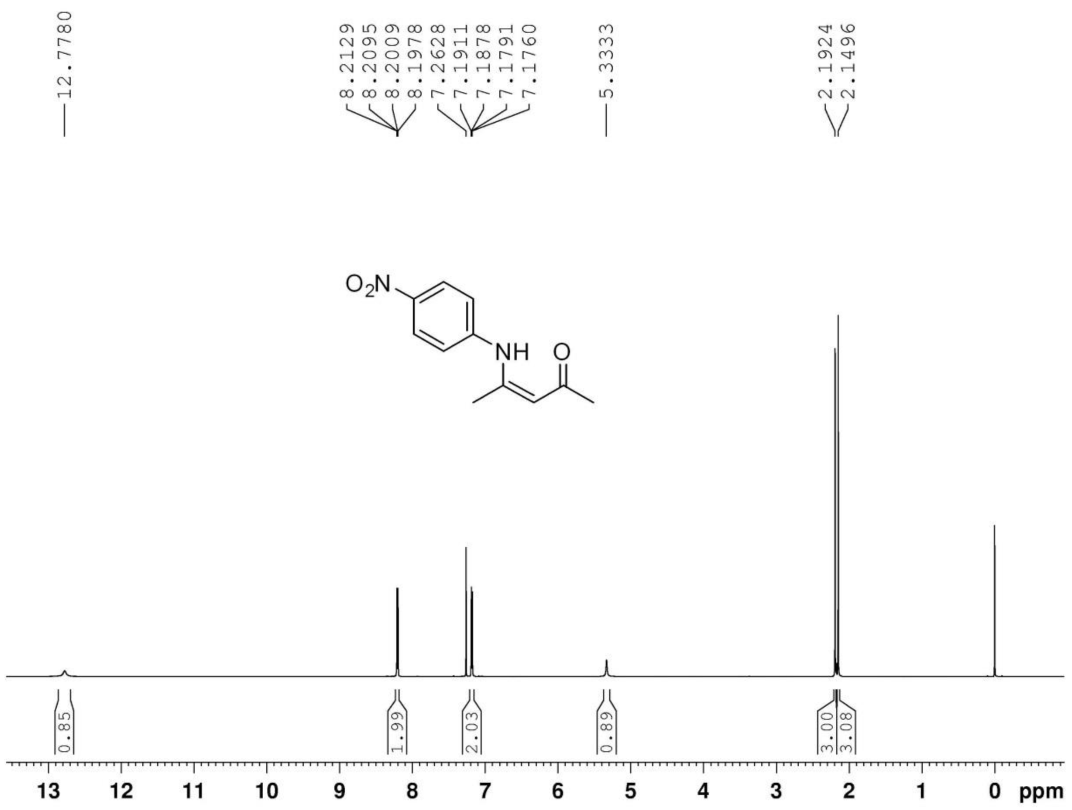

Spectrum of 3p

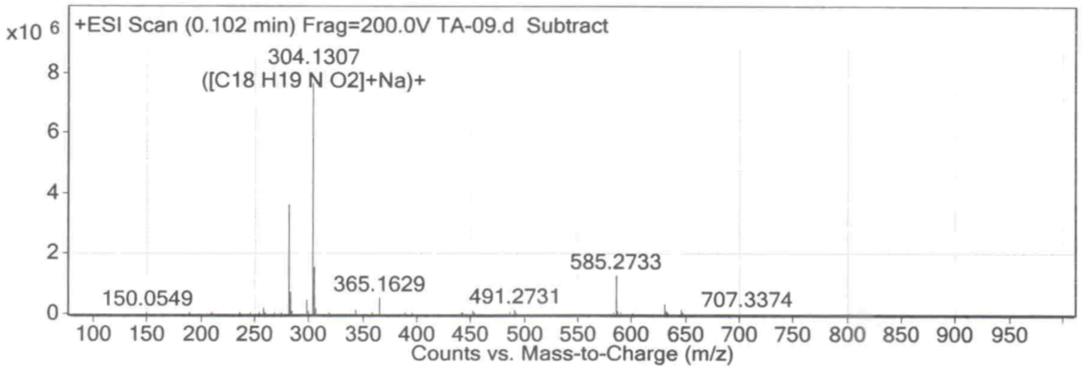

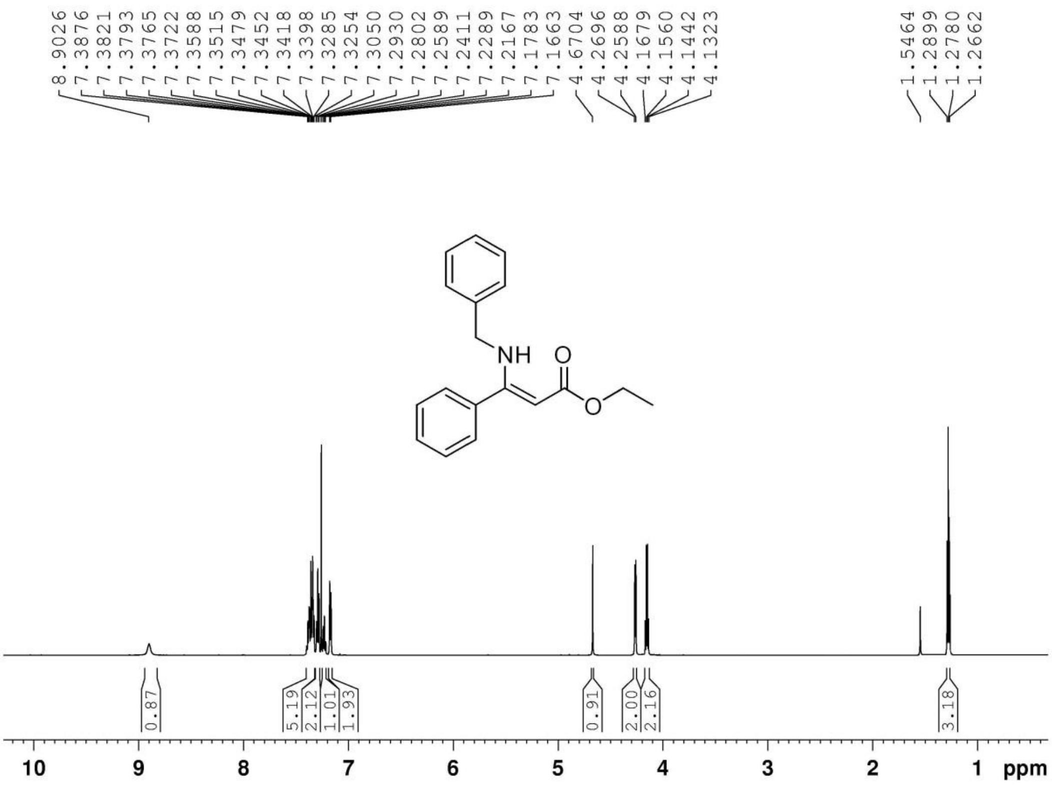

Preparation of pyridone derivatives

General procedure for α,β-unsaturated ketone (B4a-b, B5a-c)

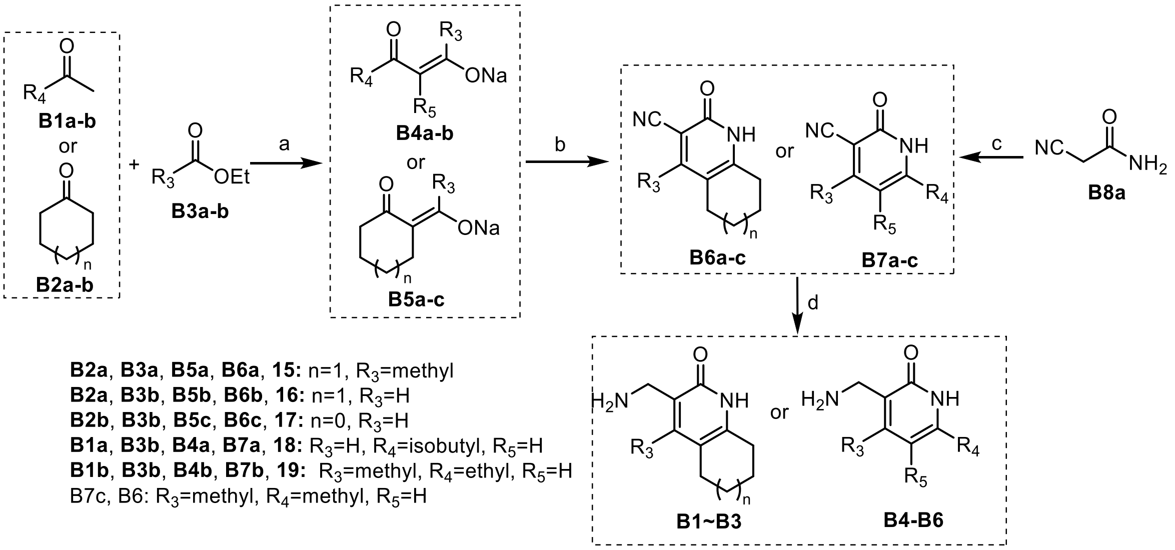

Reactions and conditions: (a) Metal sodium, Et_2O, 0~20 ^oC, 6 h; (b) 1) Cyanoacetamide, piperidine acetate, r.t.~100 ^oC, 2 h; 2) 10 N HCl; (c) Acetylacetone, K_2CO_3, H_2O, r.t., 24 h; (d) H_2, Ni, MeOH, 25% aq. NH_4OH, 60 ^oC, 11 h.

Sodium 1-(2-oxocyclohexylidene)ethan-1-olate (B5a)

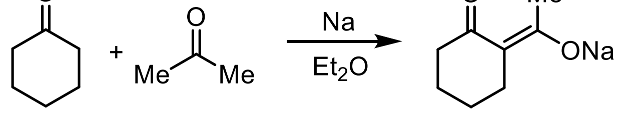

To a solution of absolute ether (300 mL) was added metal sodium (3.2 g, 0.14 mol) in portions at ice bath. Then, the mixture of 4-methyl-2-pentanone (14.0 g, 0.14 mol) and ethyl formate (10.3 g, 0.14 mol) was added dropwise in 1 h and stirred at ice bath for 5 h to allow solid precipitation. The solid was filtered and washed with ether to give a white solid (18 g, 79%). It was used in the following reaction without any further purification.

Sodium (E)-5-methyl-3-oxohex-1-en-1-olate (B4a). The titled product was obtained from cyclohexanone and ethyl acetate and according to the general procedure described previously for the α,β-unsaturated ketone as a yellow oily solid (16 g, 76%). It was used in the following reaction without any further purification.

Sodium (*E*)-4-oxohex-2-en-2-olate (B4b). The titled product was obtained from butanone and ethyl acetate and according to the general procedure described previously for the α,β-unsaturated ketone as a yellow oily solid (14 g, 74%). It was used in the following reaction without any further purification.

Sodium (*E*)-(2-oxocyclohexylidene)methanolate (B5b). The titled product was obtained from cyclohexanone and ethyl formate and according to the general procedure described previously for the α,β-unsaturated ketone as a light yellow solid (19 g, 92%). It was used in the following reaction without any further purification.

Sodium (*E*)-(2-oxocyclopentylidene)methanolate (B5c). The titled product was obtained from cyclopentanone and ethyl formate and according to the general procedure described previously for the α,β-unsaturated ketone as a yellow solid (18 g, 96%). It was used in the following reaction without any further purification.

General procedure for 3-cyanopyridone derivatives (B6a-c, B7a-b)

4-Methyl-2-oxo-1,2,5,6,7,8-hexahydroquinoline-3-carbonitrile (B7a)

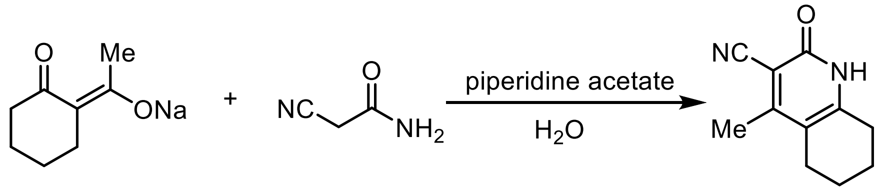

To a solution of **B4a** (13.5 g, 0.09 mol), 2-cyanoacetamide (8.2 g, 0.097 mol) in water were added piperidinium acetate (6 mL) and the mixture was heated to 100 °C for 2 h. After cooling to 0 °C, the pH was adjusted to pH 4◎5 using 10 N aq. HCl. The resulting precipitated was filtered off, washed with water, ether and dried to provide brown solid (9.0 g). The crude product was recrystallized from methanol to obtain a yellow solid (7.5 g, 36%) exhibiting the following spectroscopic characteristics. ^{1}H NMR (600 MHz, DMSO-*d*$_6$) δ (ppm): 12.52 (br, 1H), 8.04 (d, *J*=7.3 Hz, 1H), 6.21 (d, *J*=7.4 Hz, 1H), 2.41 (d, *J*=7.4 Hz, 2H), 1.98-1.91 (m, 1H), 0.87 (d, *J*=6.6 Hz, 6H).

6-Ethyl-4-methyl-2-oxo-1,2-dihydropyridine-3-carbonitrile (**B7b**). The titled product was obtained from **B4b** and 2-cyanoacetamide and according to the general procedure described previously for the 3-cyanopyridone derivatives as a grayish white solid (13.0 g, 24%). ^{1}H NMR (600 MHz, DMSO-*d*$_6$) δ (ppm): 12.27 (br, 1H), 6.19 (s, 1H), 2.52-2.48 (q, 4H), 2.32 (s, 3H), 1.15 (t, *J*=7.6 Hz, 3H).

4-Methyl-2-oxo-1,2,5,6,7,8-hexahydroquinoline-3-carbonitrile (**B6a**). The titled product

was obtained from **B5a** and 2-cyanoacetamide and according to the general procedure described previously for the 3-cyanopyridone derivatives as a white solid (20.0 g, 31%). ^{1}H NMR (600 MHz, DMSO-*d*$_6$) δ (ppm): 12.32 (br, 1H), 2.72 (t, *J*=5.9 Hz, 2H), 2.38 (t, *J*=6.3 Hz, 2H), 2.21 (s, 3H), 1.68-1.66 (m, 4H).

2-Oxo-1,2,5,6,7,8-hexahydroquinoline-3-carbonitrile (**B6b**). The titled product was obtained from **B5b** and 2-cyanoacetamide and according to the general procedure described previously for the 3-cyanopyridone derivatives as a yellow solid (7.0 g, 25%). ^{1}H NMR (600 MHz, DMSO-*d*$_6$) δ (ppm): 11.64 (br, 1H), 7.89 (s, 1H), 2.56 (t, *J*=6.1 Hz, 2H), 2.42 (t, *J*=6.2 Hz, 2H), 1.70-1.60 (m, 4H).

2-Oxo-2,5,6,7-tetrahydro-1*H*-cyclopenta[*b*]pyridine-3-carbonitrile (**B6c**). The titled product was obtained from **B5c** and 2-cyanoacetamide and according to the general procedure described previously for the 3-cyanopyridone derivatives as a yellow solid (7.2 g, 21%). ^{1}H NMR (600 MHz, DMSO-*d*$_6$) δ (ppm): 12.75 (br, 1H), 8.01 (s, 1H), 2.80 (t, *J*=7.6 Hz, 2H), 2.64 (t, *J*=7.3 Hz, 2H), 2.06-2.01 (m, 2H).

4,6-Dimethyl-2-oxo-1,2-dihydropyridine-3-carbonitrile (B7c).

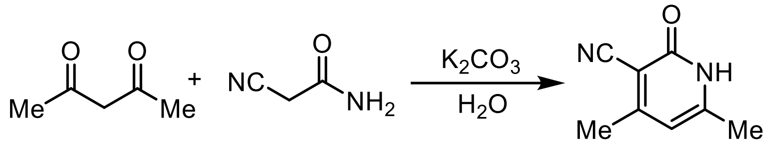

To a solution of potassium carbonate (13.8 g, 0.1 mol), acetylacetone (11.0 g, 0.1 mol) in water (100 mL), cyanoacetamide (8.4 g, 0.1 mol) were added in portions at room temperature. The mixture was stirred at room temperature, and a white solid precipitated after 60 min. The mixture was stirred for 24 h and then filtered, washed with water, dried to give crude product, whcih was recrystallized from menthol to obtain a colorless needle solid (13 g, 88%) exhibiting the following spectroscopic characteristics. M.p. 280-281 ^{o}C (lit: 286 ^{o}C). ESI-MS m/z: 149.1 [M+H]$^+$. ^{1}H NMR (600 MHz, DMSO-*d*$_6$): δ (ppm) 6.16 (s, 1H), 2.30 (s, 3H), 2.22 (s, 3H).

General procedure for reduction reaction of carbonitrile

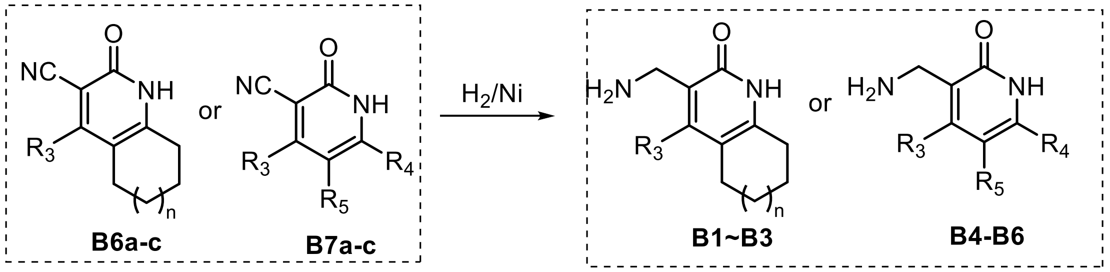

To a stirred mixture of carbonitrile (0.034 mol) and 25% ammonia (25 mL) in methanol (250 mL) was added Raniy Ni (5 g). The resulting suspension was evacuated and backfilled with argon three times. A hydrogen balloon was added and the flask was evacuated and backfilled three times with hydrogen. The resulting suspension was heated to 50 °C for 11 h. The mixture was then cooled to rt, vented, and the suspension was filtered through a pad of celite. The filtrate was concentrated *in vacuo* to afford amine.

3-(Aminomethyl)-4,6-dimethylpyridin-2(1H)-one hydrochloride salt (B6). The titled product was obtained from carbonitrile **B7c** according to the general procedure described previously for the reduction reaction of carbonitrile as a light yellow solid (6.3 g). The

crude product was dissolved with menthol in ice bath below 0 °C, the 4 N hydrogen chloride ethanol solution (20 mL) was added dropwise. The stirring was continued for 60 min, and the solid filtered off and washed with ethanol. After drying, hydrochloride salt was obtained as a white solid (5.0 g, 97%). ^{1}H NMR (600 MHz, DMSO-d_6) δ (ppm): 11.84 (br, 1H), 8.04 (s, 3H), 5.97 (s, 1H), 3.77 (s, 2H), 2.21 (s, 3H), 2.16 (s, 3H). Compounds **B1~B5** were obtained from different carbonitrile (**B6a-c, B7a-b**) to provide the corresponding products according to the general procedure described previously for the reduction reaction of carbonitrile.

3-(Aminomethyl)-4-methoxy-6-methylpyridin-2(1H)-one hydrogen chloride (B7)

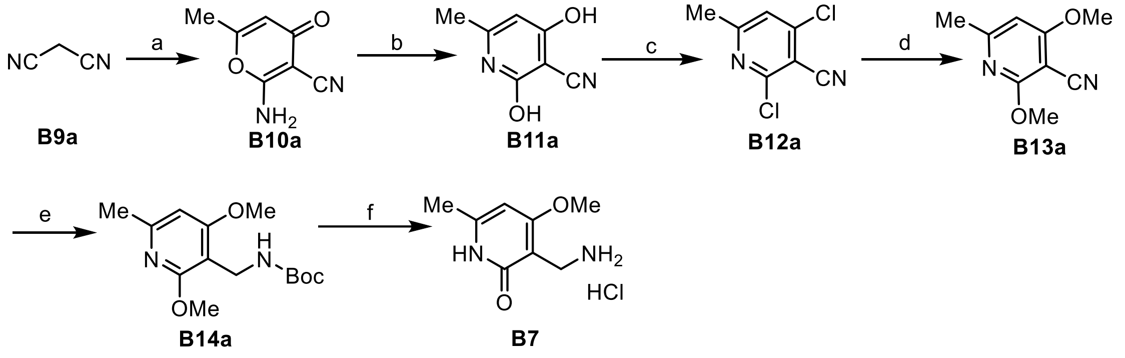

Reactions and conditions: (a) 1) 60% NaH, THF, 0 °C, 0.5 h; 2) 4-Methyleneoxetan-2-one, -10 °C, 1 h; (b) 4 N HCl, reflux, 5 h; (c) $POCl_3$, reflux, 3 h; (d) MeONa, MeOH, reflux, 4 h; (e) H_2, Raney Ni, Boc_2O, TEA, MeOH/THF, 30 °C, 48 h; (f) 4 N HCl, reflux, 5 h.

2-Amino-6-methyl-4-oxo-4H-pyran-3-carbonitrile (B10a).

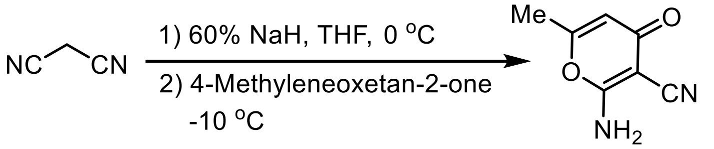

To a solution of malononitrile (10 g, 0.15 mol) in anhydrous THF (100 mL) were slowly added sodium hydride NaH (60% dispersion in mineral oil, 6.0 g, 0.15 mol) bellow 10 °C. The mixture was made to react for 30 min at 0 °C. After cooling to -10 °C, acetyl ketene (12.7 g, 0.15 mol) was added dropwise into solution, and the mixture was made to react for 1 h at -10 °C. After cooling to 0 °C, the pH was adjusted to pH 6-7 using 4 N HCl, and the solvent was evaporated under reduced pressure to afford orange viscous liquid (22.5 g). The crude was used in the following reaction without any further purification.

2,4-Dihydroxy-6-methylnicotinonitrile (B11a).

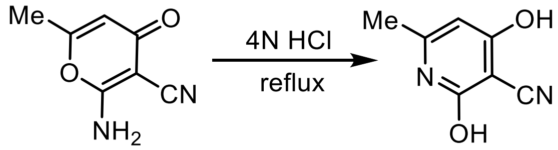

Carbonitrile **B10a** (22.5 g, 0.15 mol) was added to a stirred mixture of 4 N HCl (240 mL), and the reaction mixture was heated to reflux for 5 h. The mixture was cooled to r.t. and poured at 4 °C for 8 h to allow product precipitation. The solid was filtered and washed with water to give a yellow solid (9.8 g, 43%).

2,4-Dichloro-6-methylnicotinonitrile (B12a).

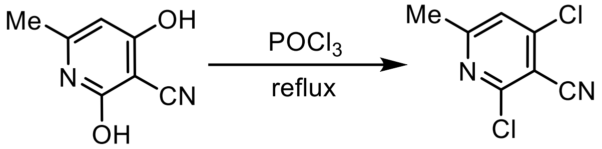

B11a (10 g, 66.6 mmol) was added to a stirred solution of DMF (1 mL) in $POCl_3$ (100 mL), and the mixture was heated to 70 °C for 3 h. The solvent $POCl_3$ was removed *via* vacuum distillation at 52 °C. The residue was dissolved in ethyl acetate, and the pH was adjusted to pH 7 using saturated sodium carbonate solution. The reaction mixture was filtered through a pad of celite, and the filtrate was extracted with ethyl acetate (40 mL×3), and then washed with water and brine. The organic solution was dried with anhydrous magnesium sulfate and evaporated under reduced pressure to afford a brown solid (10 g, 79%).

2,4-Dimethoxy-6-methylnicotinonitrile (B13a).

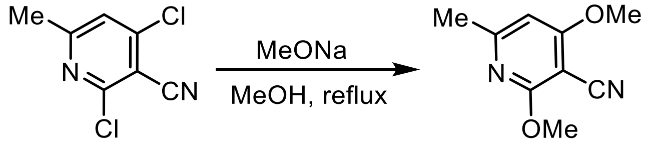

To a solution of **B12a** (9.0 g, 48 mmol) in methanol (60 mL) were added sodium methanolate (7 g, 336 mmol), and the mixture was heated to reflux for 4 h. After cooling to r.t., the solvent was evaporated under reduced pressure. The resulting residue was added water (100 mL), and the pH was adjusted to pH 7 using 6 N HCl. The solid was filtered and washed with water and ether to give a yellow solid (8 g, 95%).

***tert*-Butyl ((2,4-dimethoxy-6-methylpyridin-3-yl)methyl)carbamate (B14a)**

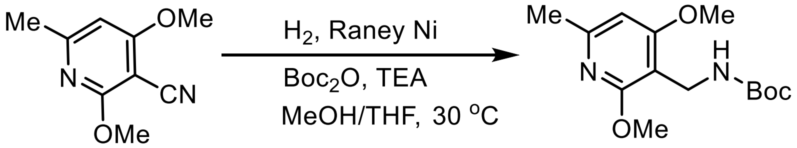

To a solution of **B13a** (10.0 g, 56 mmol) in THF (260 mL) and MeOH (260 mL) were added Raney Ni (water closure, approximately 10 g), TEA (29.0 g, 280 mmol) and Boc_2O (36.8 g, 168 mmol). The resulting suspension was evacuated and backfilled with argon three times. A hydrogen balloon was added and the flask was evacuated and backfilled three times with hydrogen. The resulting suspension was heated in a 30 °C oil bath for 48 h. The mixture was then cooled to rt, vented, and the suspension was filtered through a pad of celite. The filtrate was concentrated *in vacuo* to afford reddish brown oil (13.9 g, 88%). It was used in the following reaction without any further purification.

3-(Aminomethyl)-4-methoxy-6-methylpyridin-2(1*H*)-one hydrogen chloride (B7).

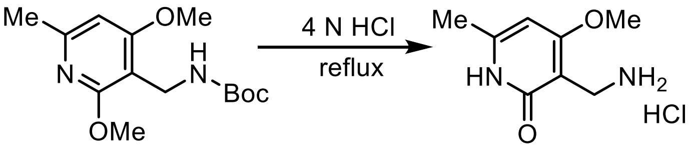

The crude product **B14a** (13.9 g) from previous step was added 4 N HCl (90 mL), the mixture was refluxed for 5 h. After cooling to rt, the water was removed *in vacuo.* The resulting residue was added cold ethanol (50 mL), and stirred at rt for 30 min to allow product precipitation. The solid was filtered and washed with cold ethanol to give white

solid (8.2 g, 82%). ^{1}H NMR (600 MHz, DMSO-d_6) δ (ppm): 11.74 (br, 1H), 7.90 (br, 3H), 6.19 (s, 1H), 3.85 (s, 3H), 3.74-3.71 (q, *J*=11.3 Hz, 5.6 Hz, 2H), 2.22 (s, 3H

Spectrum of intermediates and EZH2 inhibitors

^{1}H NMR spectrum of B6a

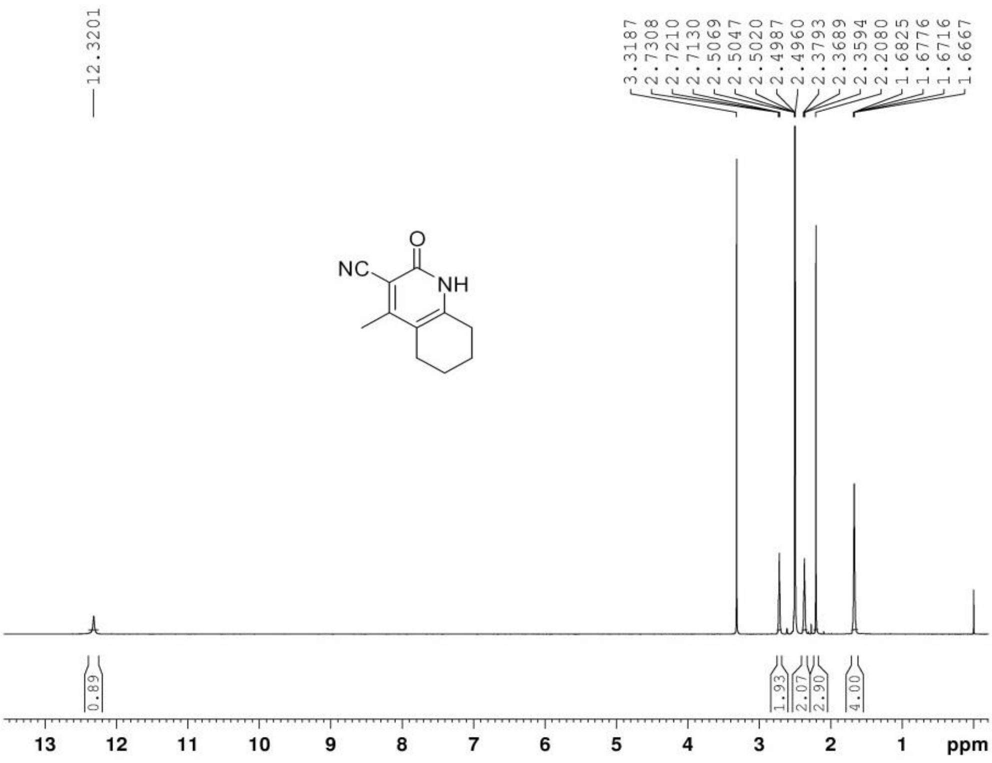

^{1}H NMR spectrum of B6b

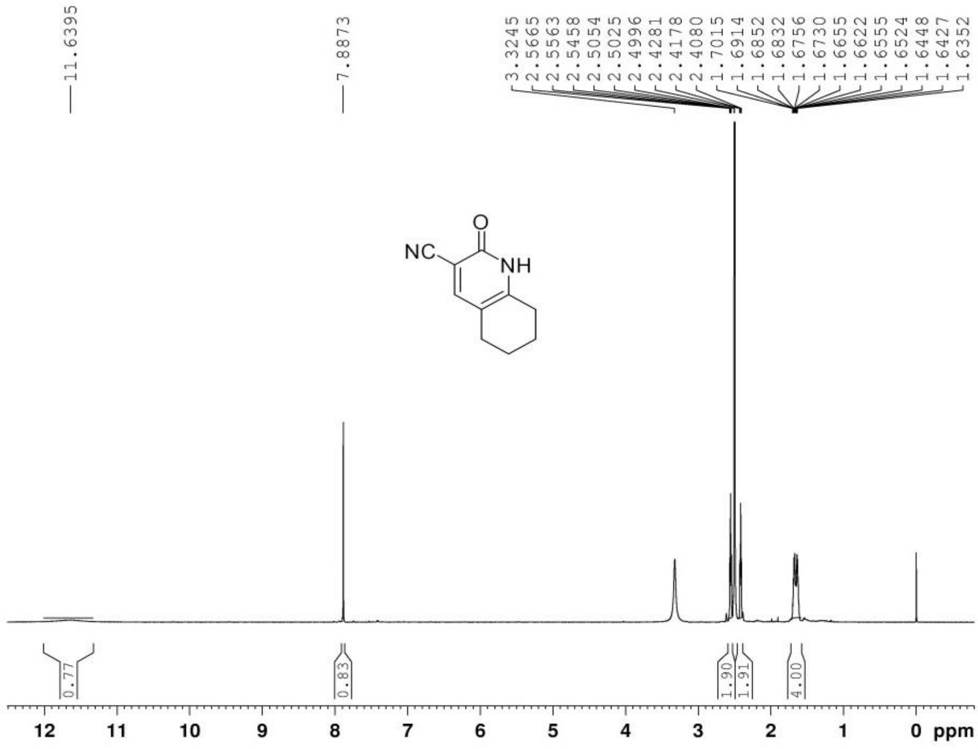

¹H NMR spectrum of B6c

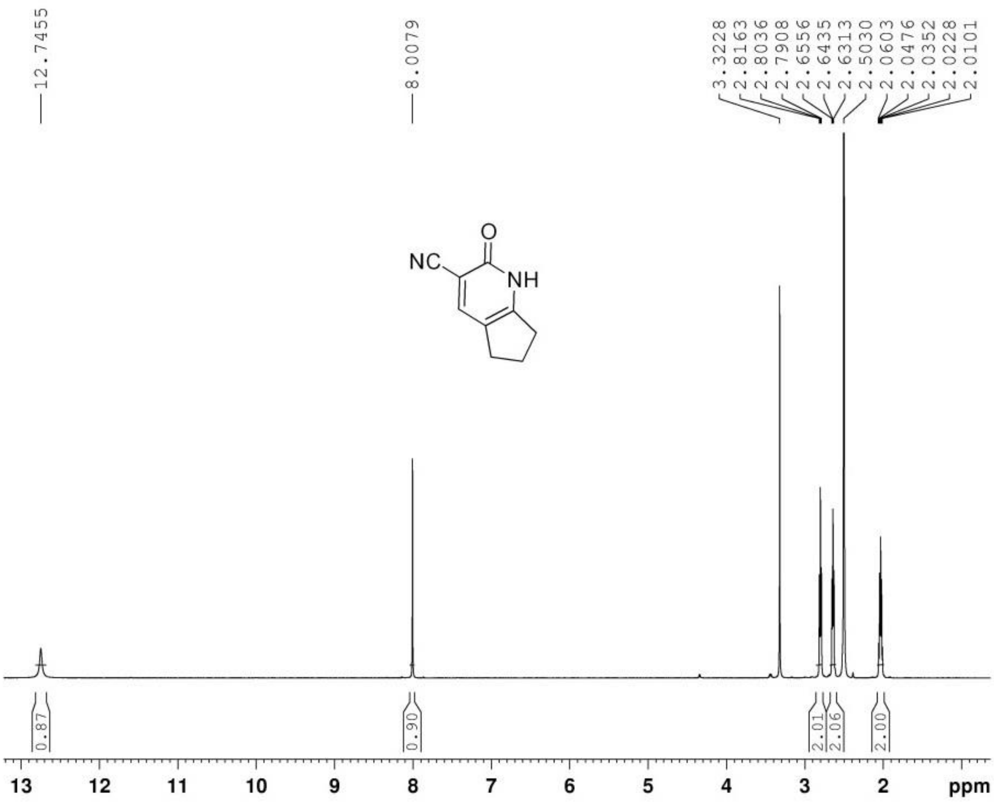

¹H NMR spectrum of B7a

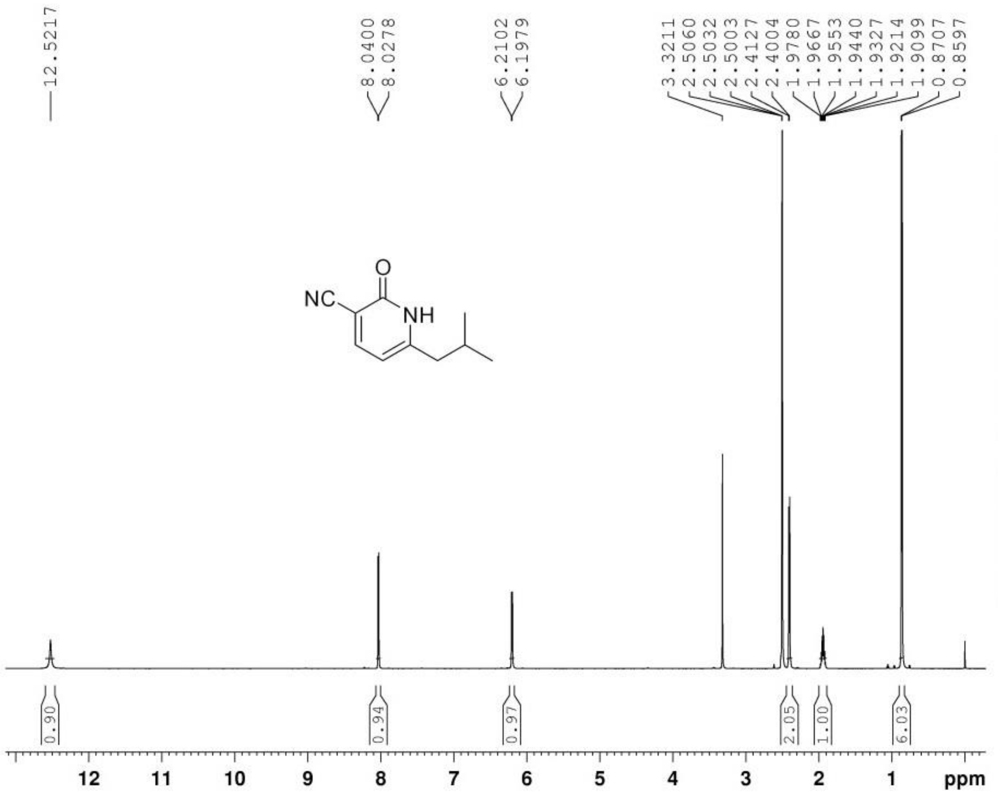

^{1}H NMR spectrum of B7b

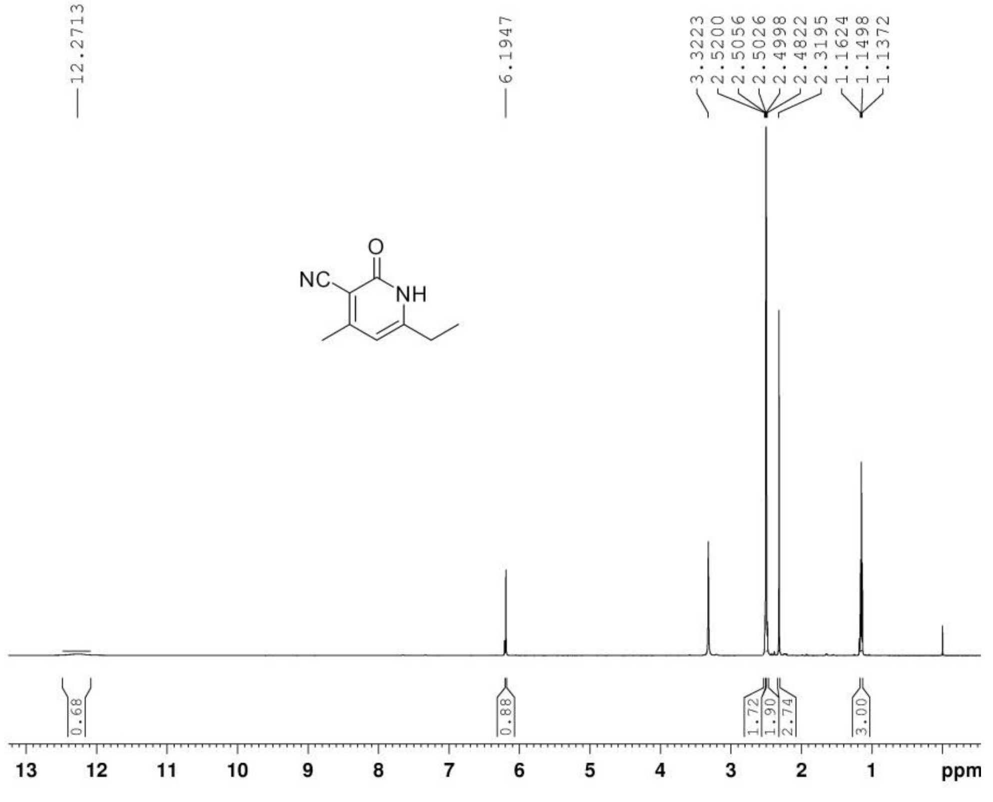

^{1}H NMR spectrum of B7c

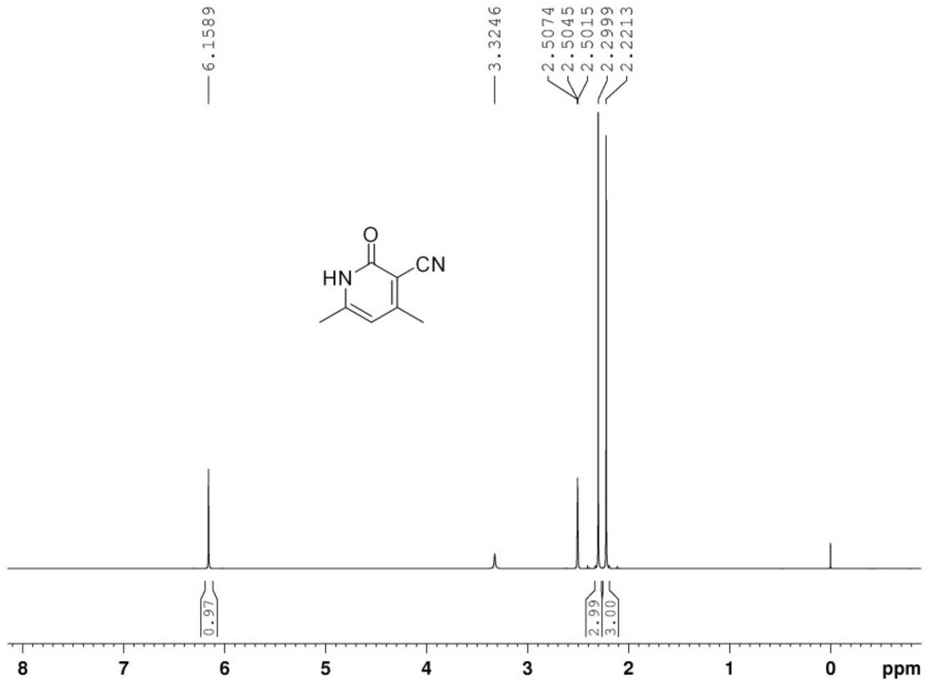

^{1}H NMR spectrum of B6

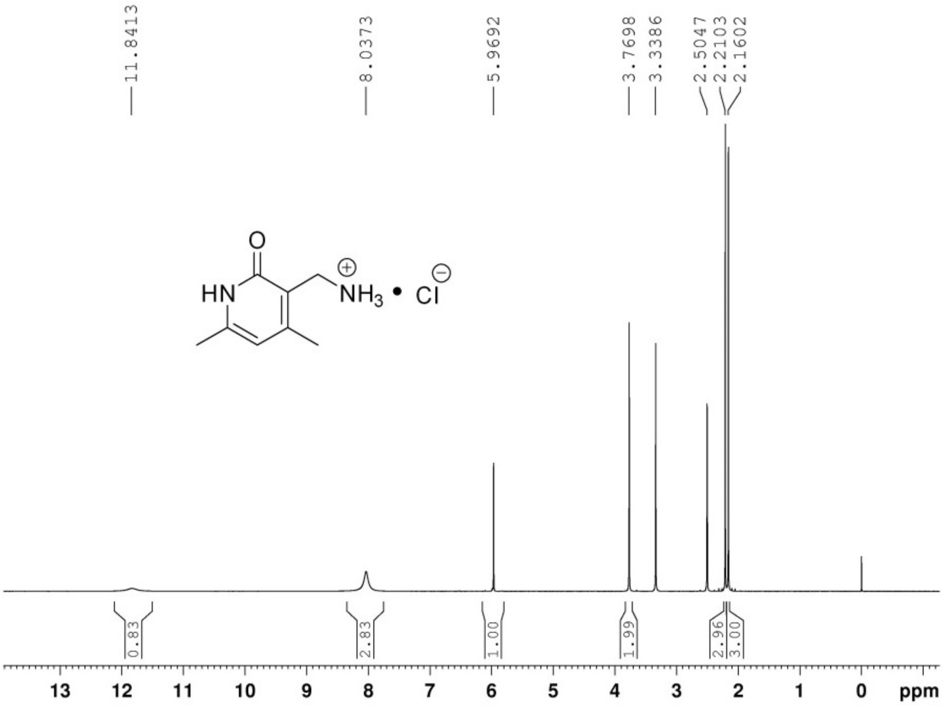

¹H NMR spectrum of B7

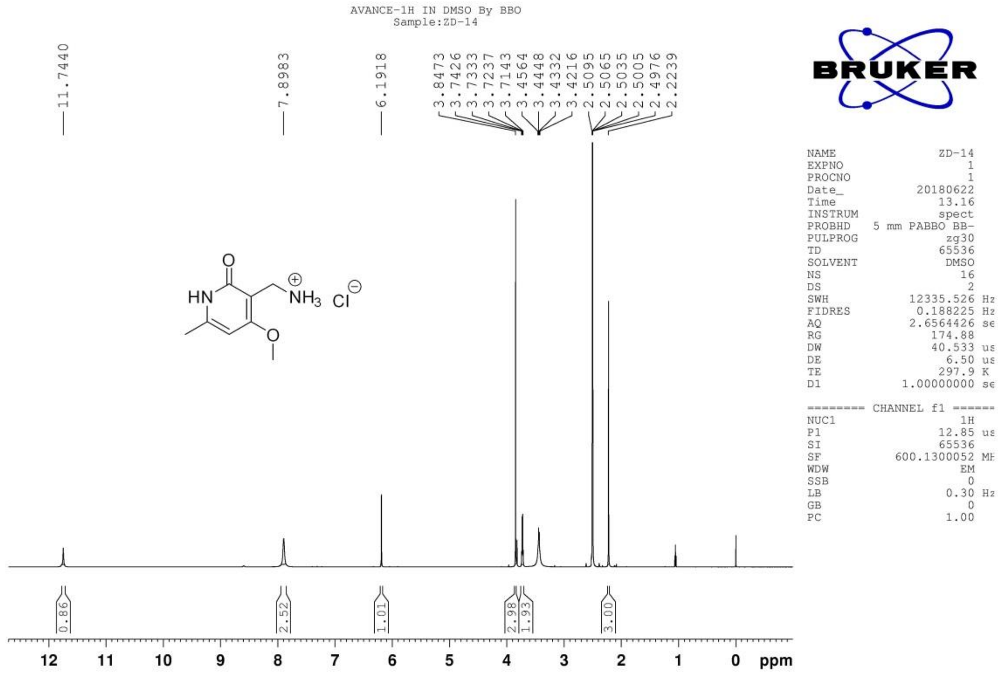

Spectrum of compound 4a

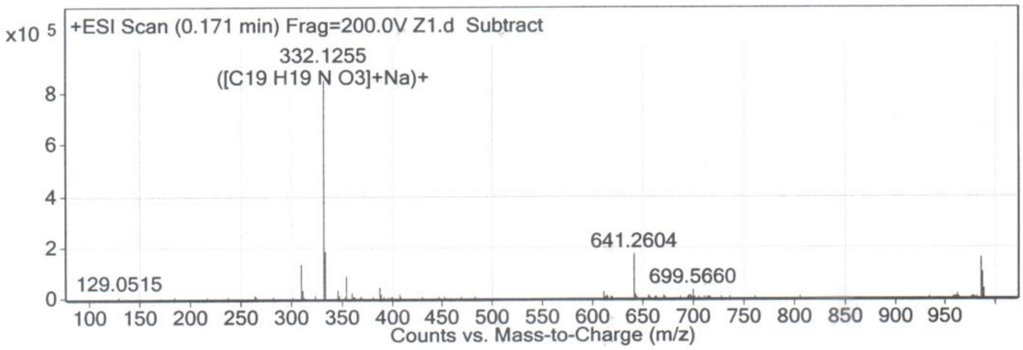

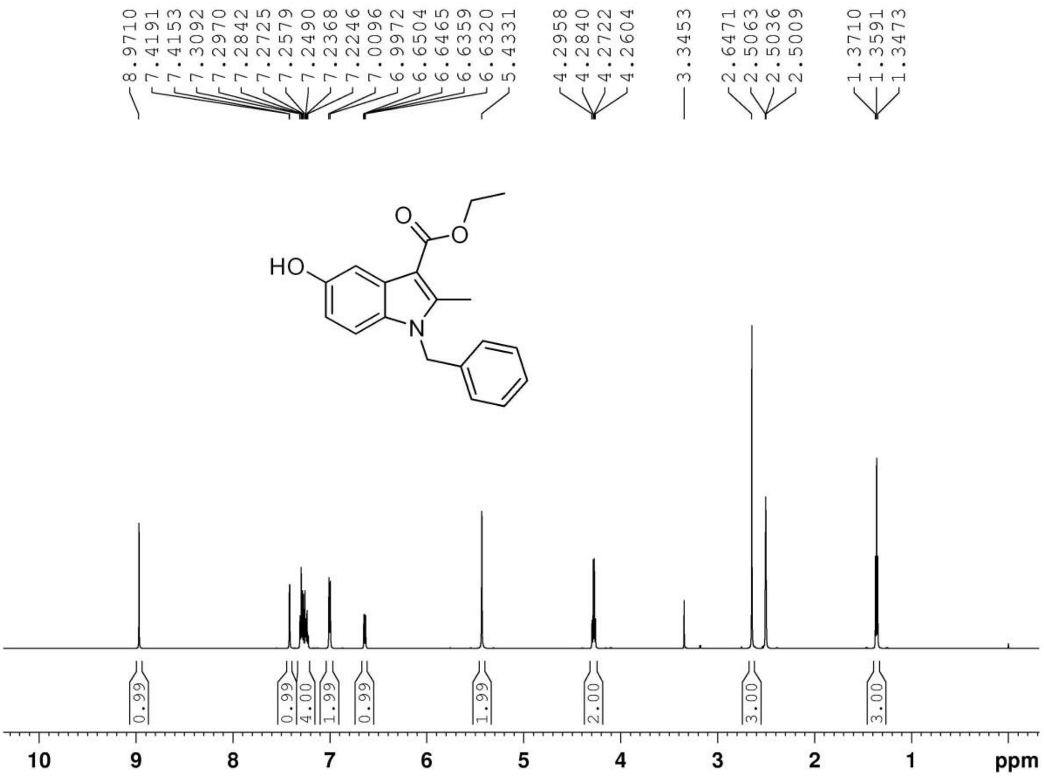
8.9710
7.4191
7.4153
7.3092
7.2970
7.2842
7.2725
7.2579
7.2490
7.2368
7.2246
7.0096
6.9972
6.6504
6.6465
6.6359
6.6320
5.4331
4.2958
4.2840
4.2722
4.2604
3.3453
2.6471
2.5063
2.5036
2.5009
1.3710
1.3591
1.3473
HO
O
O
N
0.99
0.99
4.00
1.99
0.99
1.99
2.00
3.00
3.00
10
9
8
7
6
5
4
3
2
1
ppm

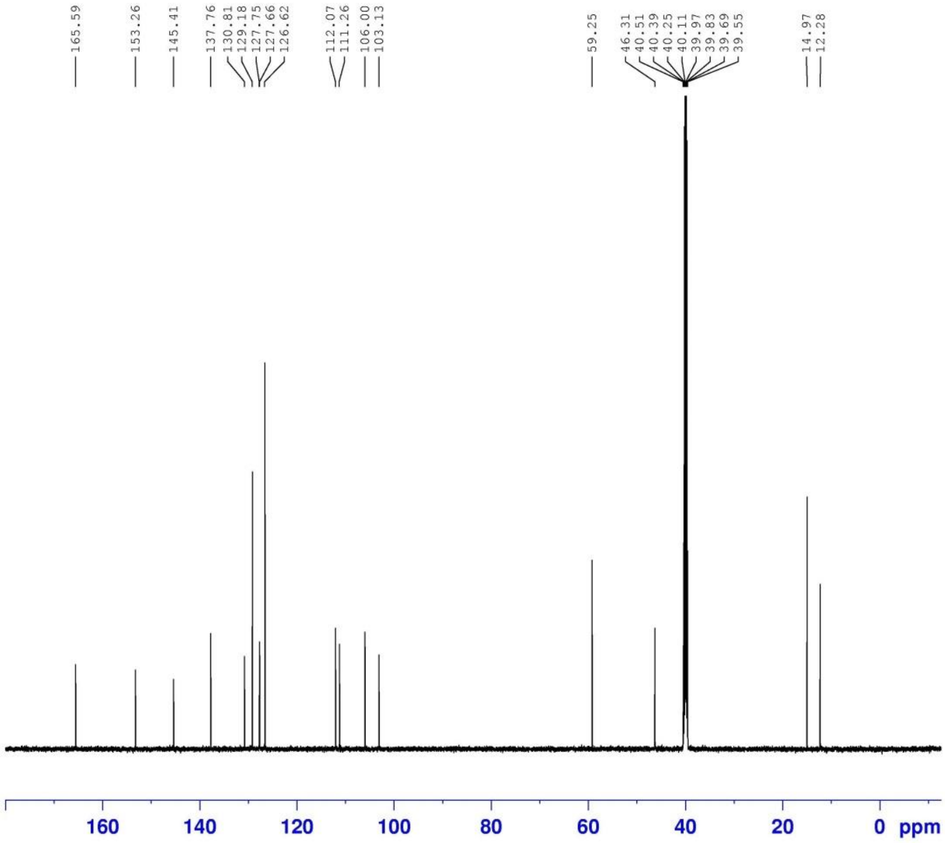
165.59
153.26
145.41
137.76
130.81
129.18
127.75
127.66
126.62
112.07
111.26
106.00
103.13
59.25
46.31
40.51
40.39
40.25
40.11
39.97
39.83
39.69
39.55
14.97
12.28
160
140
120
100
80
60
40
20
0
ppm

Spectrum of compound 4b

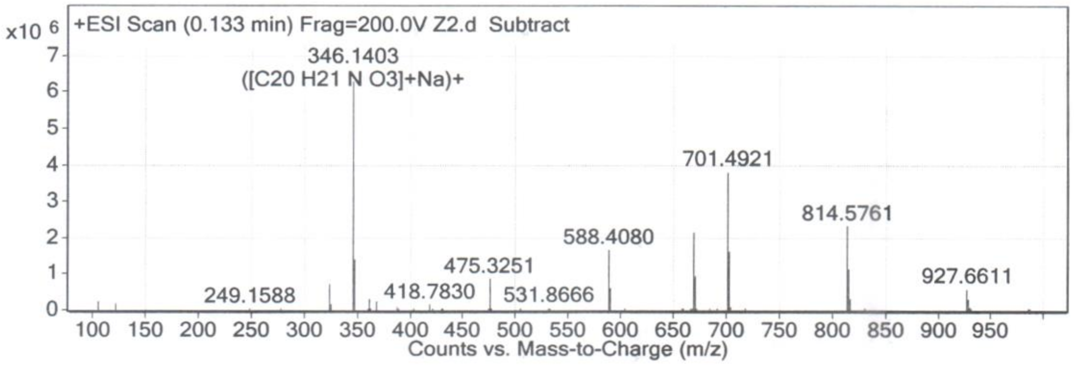

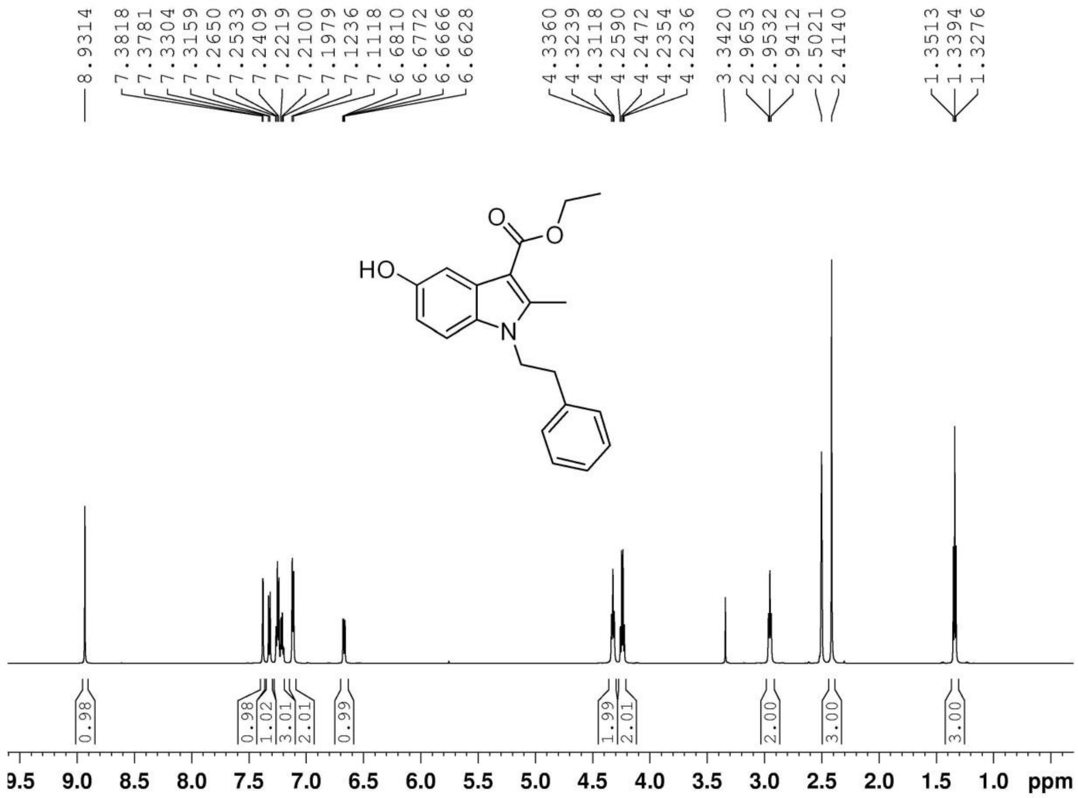

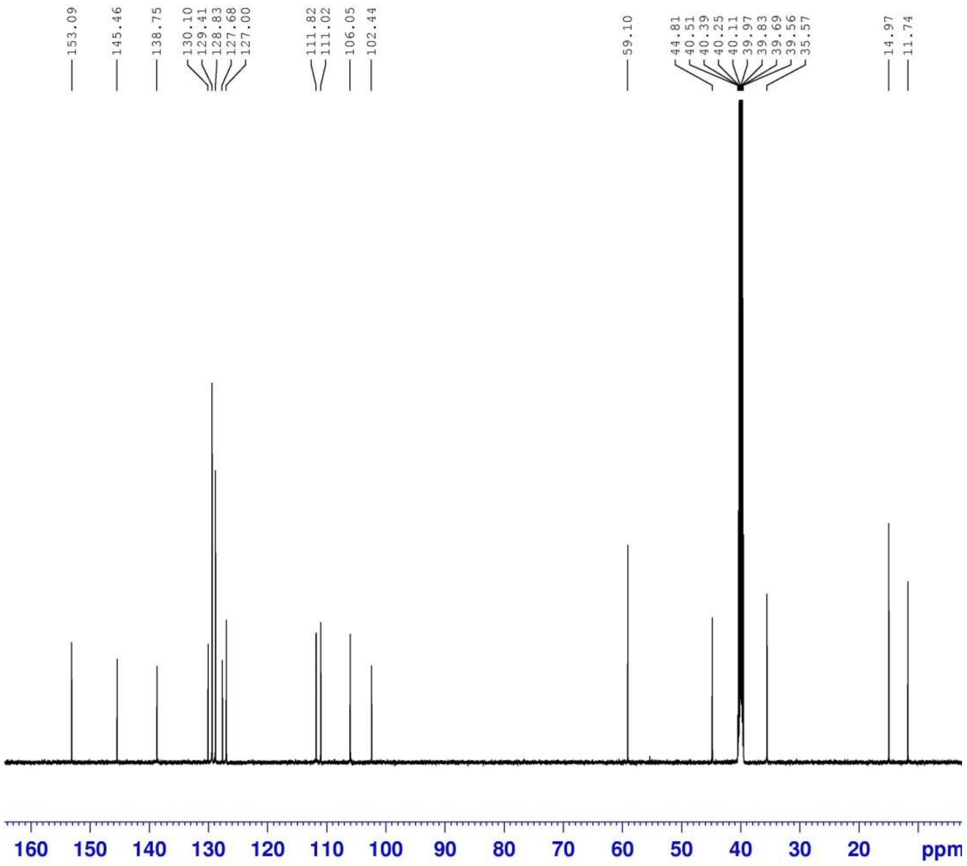

Spectrum of compound 4c

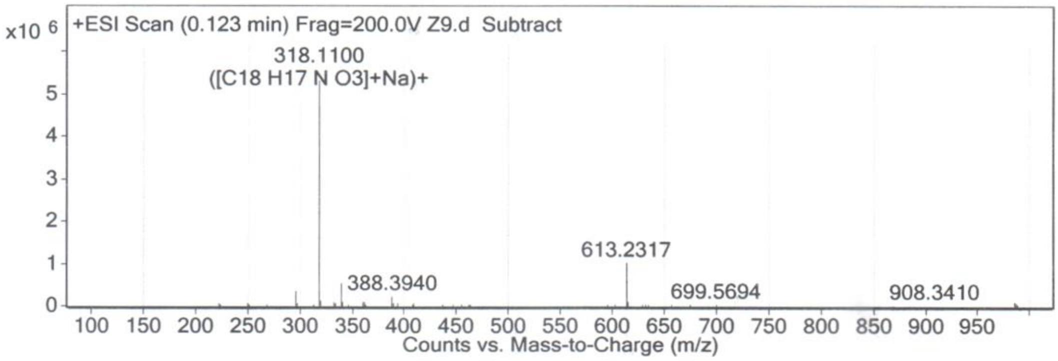

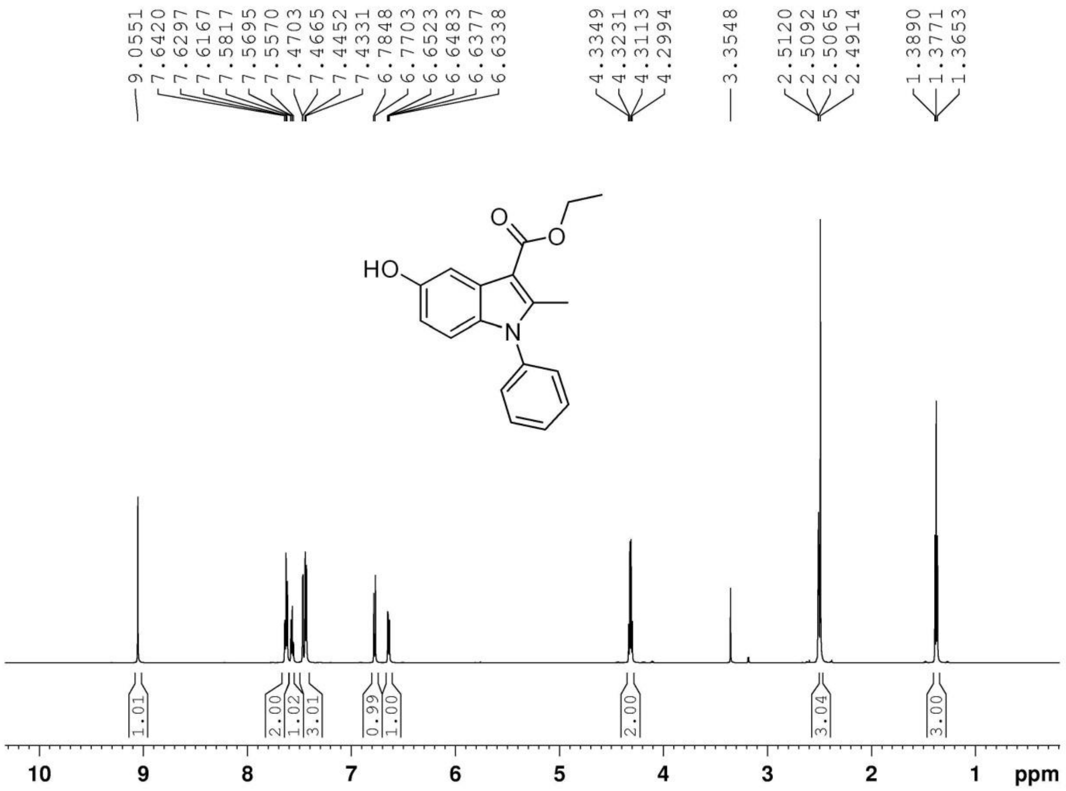
9.0551
7.6420
7.6297
7.6167
7.5817
7.5695
7.5570
7.4703
7.4665
7.4452
7.4331
6.7848
6.7703
6.6523
6.6483
6.6377
6.6338
4.3349
4.3231
4.3113
4.2994
3.3548
2.5120
2.5092
2.5065
2.4914
1.3890
1.3771
1.3653
HO
O
O
N
1.01
2.00
1.02
3.01
0.99
1.00
2.00
3.04
3.00
10
9
8
7
6
5
4
3
2
1
ppm

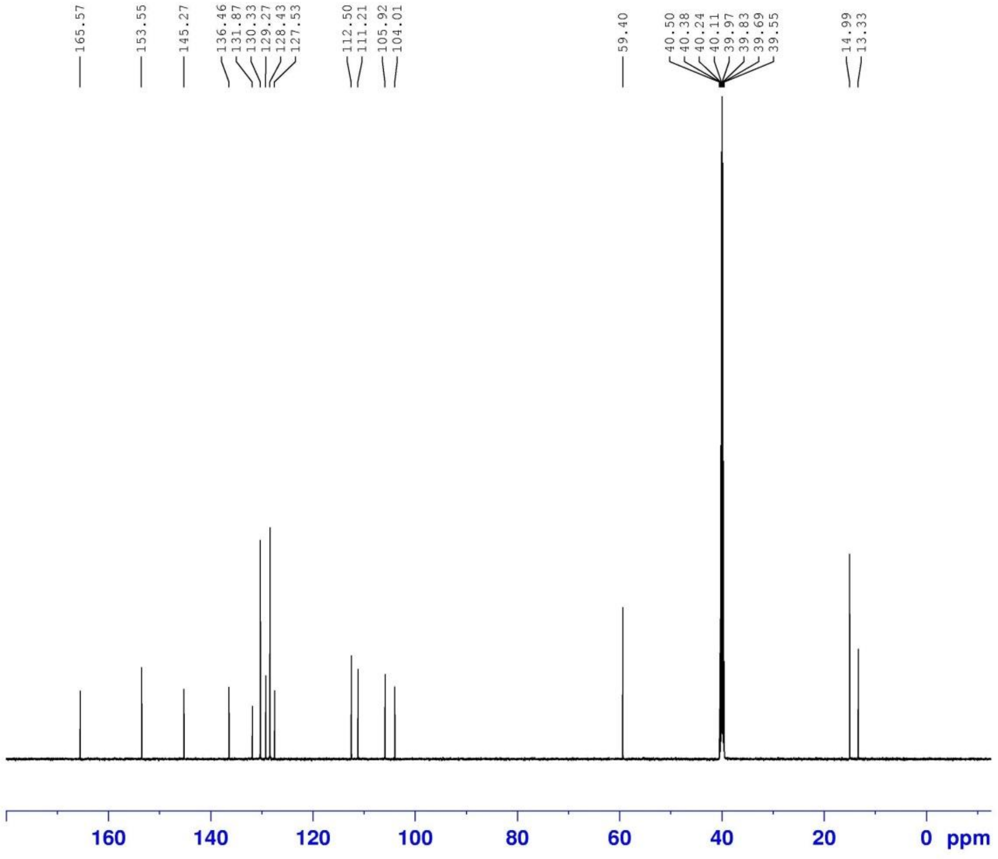
165.57
153.55
145.27
136.46
131.87
130.33
129.27
128.43
127.53
112.50
111.21
105.92
104.01
59.40
40.50
40.38
40.24
40.11
39.97
39.83
39.69
39.55
14.99
13.33
160
140
120
100
80
60
40
20
0
ppm

Spectrum of compound 4d

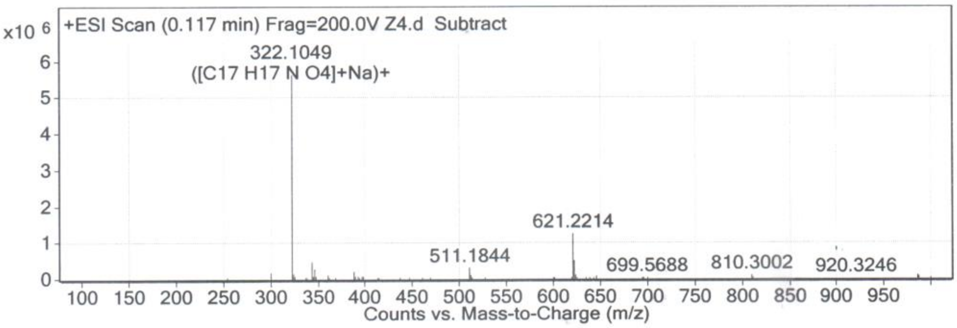

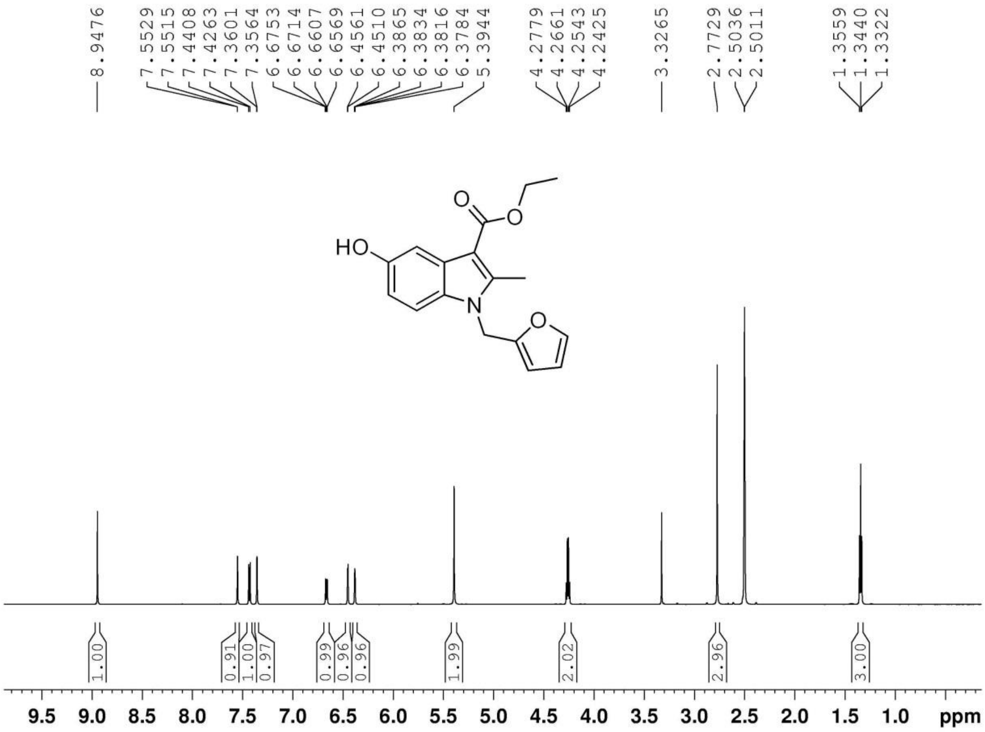

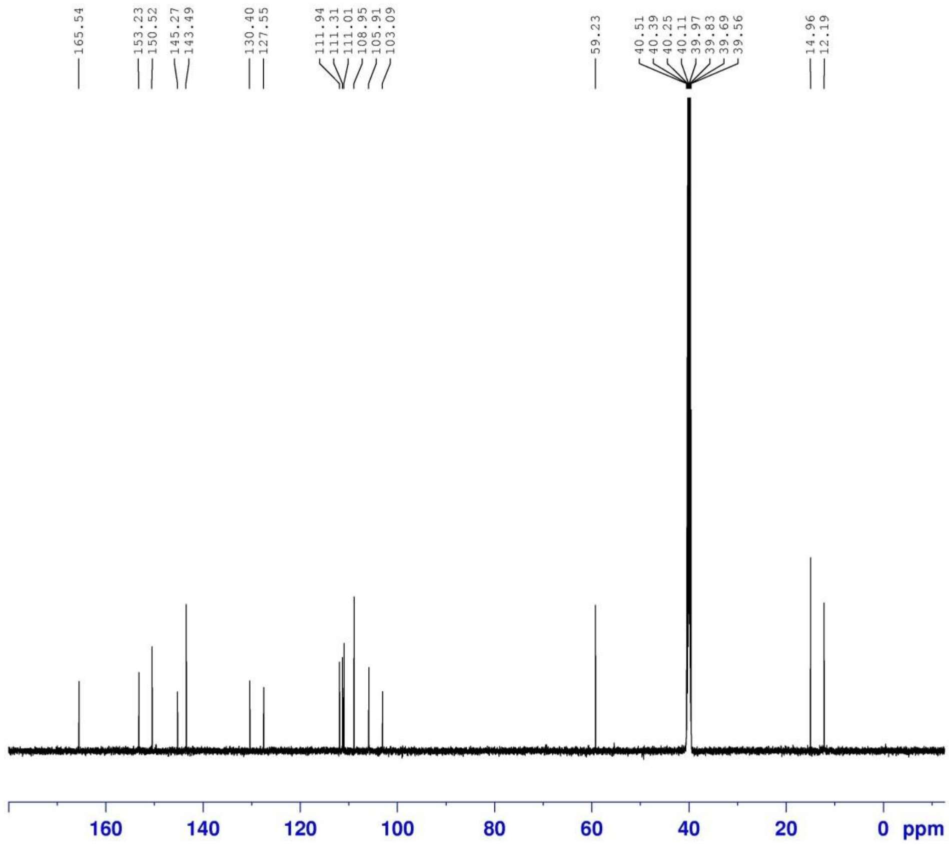

Spectrum of compound 4e

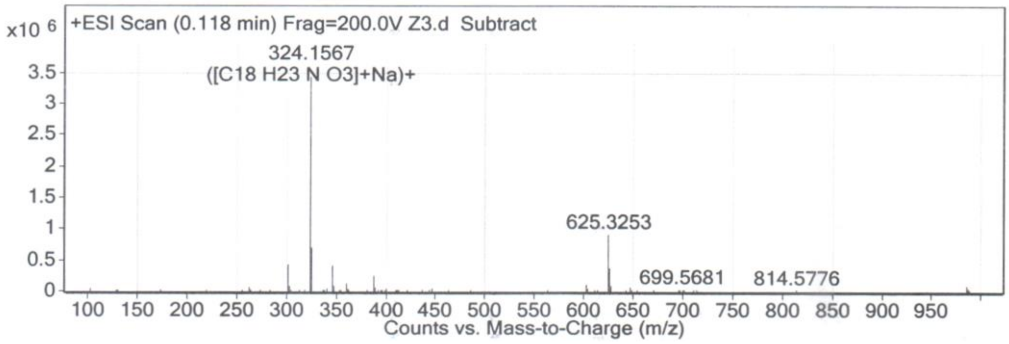

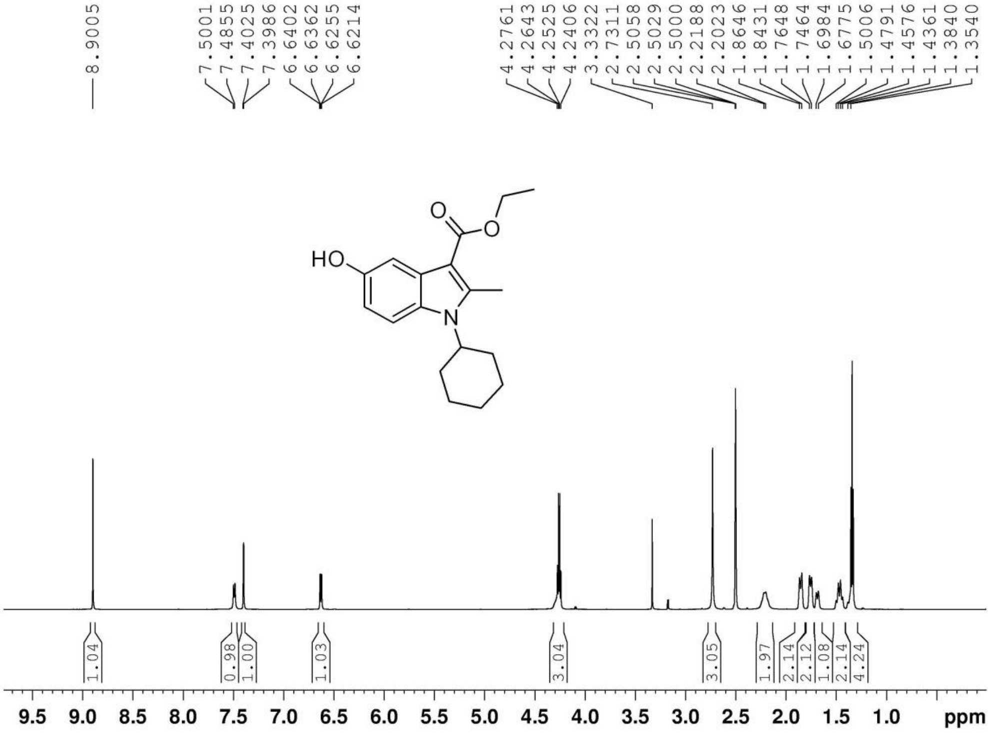
8.9005
7.5001
7.4855
7.4025
7.3986
6.6402
6.6362
6.6255
6.6214
4.2761
4.2643
4.2525
4.2406
3.3322
2.7311
2.5058
2.5029
2.5000
2.2188
2.2023
1.8646
1.8431
1.7648
1.7464
1.6984
1.6775
1.5006
1.4791
1.4576
1.4361
1.3840
1.3540
HO
O
O
N
1.04
0.98
1.00
1.03
3.04
3.05
1.97
2.14
2.12
1.08
2.14
4.24
9.5 9.0 8.5 8.0 7.5 7.0 6.5 6.0 5.5 5.0 4.5 4.0 3.5 3.0 2.5 2.0 1.5 1.0 ppm

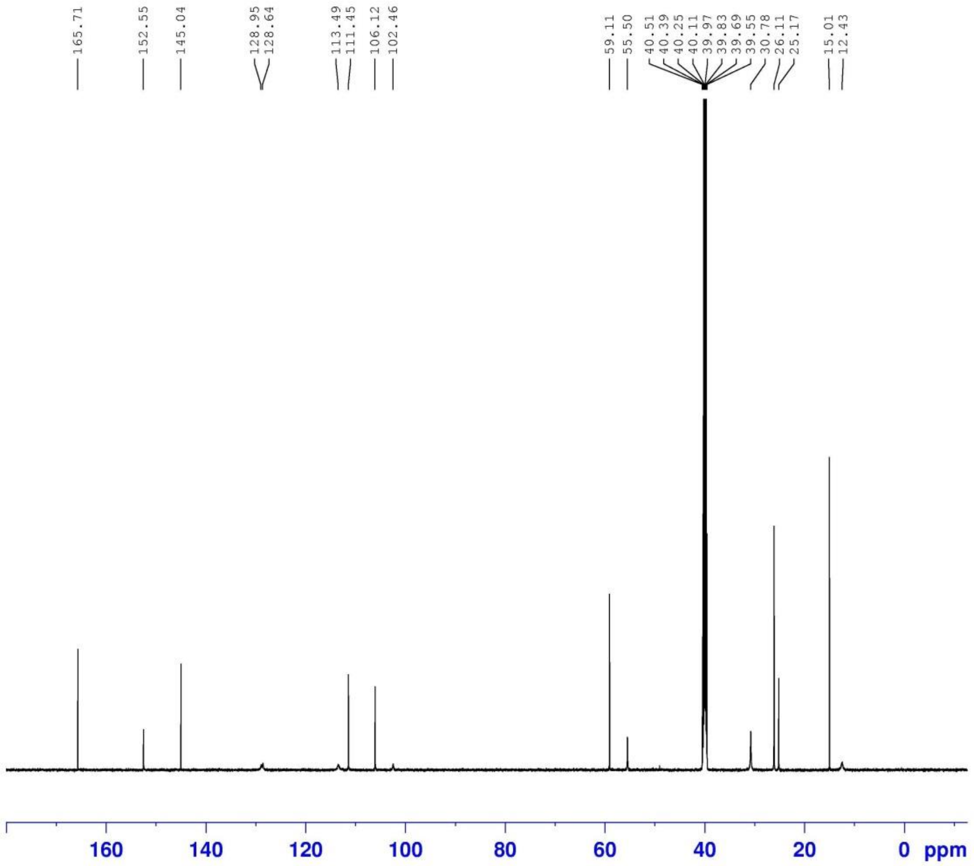
165.71
152.55
145.04
128.95
128.64
113.49
111.45
106.12
102.46
59.11
55.50
40.51
40.39
40.25
40.11
39.97
39.83
39.69
39.55
30.78
26.11
25.17
15.01
12.43
160 140 120 100 80 60 40 20 0 ppm

Spectrum of compound 4f

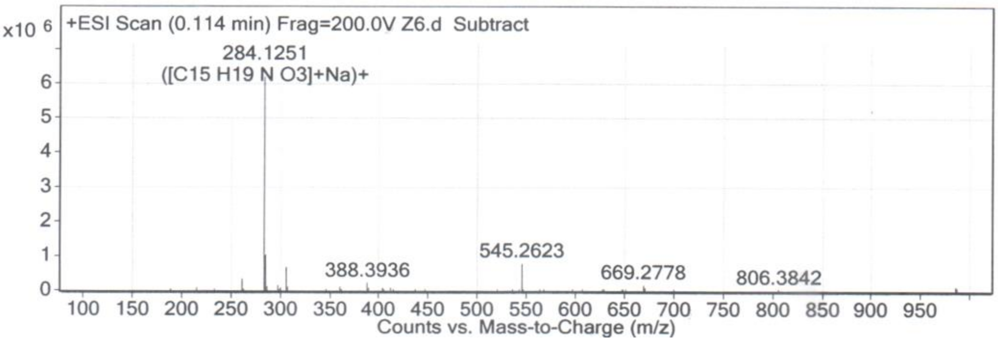

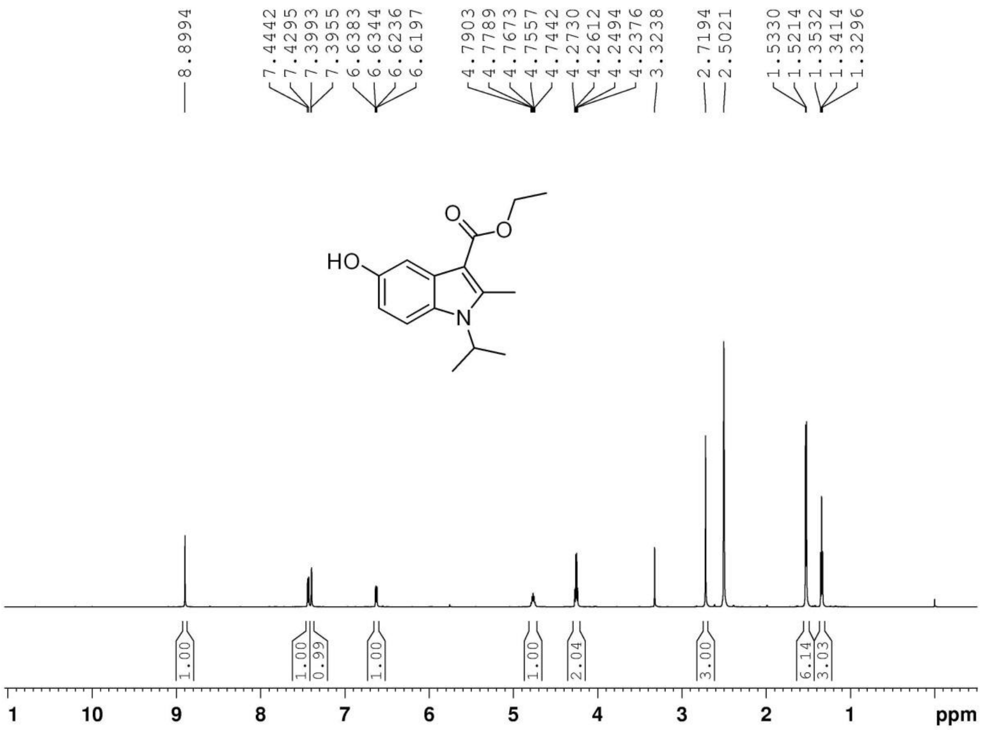

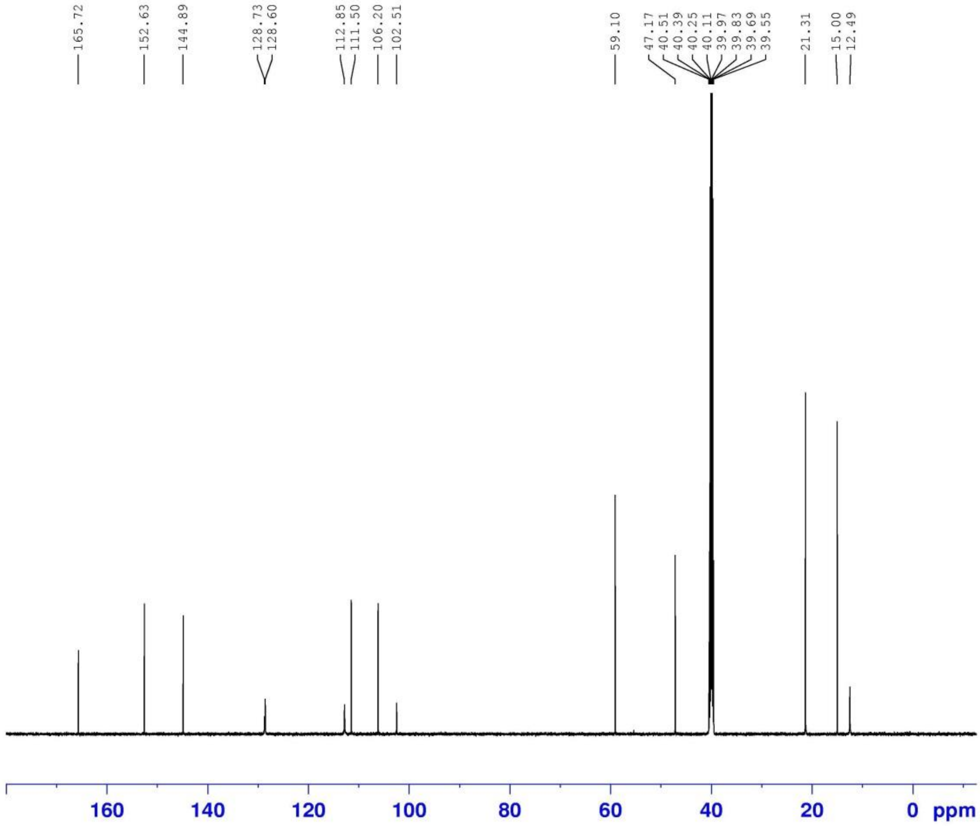

spectrum of compound 4g

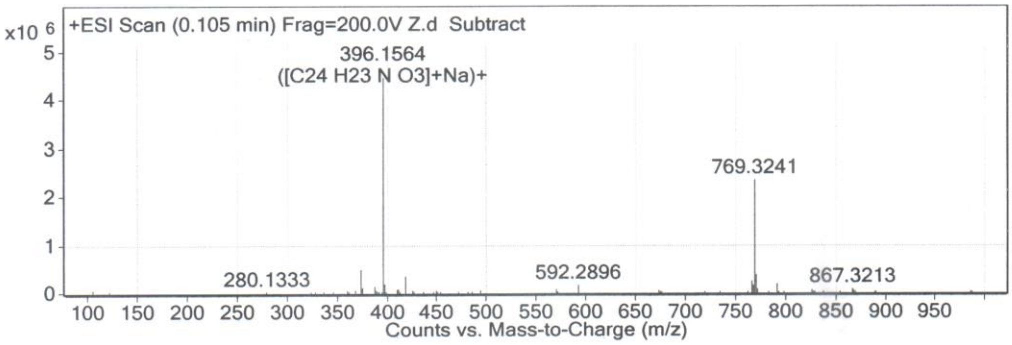

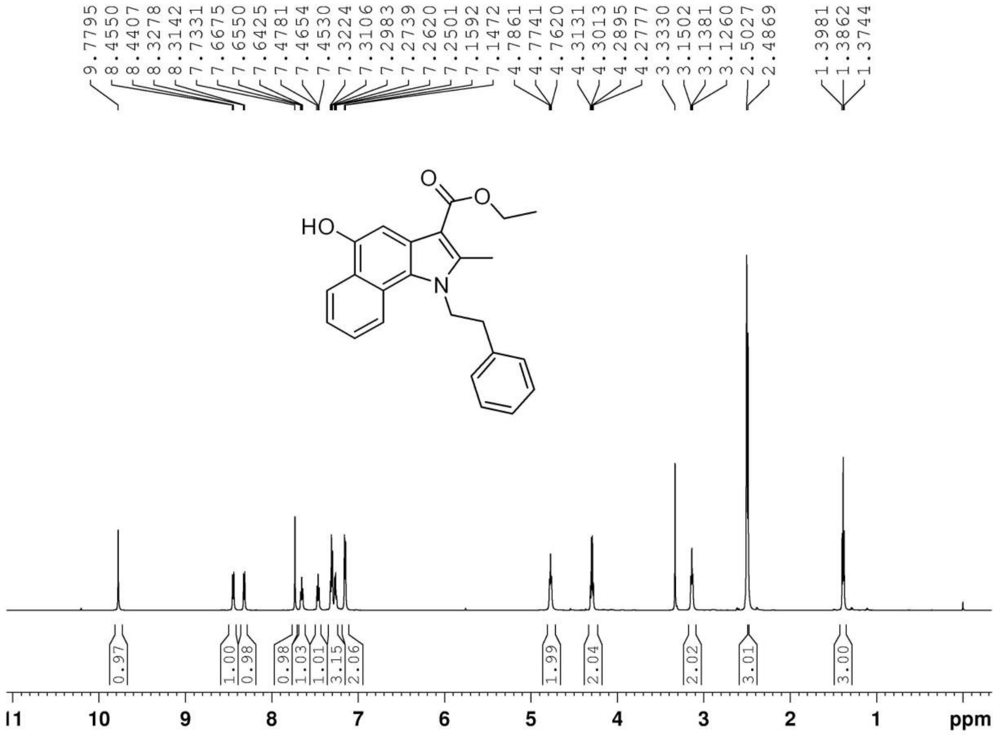
9.7795
8.4550
8.4407
8.3278
8.3142
7.7331
7.6675
7.6550
7.6425
7.4781
7.4654
7.4530
7.3224
7.3106
7.2983
7.2739
7.2620
7.2501
7.1592
7.1472
4.7861
4.7741
4.7620
4.3131
4.3013
4.2895
4.2777
3.3330
3.1502
3.1381
3.1260
2.5027
2.4869
1.3981
1.3862
1.3744
HO
O
O
N
0.97
1.00
0.98
0.98
1.03
1.01
3.15
2.06
1.99
2.04
2.02
3.01
3.00
11
10
9
8
7
6
5
4
3
2
1
ppm

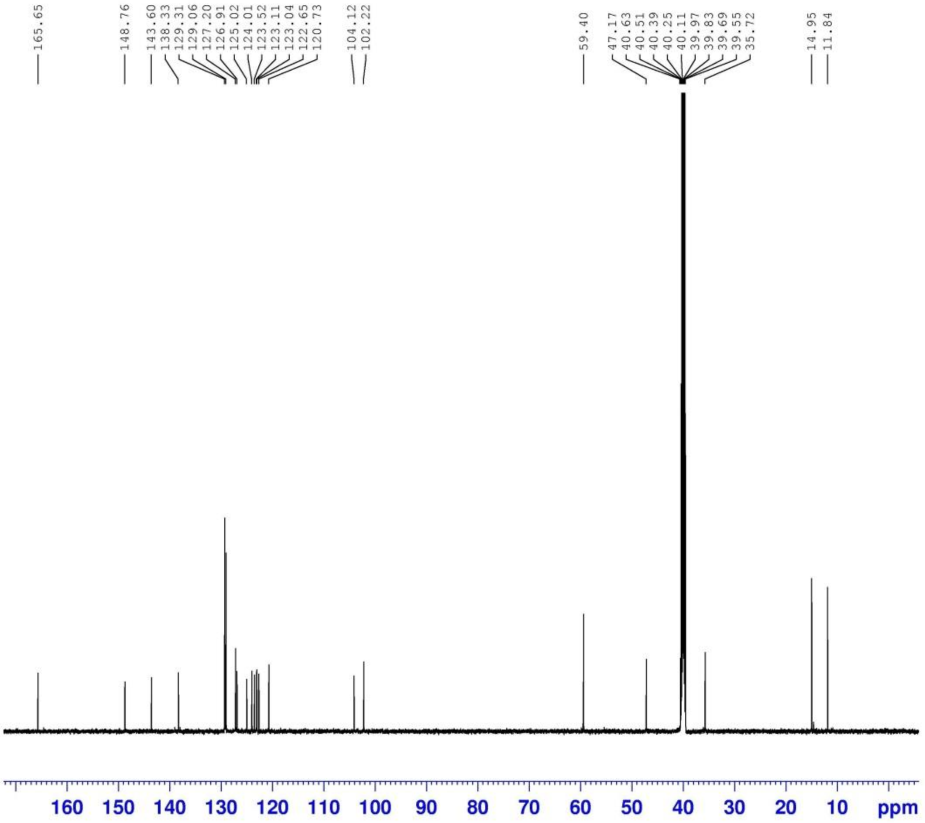
165.65
148.76
143.60
138.33
129.31
129.06
127.20
126.91
125.02
124.01
123.52
123.11
123.04
122.65
120.73
104.12
102.22
59.40
47.17
40.63
40.51
40.39
40.25
40.11
39.97
39.83
39.69
39.55
35.72
14.95
11.84
160
150
140
130
120
110
100
90
80
70
60
50
40
30
20
10
ppm

Spectrum of L-01

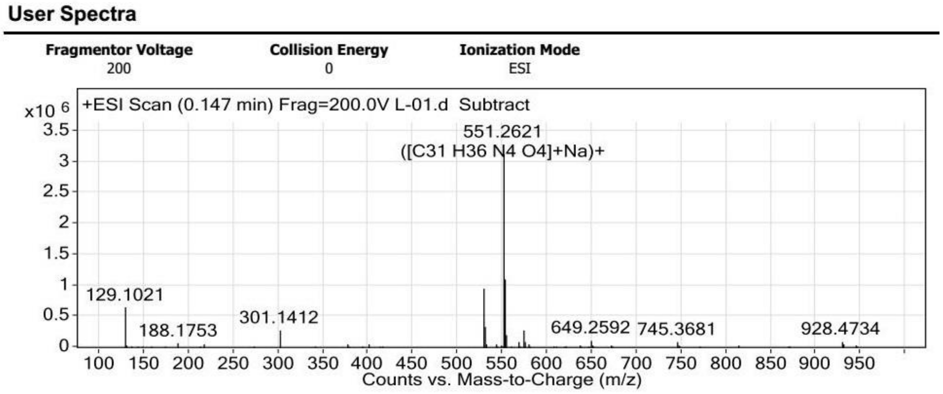

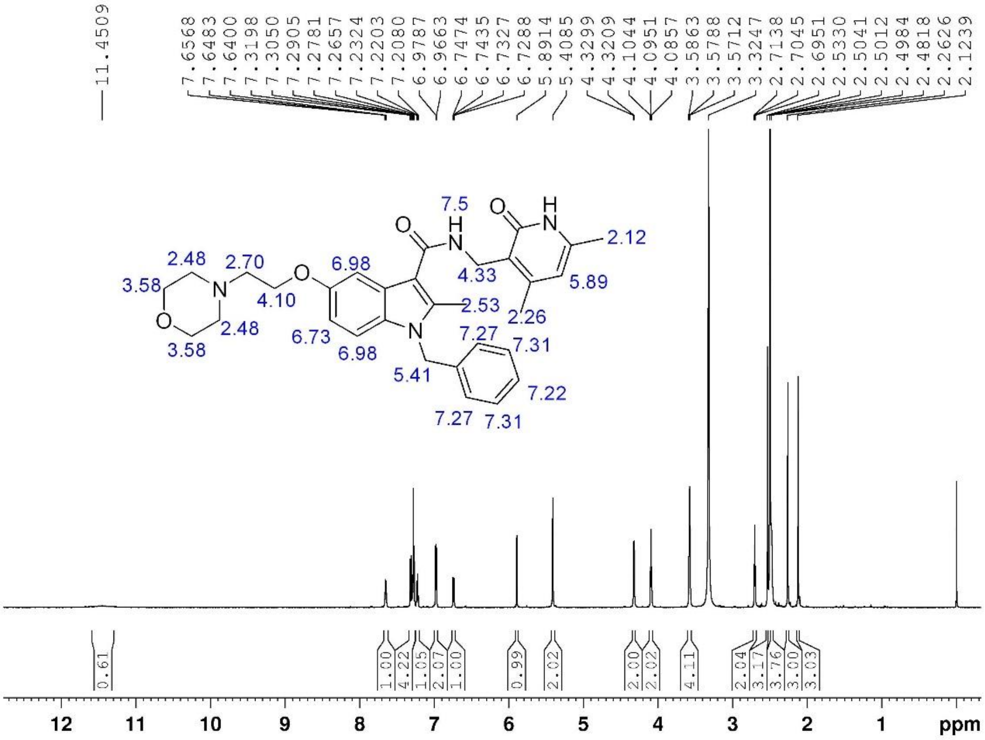

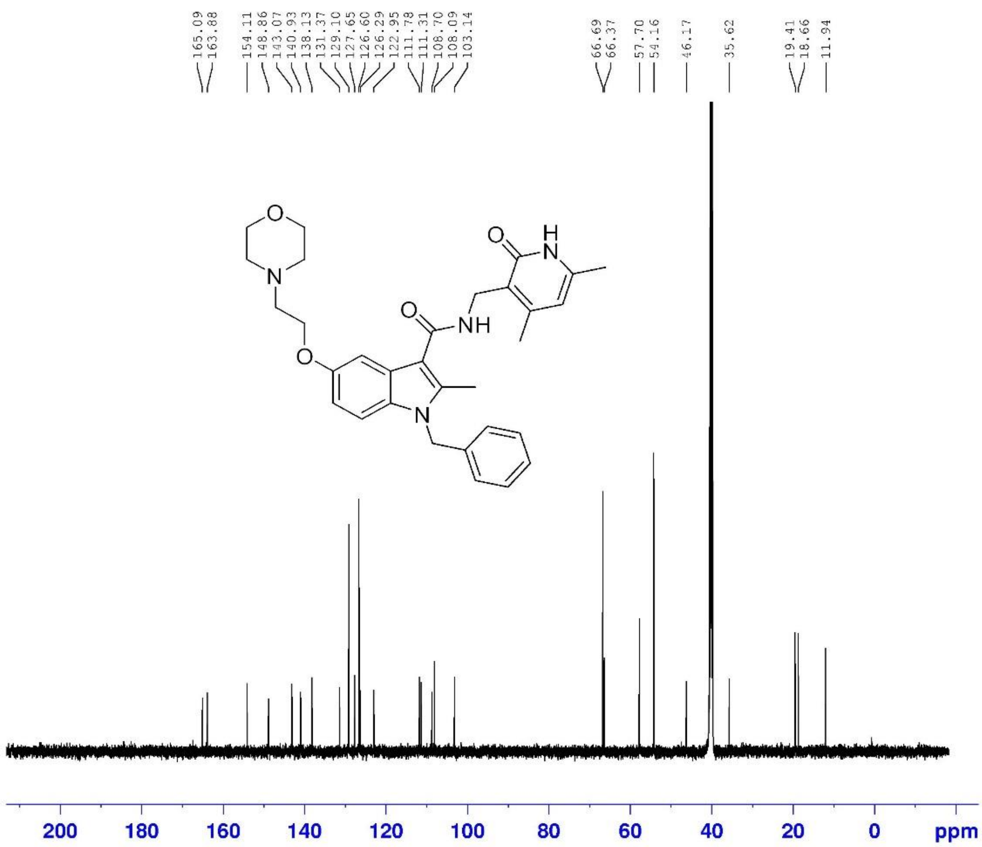
165.09
163.88
154.11
148.86
143.07
140.93
138.13
131.37
129.10
127.65
126.60
126.29
122.95
111.78
111.31
108.70
108.09
103.14
66.69
66.37
57.70
54.16
46.17
35.62
19.41
18.66
11.94
O
N
O
O
NH
O
H
N
N
200
180
160
140
120
100
80
60
40
20
0
ppm

Spectrum of L-02

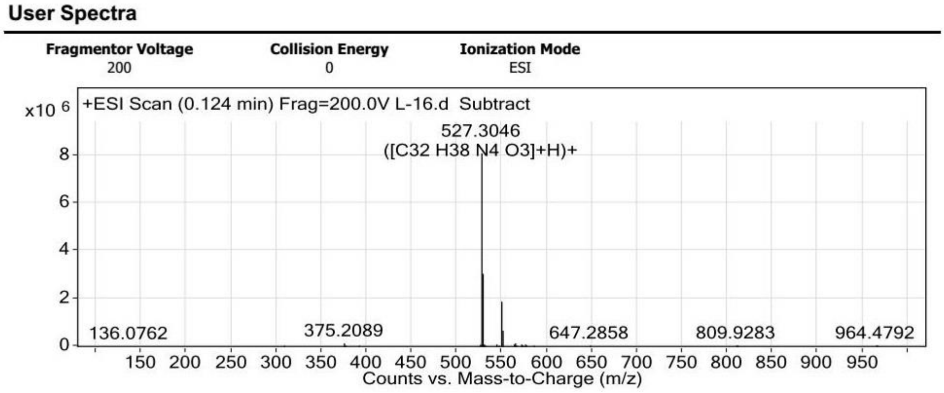

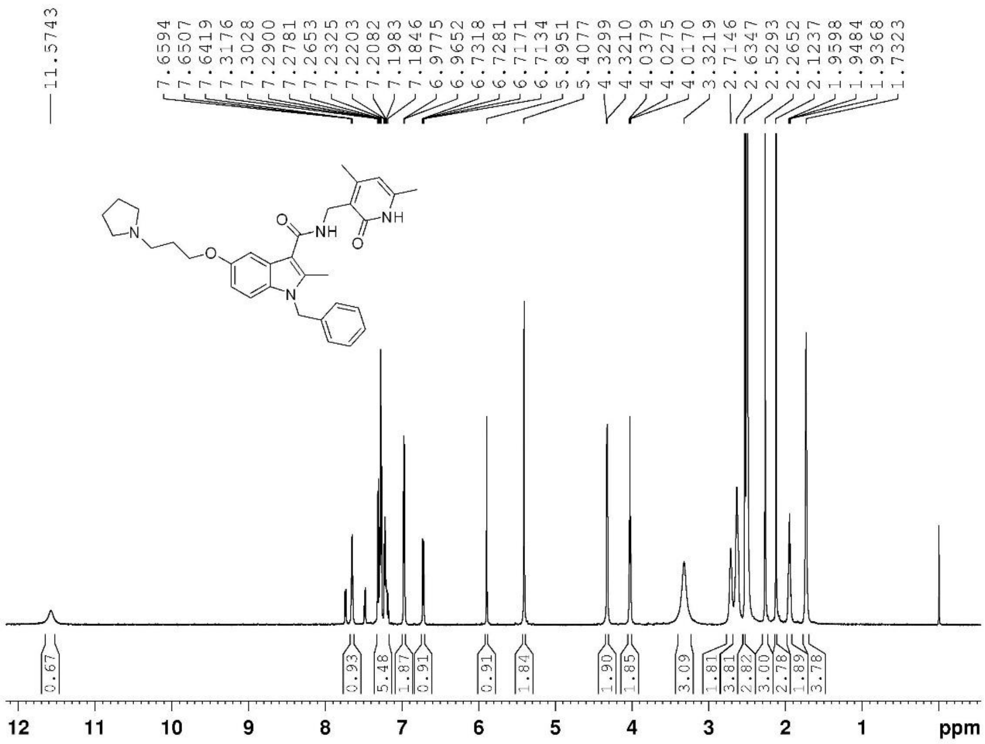

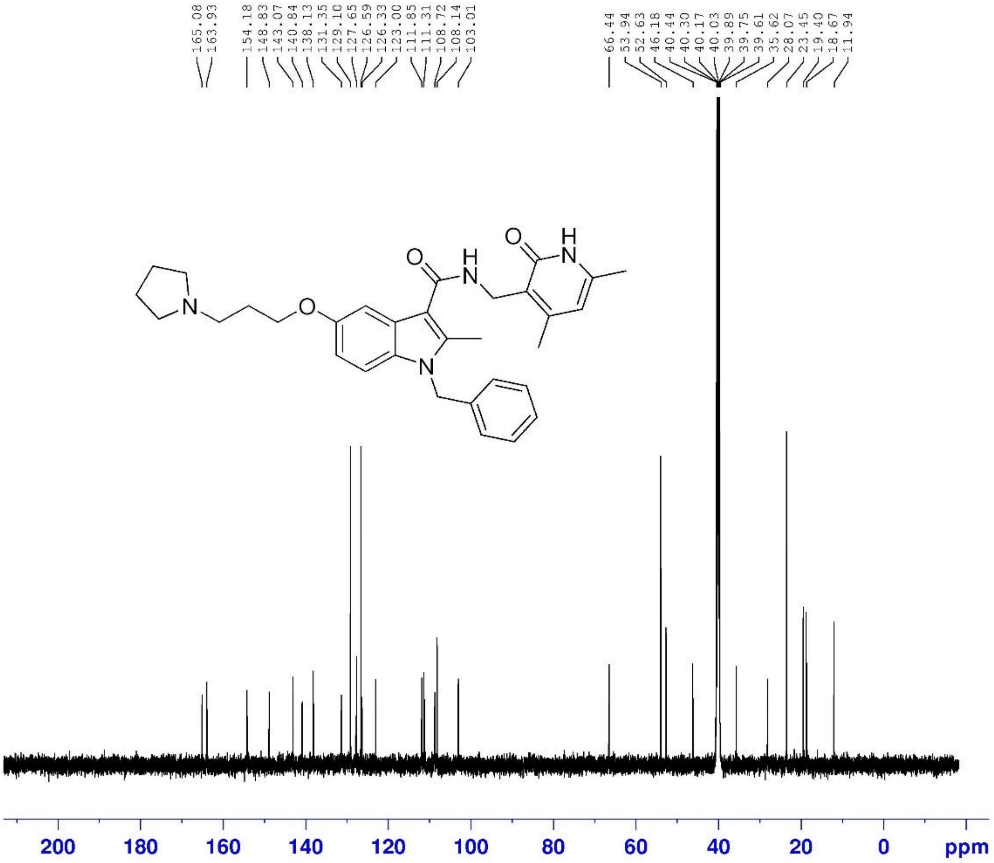

Spectrum of L-03

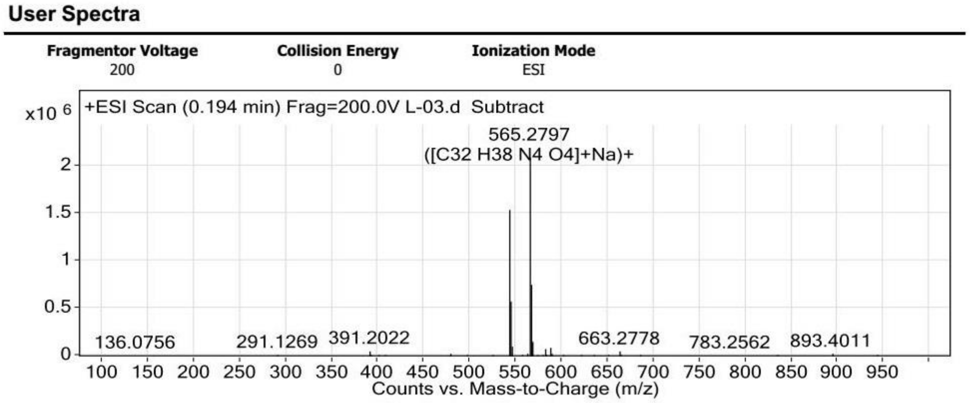

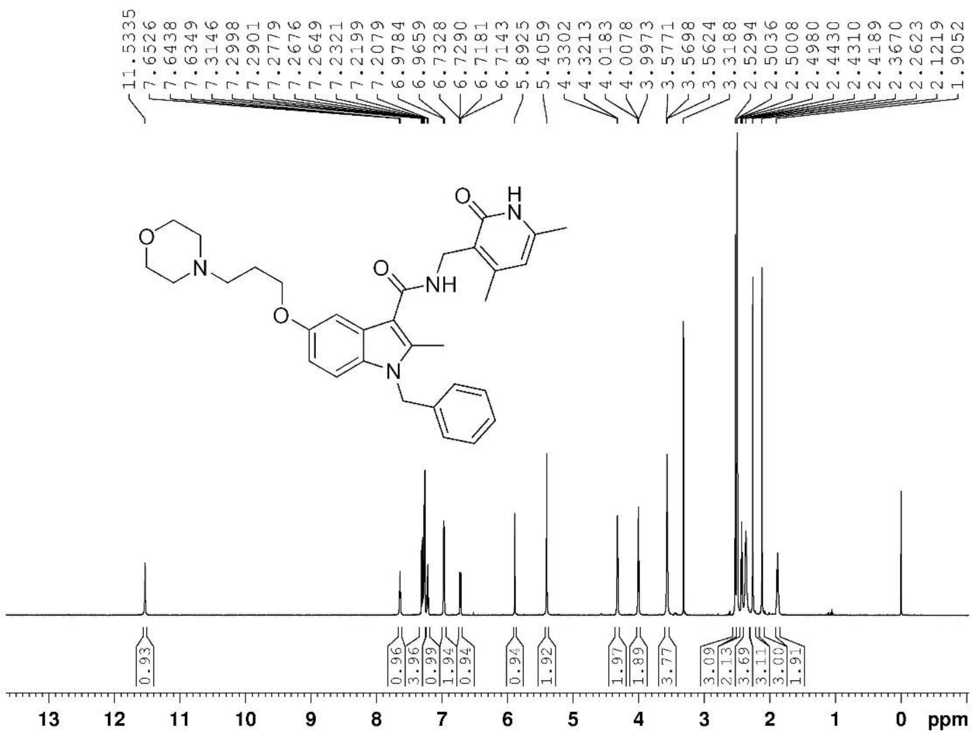
11.5335
7.6526
7.6438
7.6349
7.3146
7.2998
7.2901
7.2779
7.2676
7.2649
7.2321
7.2199
7.2079
6.9784
6.9659
6.7328
6.7290
6.7181
6.7143
5.8925
5.4059
4.3302
4.3213
4.0183
4.0078
3.9973
3.5771
3.5698
3.5624
3.3188
2.5294
2.5036
2.5008
2.4980
2.4430
2.4310
2.4189
2.3670
2.2623
2.1219
1.9052
O
N
H
NH
O
N
O
N
O
0.93
0.96
3.96
0.99
1.94
0.94
0.94
1.92
1.97
1.89
3.77
3.09
2.13
3.69
3.11
3.00
1.91
13
12
11
10
9
8
7
6
5
4
3
2
1
0
ppm

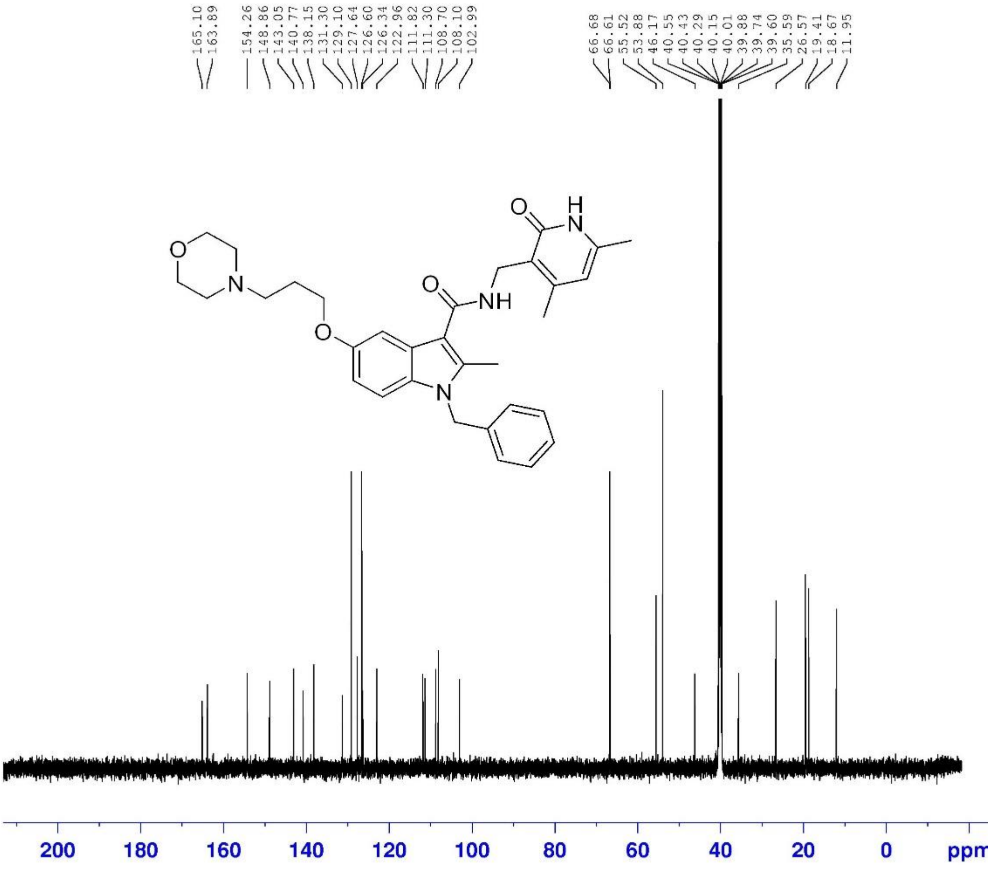
165.10
163.89
154.26
148.86
143.05
140.77
138.15
131.30
129.10
127.64
126.60
126.34
122.96
111.82
111.30
108.70
108.10
102.99
66.68
66.61
55.52
53.88
46.17
40.55
40.43
40.29
40.15
40.01
39.88
39.74
39.60
35.59
26.57
19.41
18.67
11.95
O
N
H
NH
O
N
O
N
O
200
180
160
140
120
100
80
60
40
20
0
ppm

Spectrum of L-04

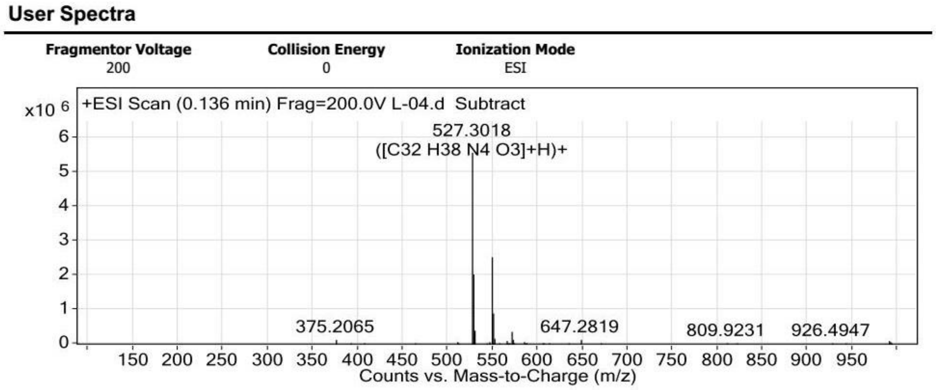

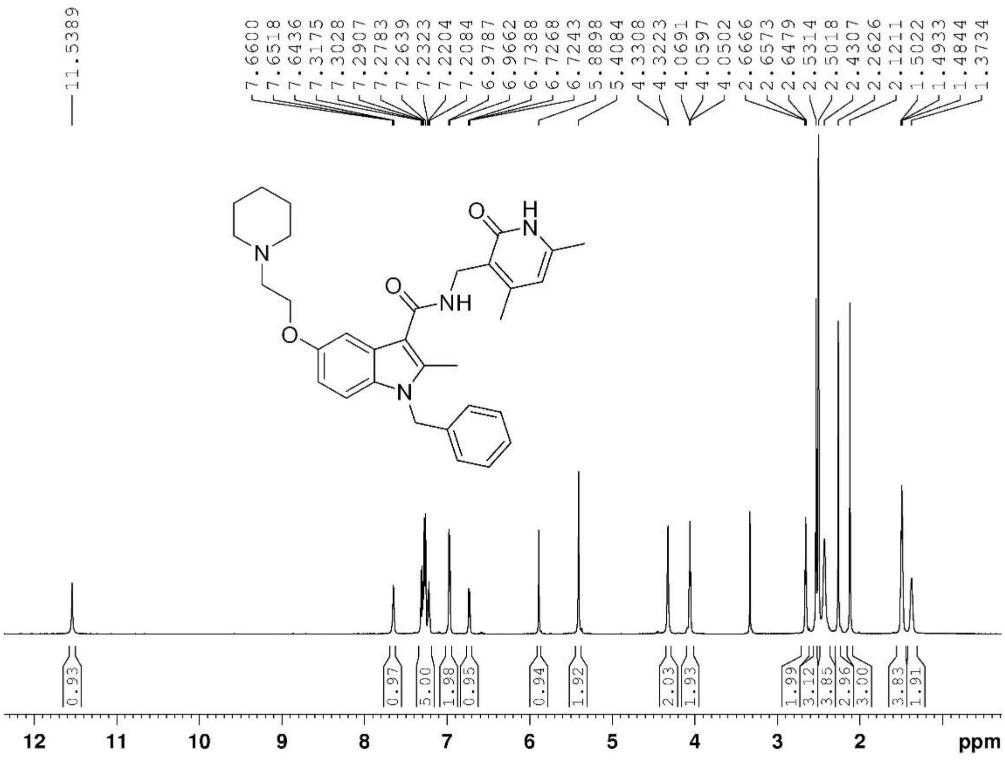

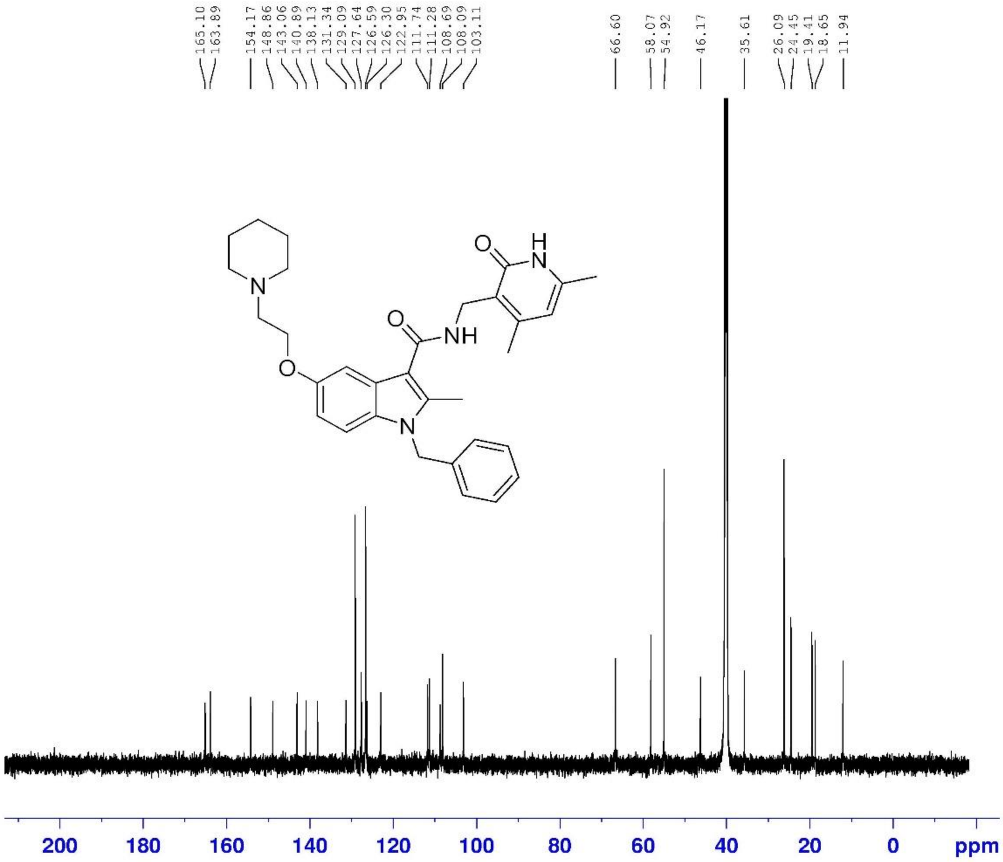

Spectrum of L-05

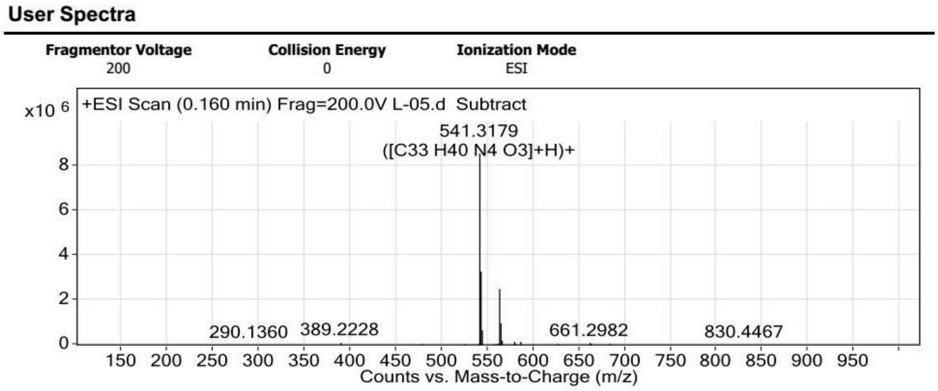

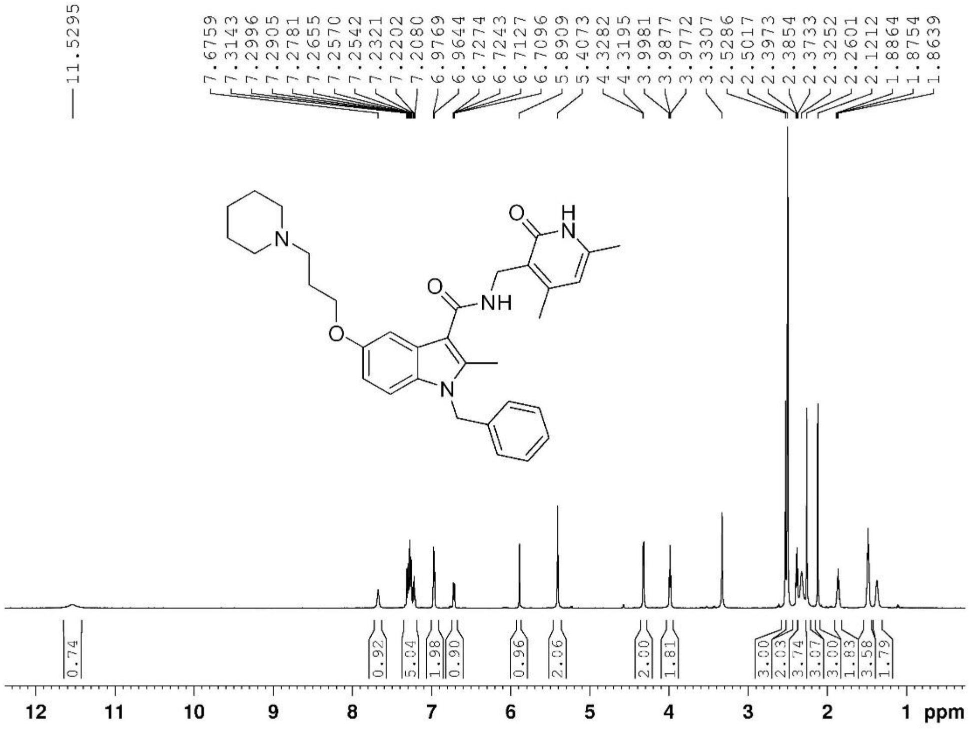
11.5295
7.6759
7.3143
7.2996
7.2905
7.2781
7.2655
7.2570
7.2542
7.2321
7.2202
7.2080
6.9769
6.9644
6.7274
6.7243
6.7127
6.7096
5.8909
5.4073
4.3282
4.3195
3.9981
3.9877
3.9772
3.3307
2.5286
2.5017
2.3973
2.3854
2.3733
2.3252
2.2601
2.1212
1.8864
1.8754
1.8639
O
H
N
N
O
NH
O
N
0.74
0.92
5.04
1.98
0.90
0.96
2.06
2.00
1.81
3.00
2.03
3.74
3.07
3.00
1.83
3.58
1.79
12
11
10
9
8
7
6
5
4
3
2
1
ppm

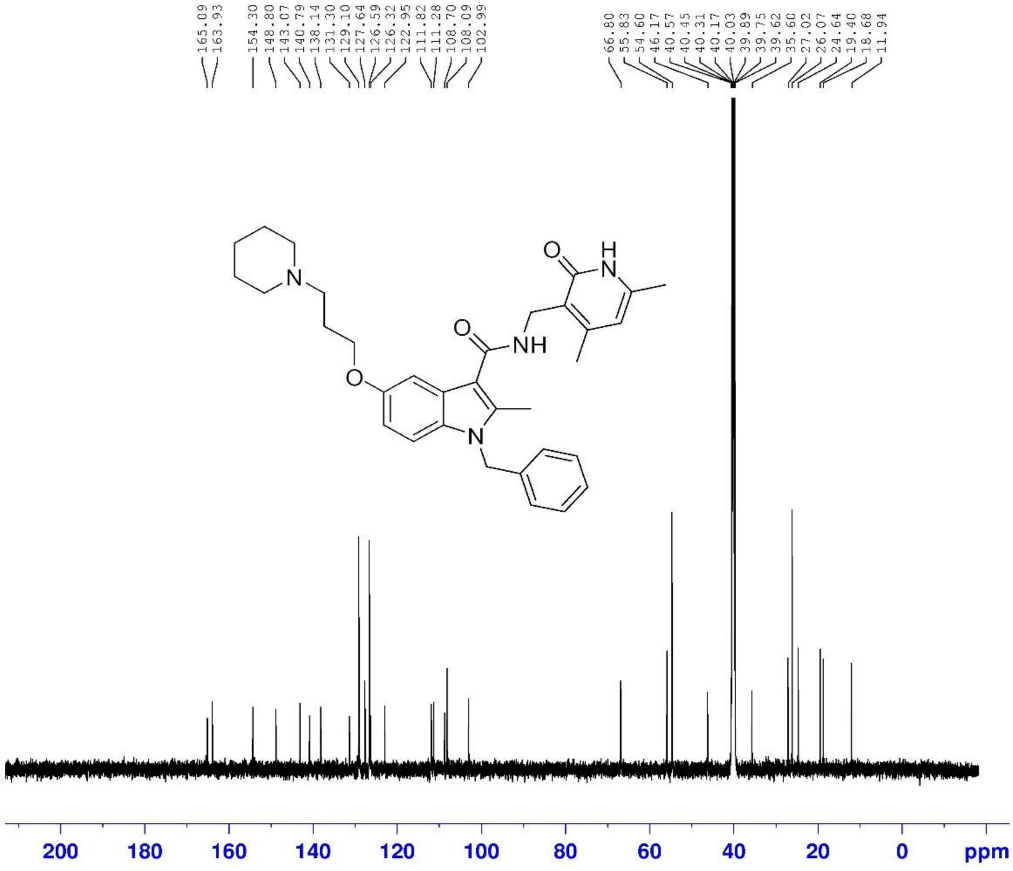
165.09
163.93
154.30
148.80
143.07
140.79
138.14
131.30
129.10
127.64
126.59
126.32
122.95
111.82
111.28
108.70
108.09
102.99
66.80
55.83
54.60
46.17
40.57
40.45
40.31
40.17
40.03
39.89
39.75
39.62
35.60
27.02
26.07
24.64
19.40
18.68
11.94
O
H
N
N
O
NH
O
N
200
180
160
140
120
100
80
60
40
20
0
ppm

Spectrum of L-06

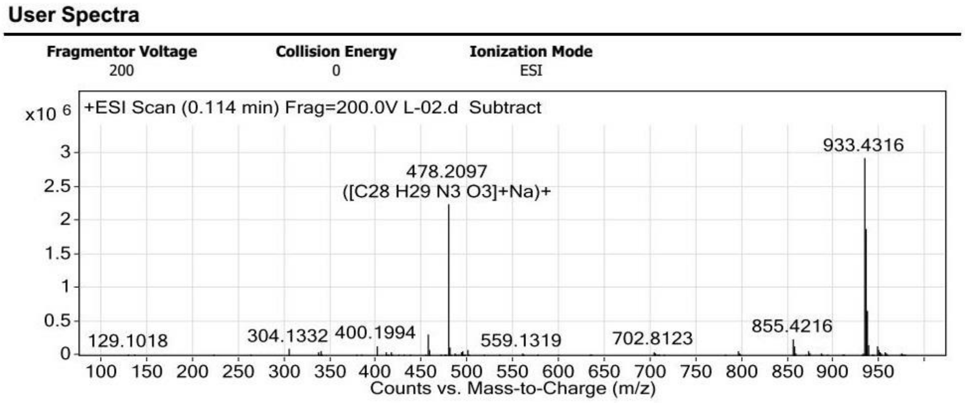

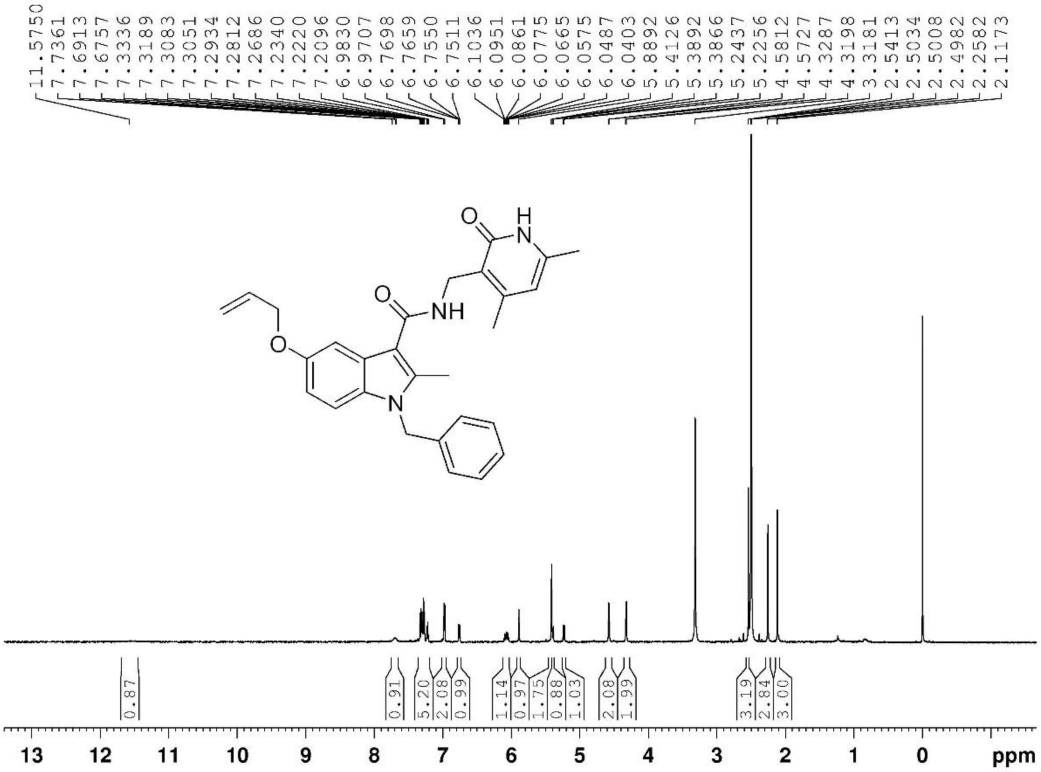

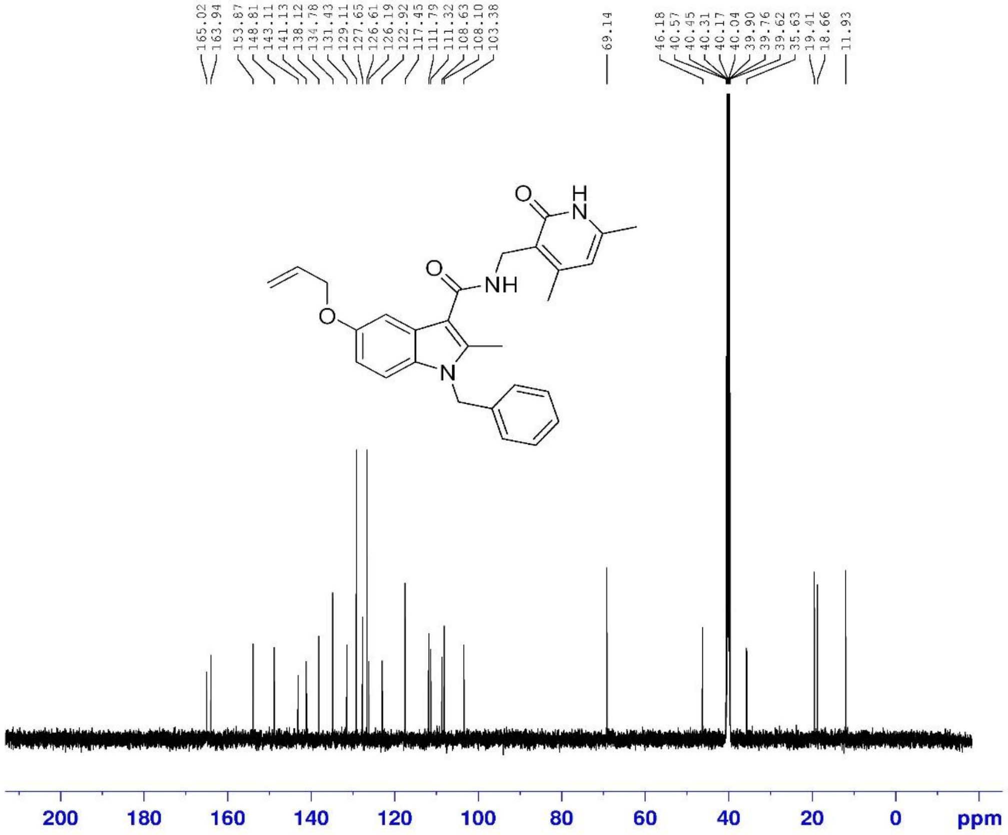

Spectrum of L-07

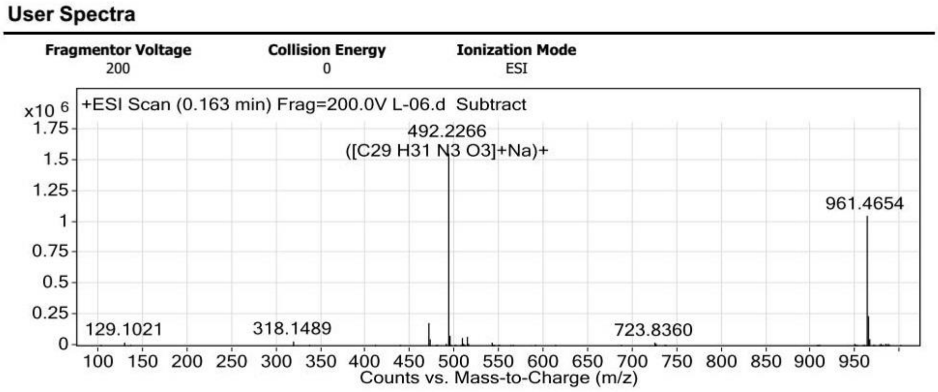

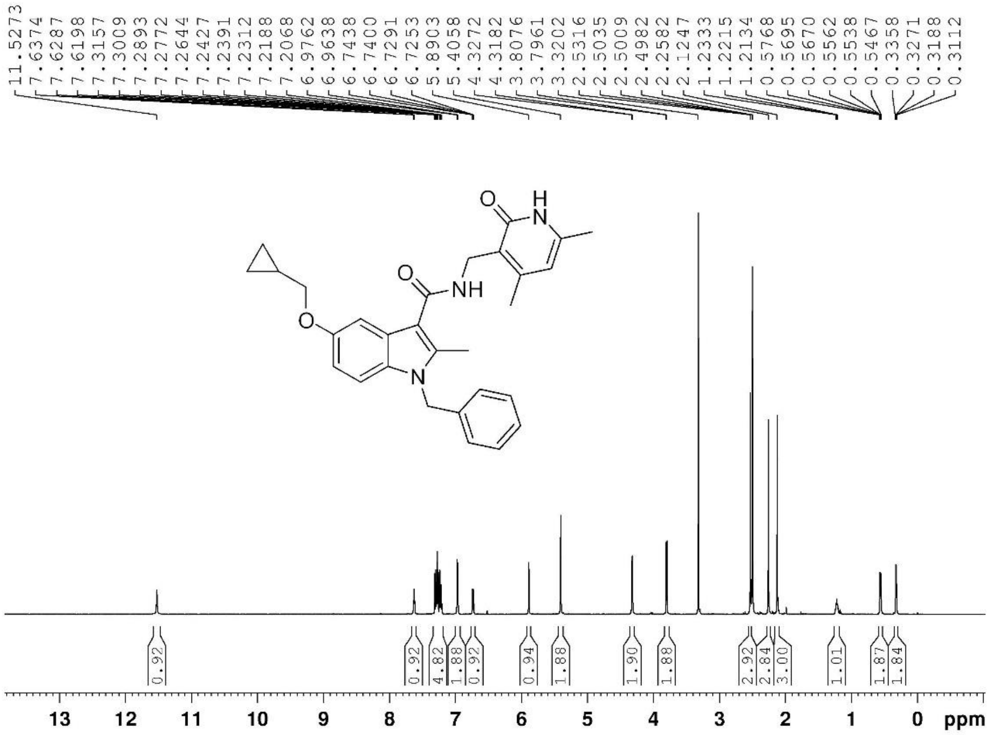

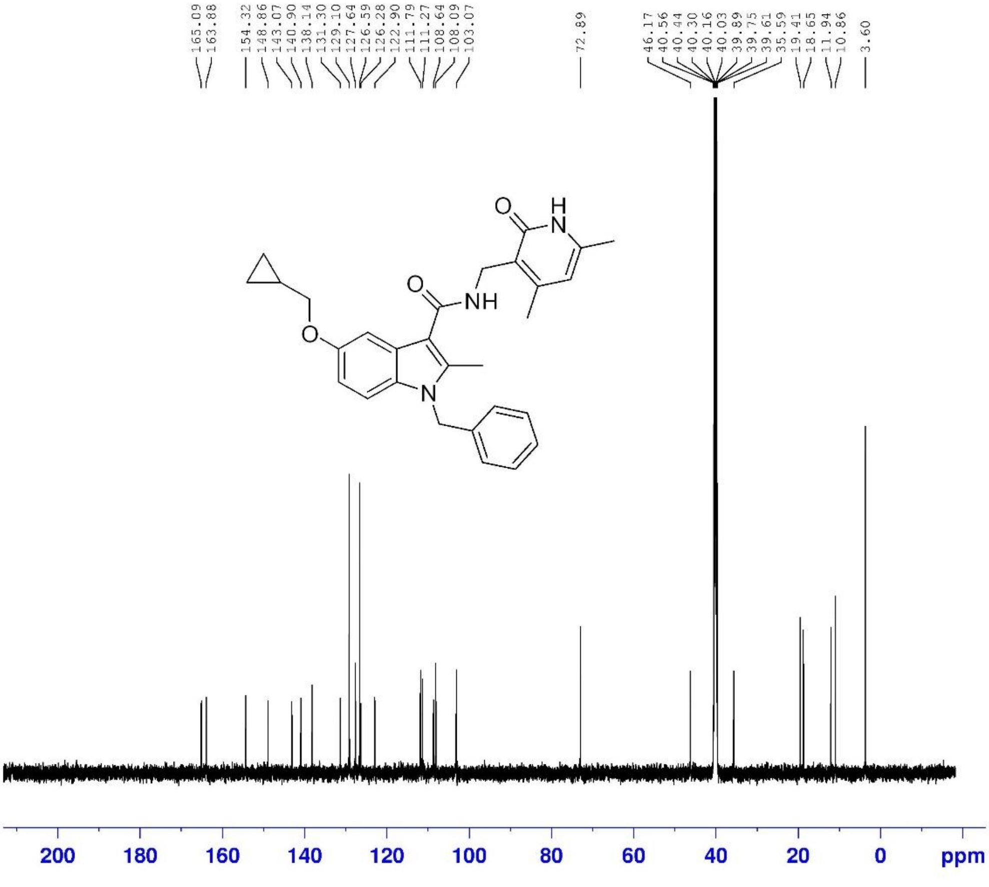

Spectrum of L-08

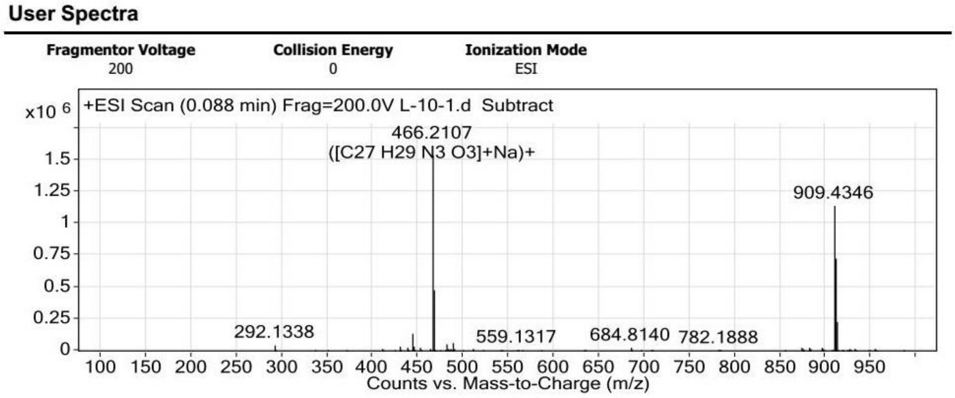

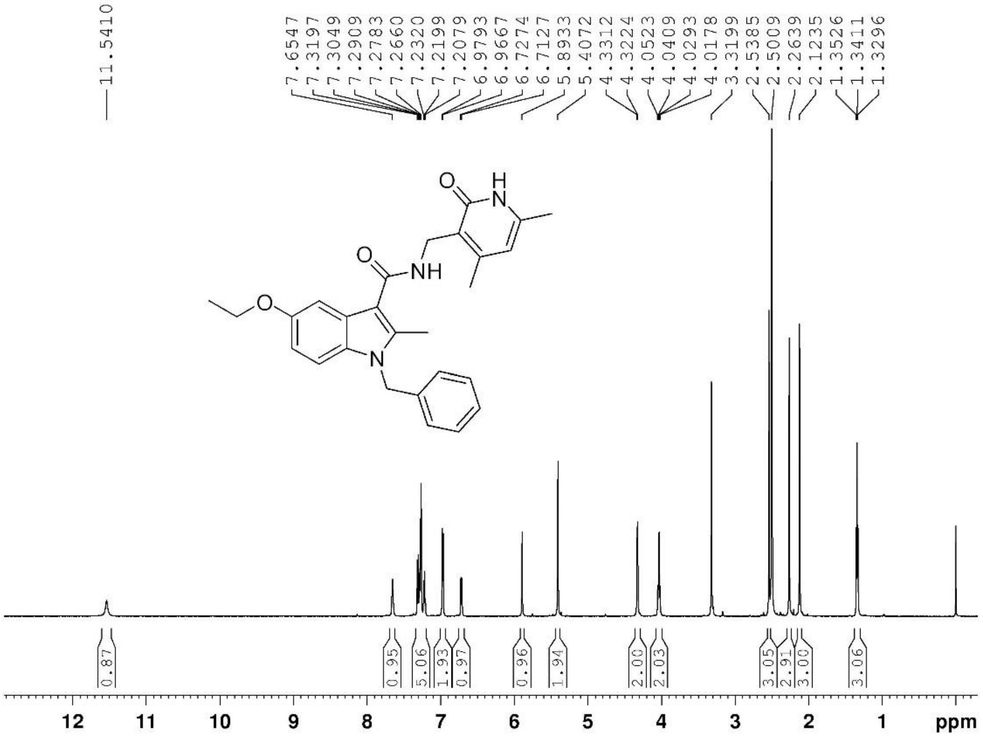

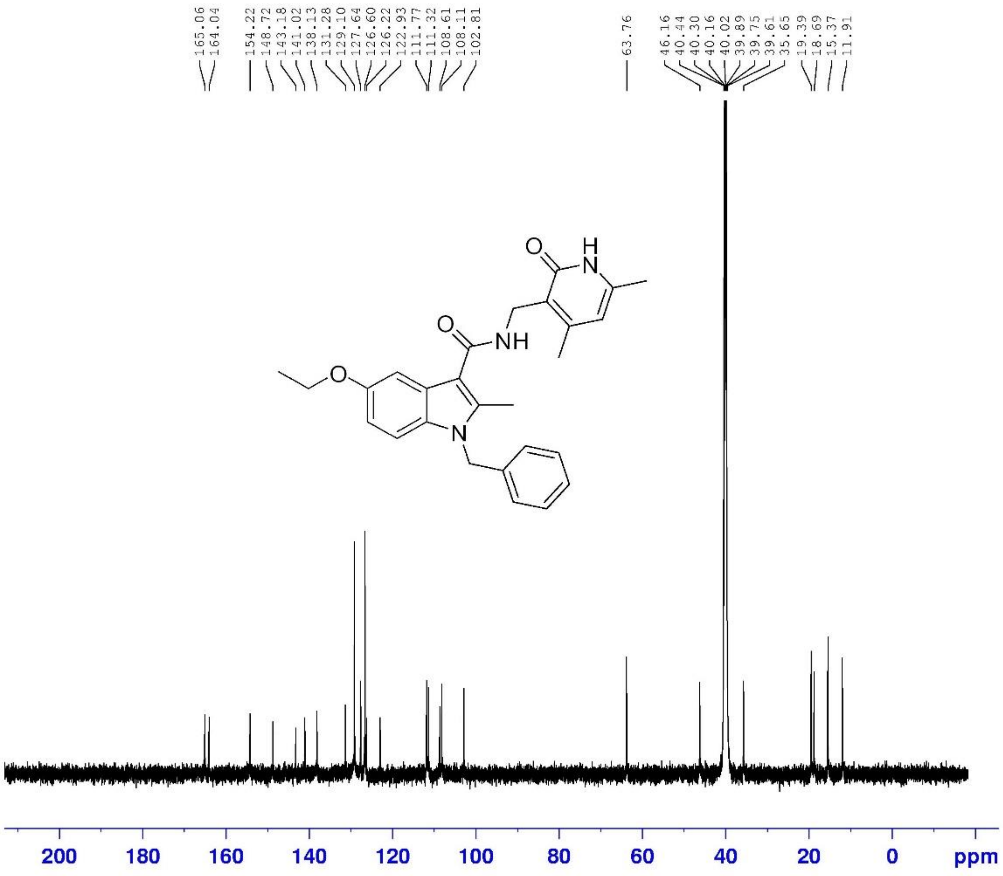

Spectrum of L-09

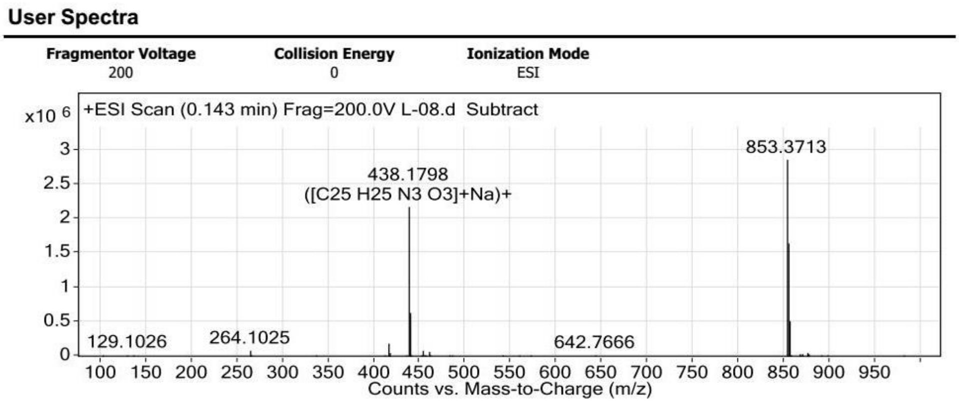

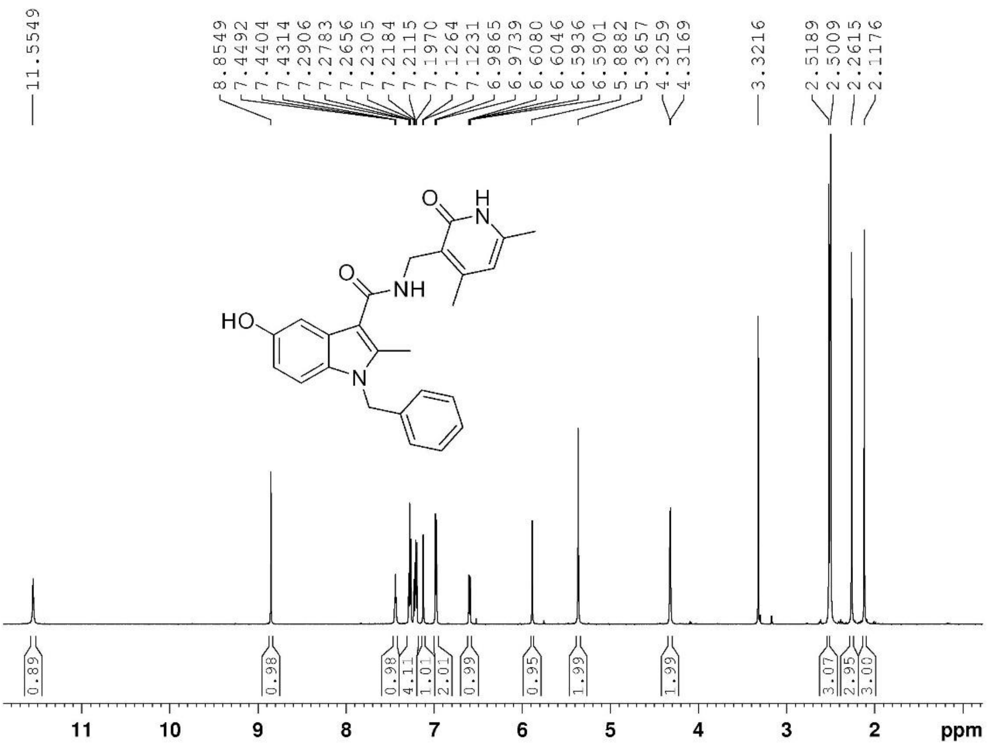
11.5549
8.8549
7.4492
7.4404
7.4314
7.2906
7.2783
7.2656
7.2305
7.2184
7.2115
7.1970
7.1264
7.1231
6.9865
6.9739
6.6080
6.6046
6.5936
6.5901
5.8882
5.3657
4.3259
4.3169
3.3216
2.5189
2.5009
2.2615
2.1176
O
H
N
NH
HO
N
0.89
0.98
0.98
4.11
1.01
2.01
0.99
0.95
1.99
1.99
3.07
2.95
3.00
11
10
9
8
7
6
5
4
3
2
ppm

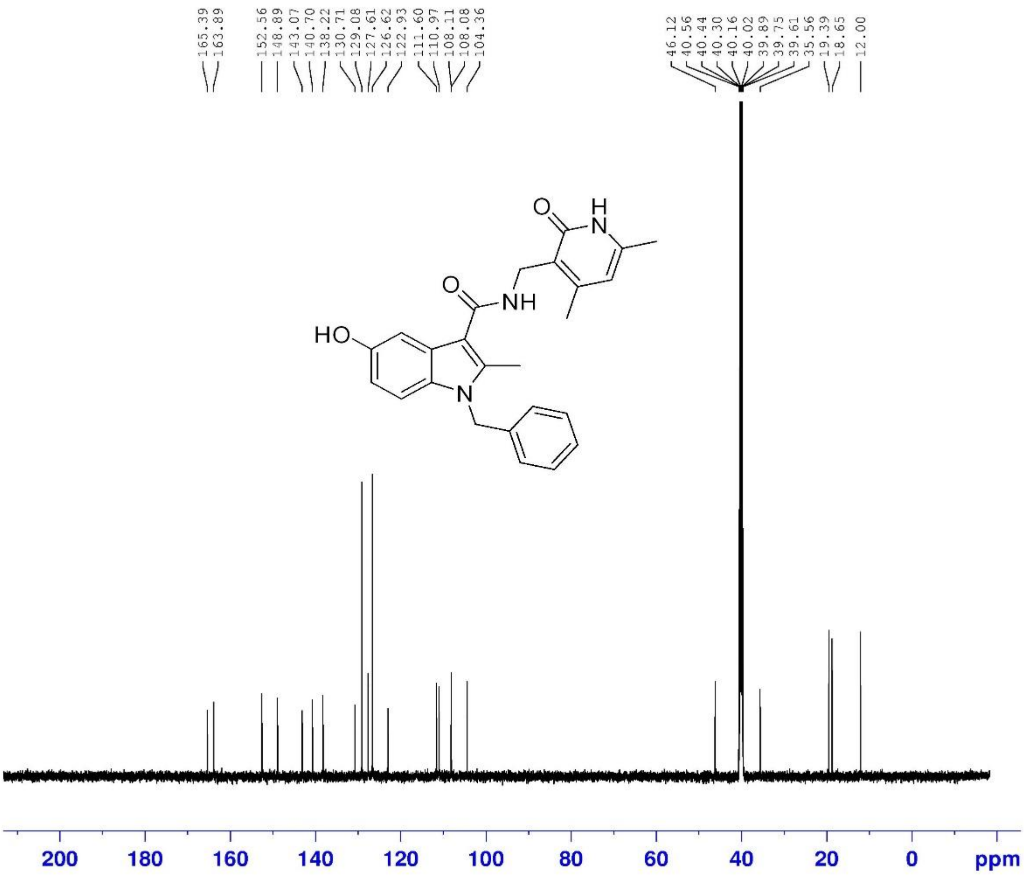
165.39
163.89
152.56
148.89
143.07
140.70
138.22
130.71
129.08
127.61
126.62
122.93
111.60
110.97
108.11
108.08
104.36
46.12
40.56
40.44
40.30
40.16
40.02
39.89
39.75
39.61
35.56
19.39
18.65
12.00
O
H
N
NH
HO
N
200
180
160
140
120
100
80
60
40
20
0
ppm

Spectrum of L-10

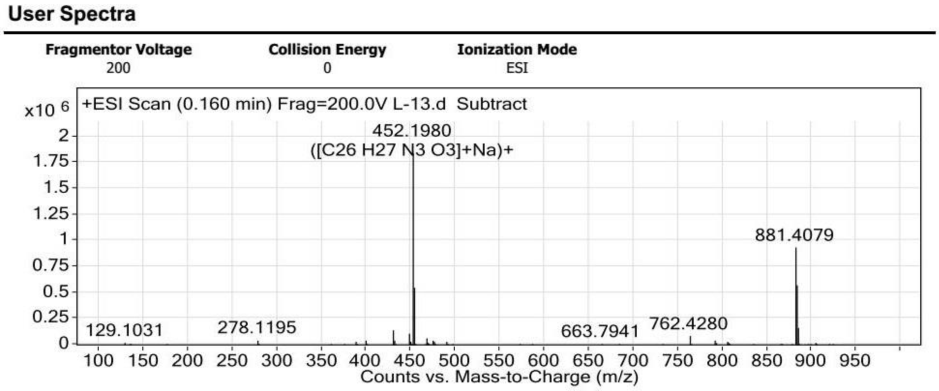

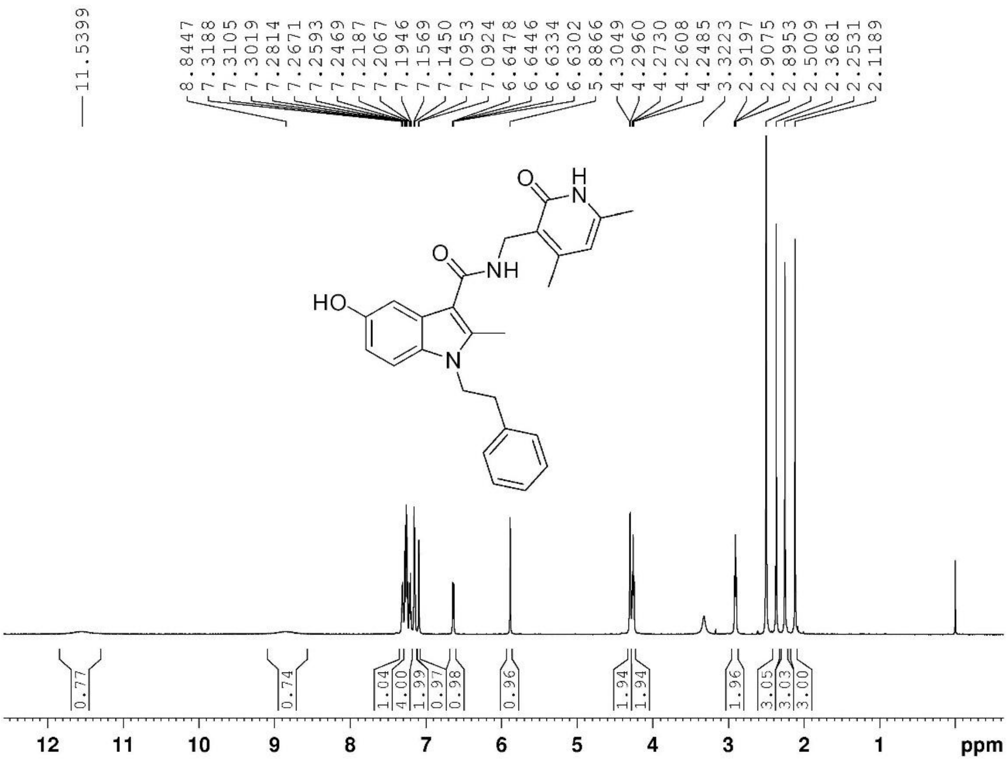

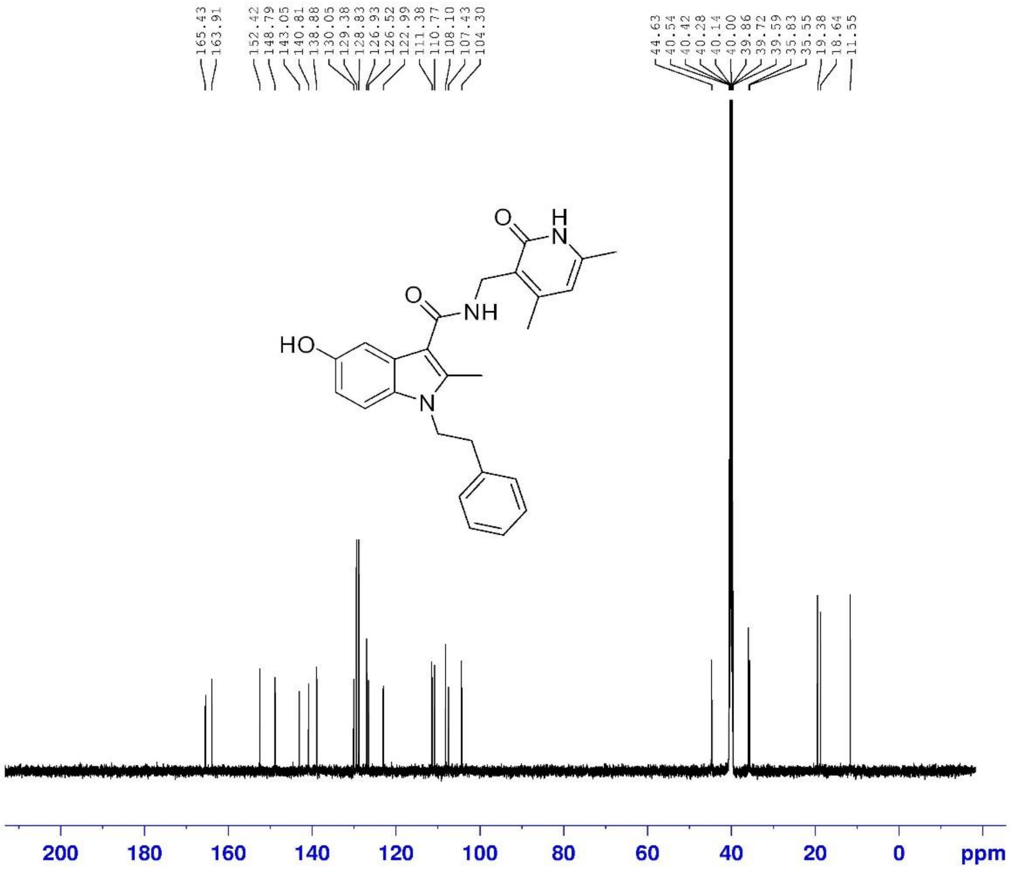

Spectrum of L-11

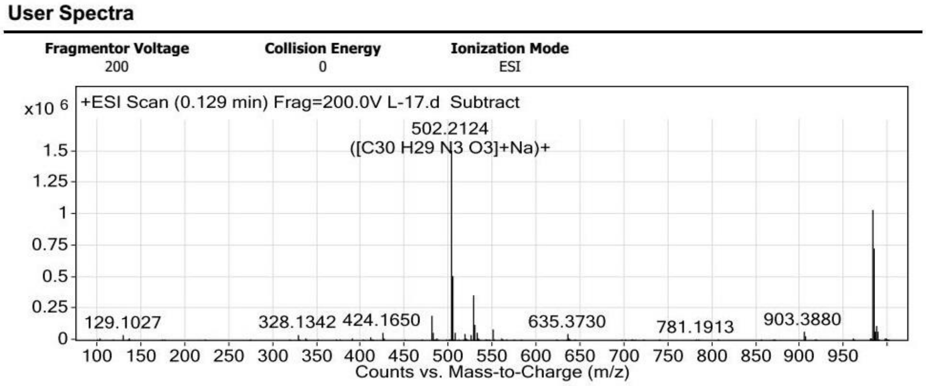

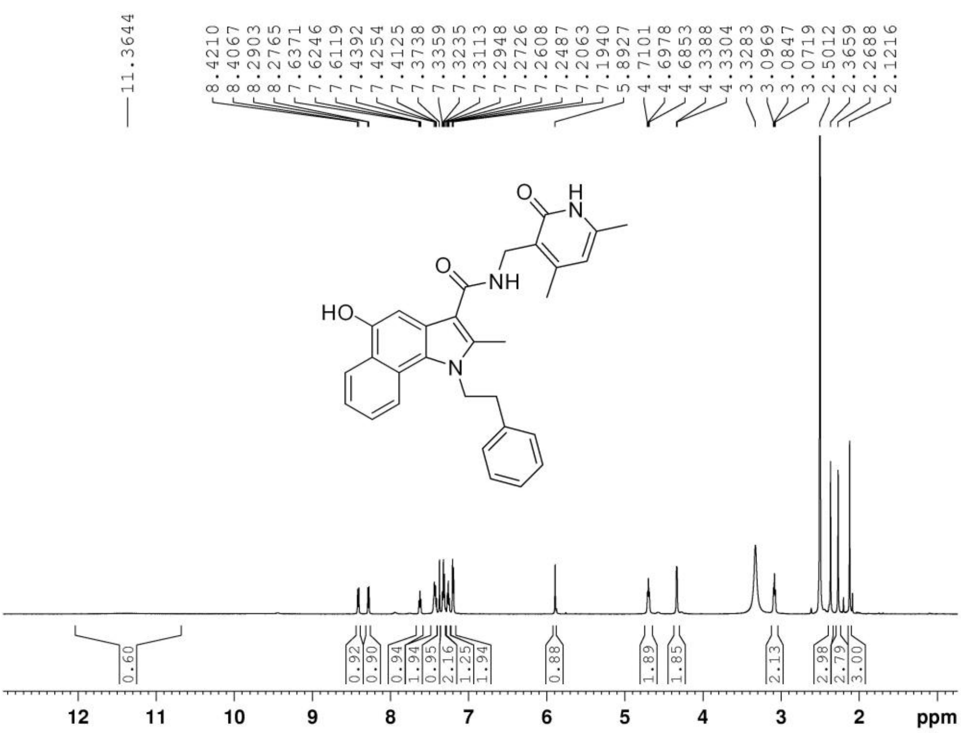
11.3644
8.4210 8.4067 8.2903 8.2765 7.6371 7.6246 7.6119 7.4392 7.4254 7.4125 7.3738 7.3359 7.3235 7.3113 7.2948 7.2726 7.2608 7.2487 7.2063 7.1940 5.8927 4.7101 4.6978 4.6853 4.3388 4.3304 3.3283 3.0969 3.0847 3.0719 2.5012 2.3659 2.2688 2.1216
HO
N
O
NH
O
H
N
0.60 0.92 0.90 0.94 1.94 0.95 2.16 1.25 1.94 0.88 1.89 1.85 2.13 2.98 2.79 3.00
12 11 10 9 8 7 6 5 4 3 2 ppm

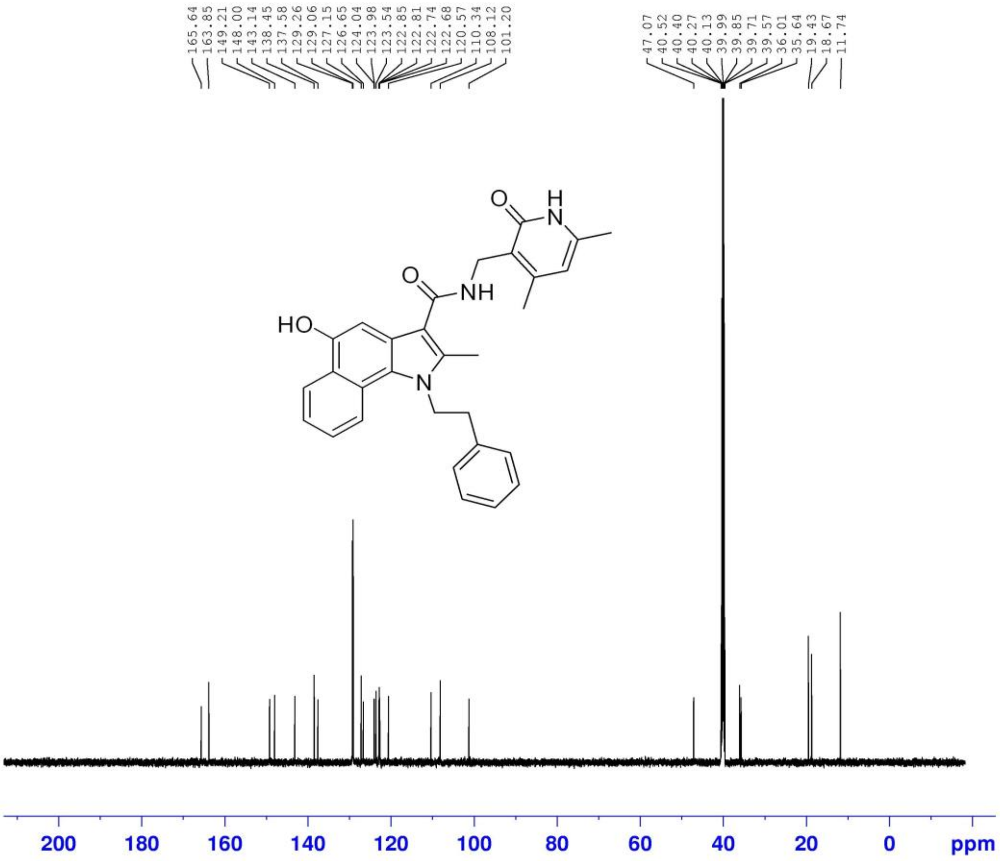
165.64 163.85 149.21 148.00 143.14 138.45 137.58 129.26 129.06 127.15 126.65 124.04 123.98 123.54 122.85 122.81 122.74 122.68 120.57 110.34 108.12 101.20
47.07 40.52 40.40 40.27 40.13 39.99 39.85 39.71 39.57 36.01 35.64 19.43 18.67 11.74
HO
N
O
NH
O
H
N
200 180 160 140 120 100 80 60 40 20 0 ppm

Spectrum of L-12

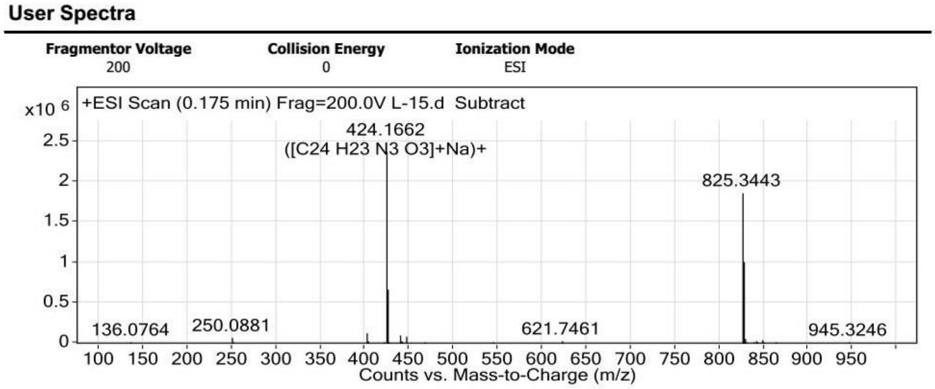

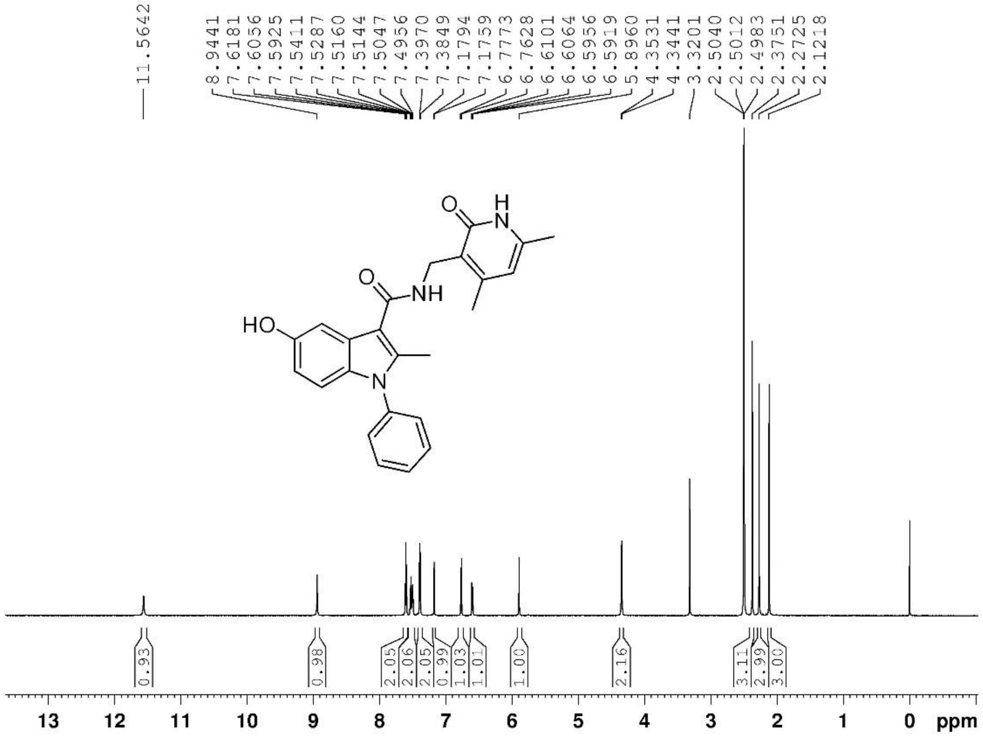

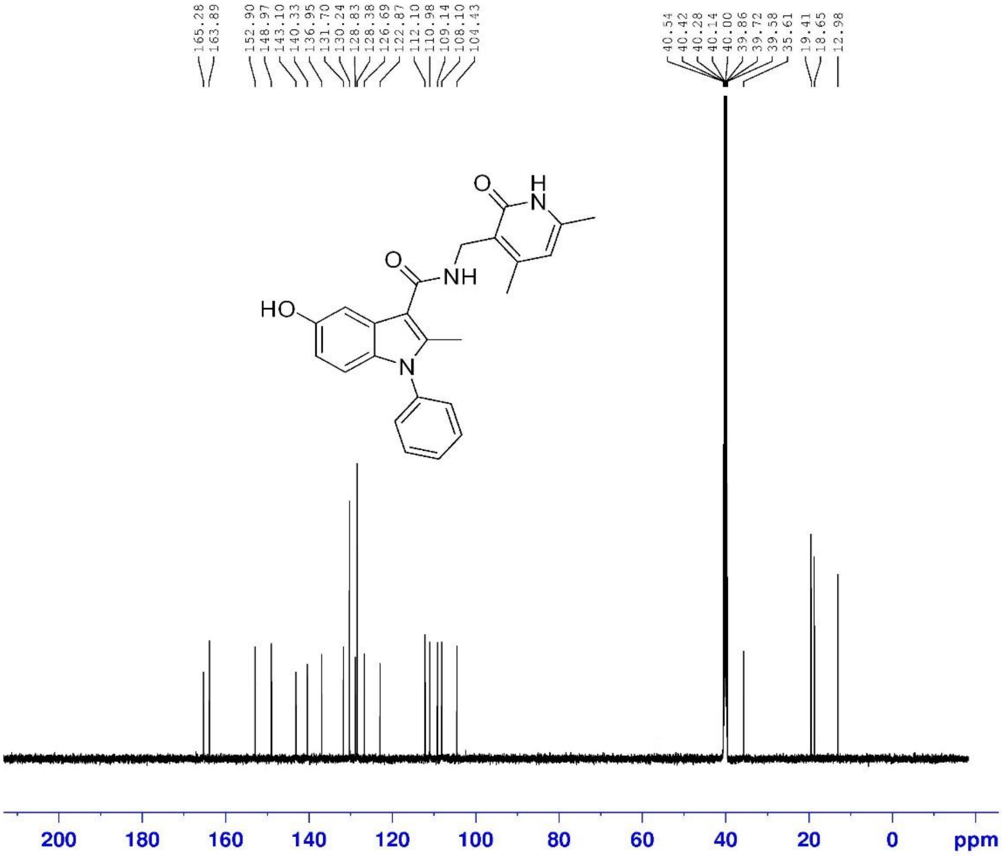

Spectrum of L-13

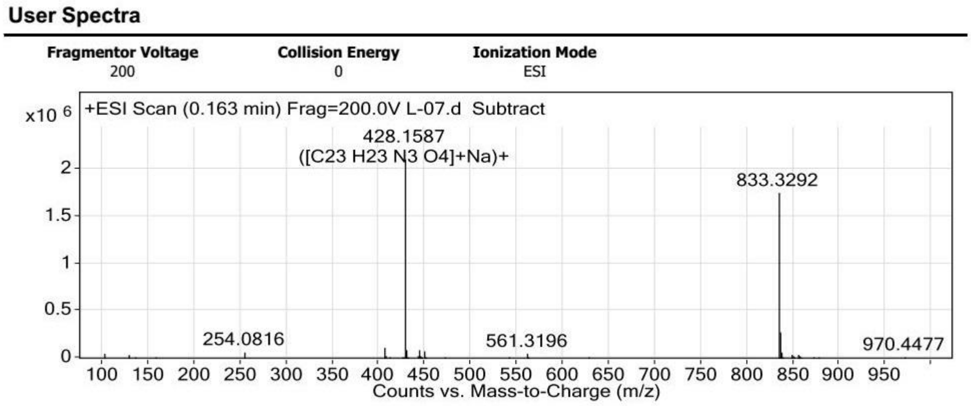

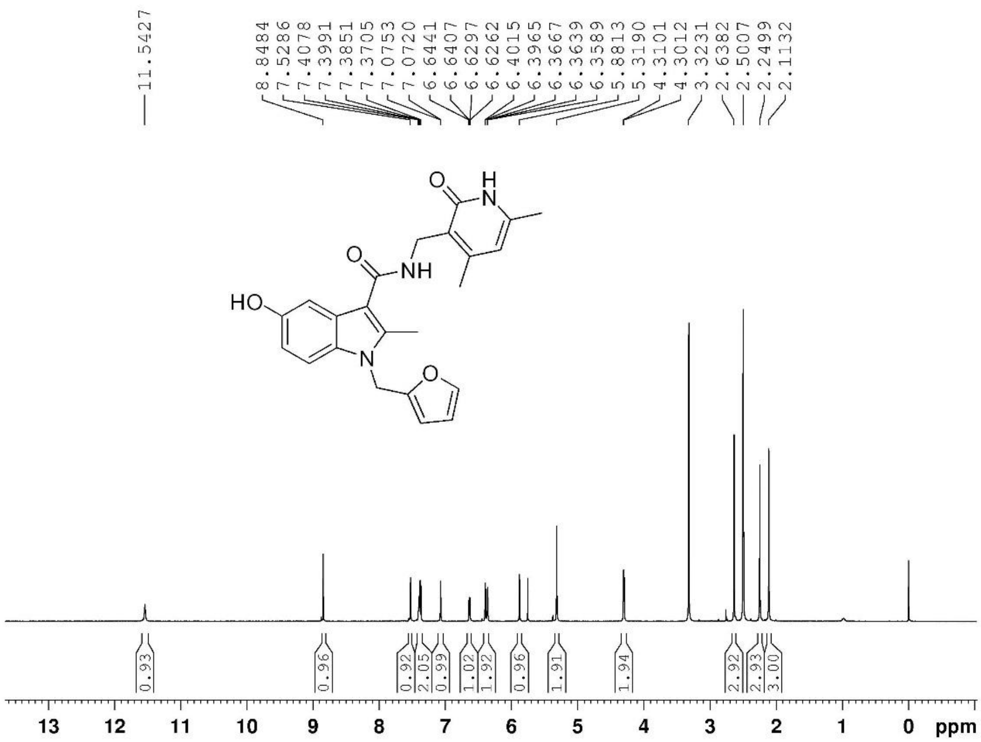

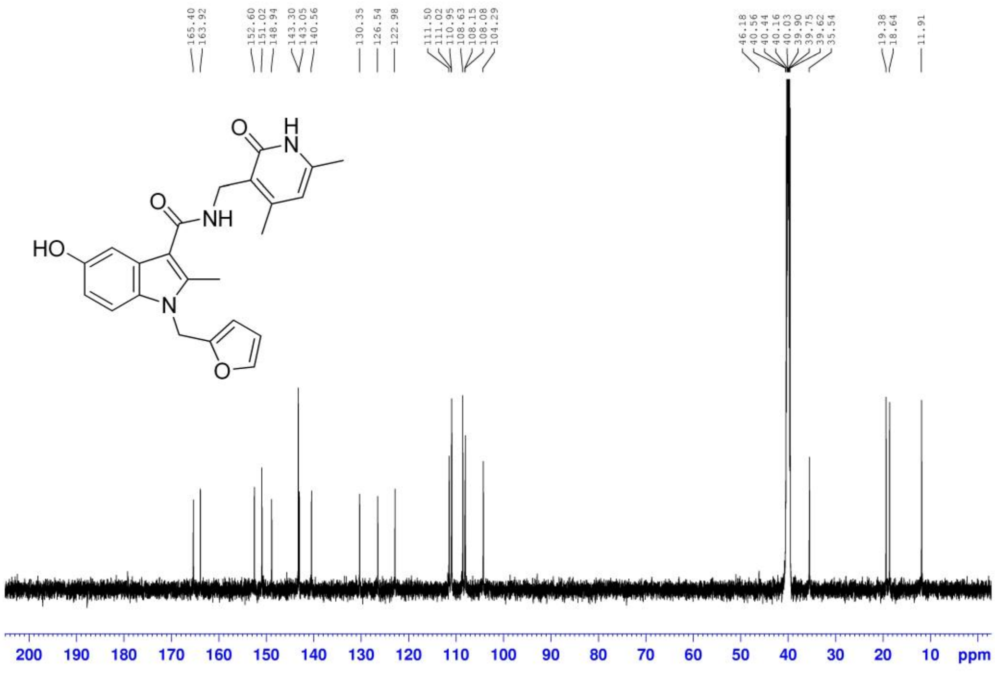

Spectrum of L-14

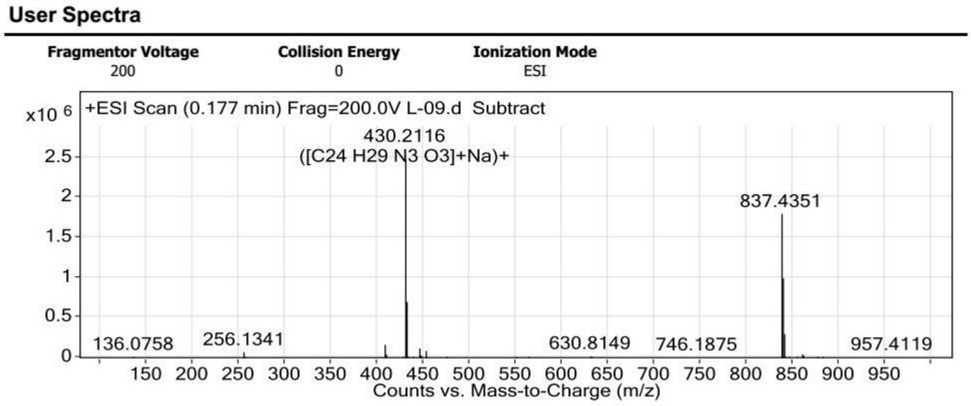

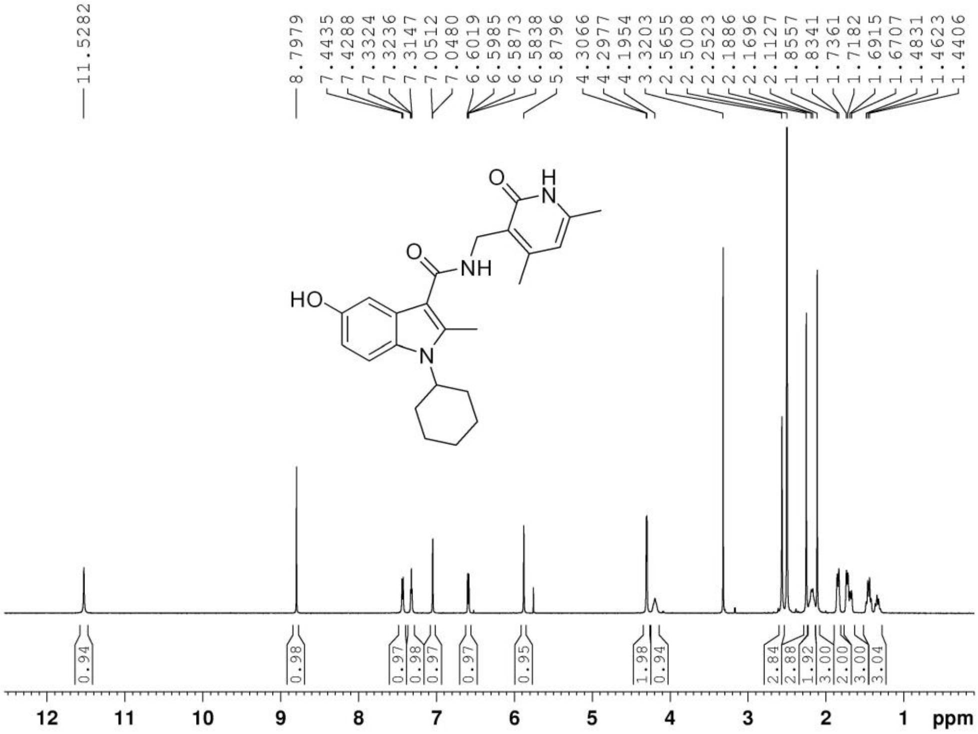

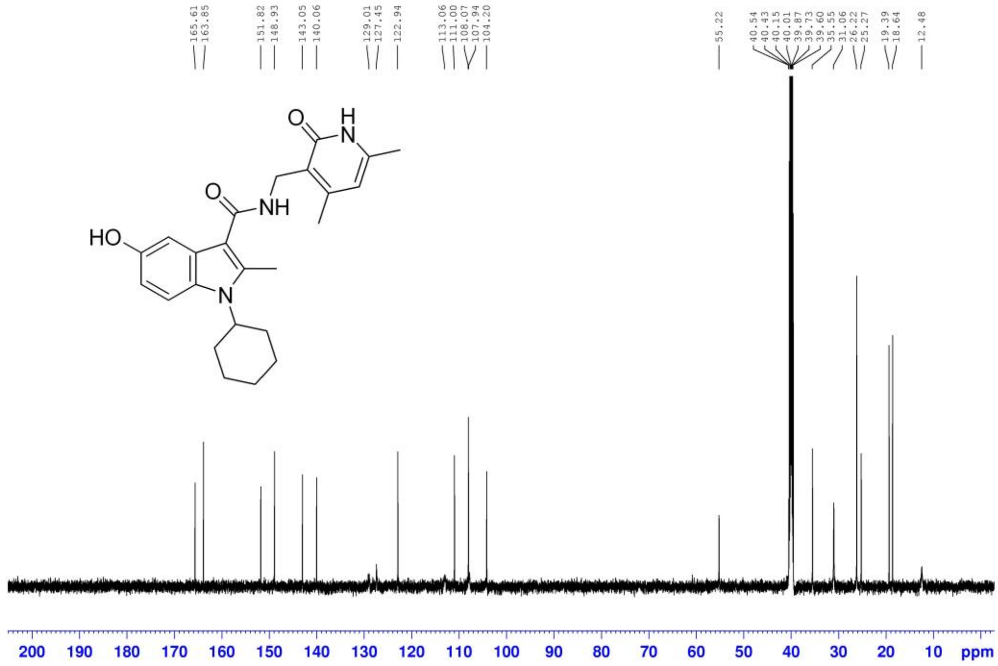

Spectrum of L-15

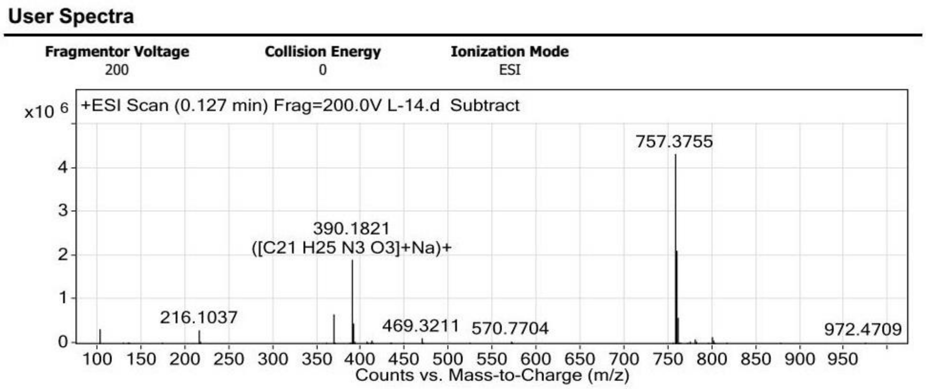

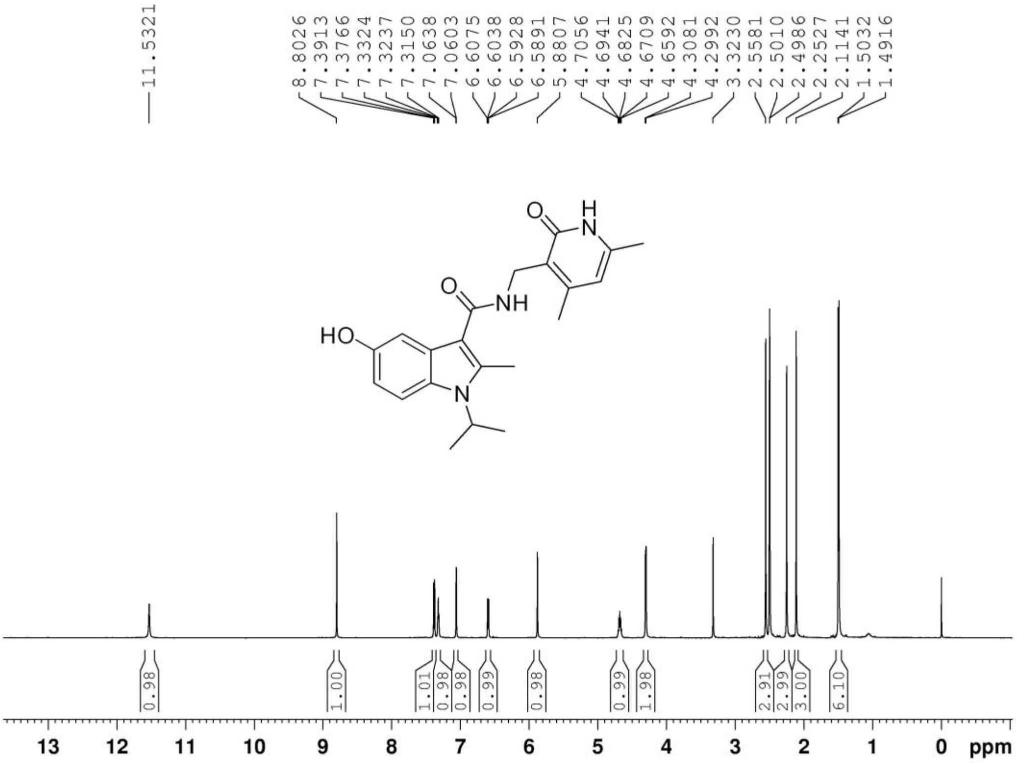
11.5321
8.8026 7.3913 7.3766 7.3324 7.3237 7.3150 7.0638 7.0603 6.6075 6.6038 6.5928 6.5891 5.8807 4.7056 4.6941 4.6825 4.6709 4.6592 4.3081 4.2992 3.3230 2.5581 2.5010 2.4986 2.2527 2.1141 1.5032 1.4916
O
H
N
O
NH
HO
N
0.98
1.00
1.01 0.98 0.98
0.99
0.98
0.99 1.98
2.91 2.99 3.00
6.10
13 12 11 10 9 8 7 6 5 4 3 2 1 0 ppm

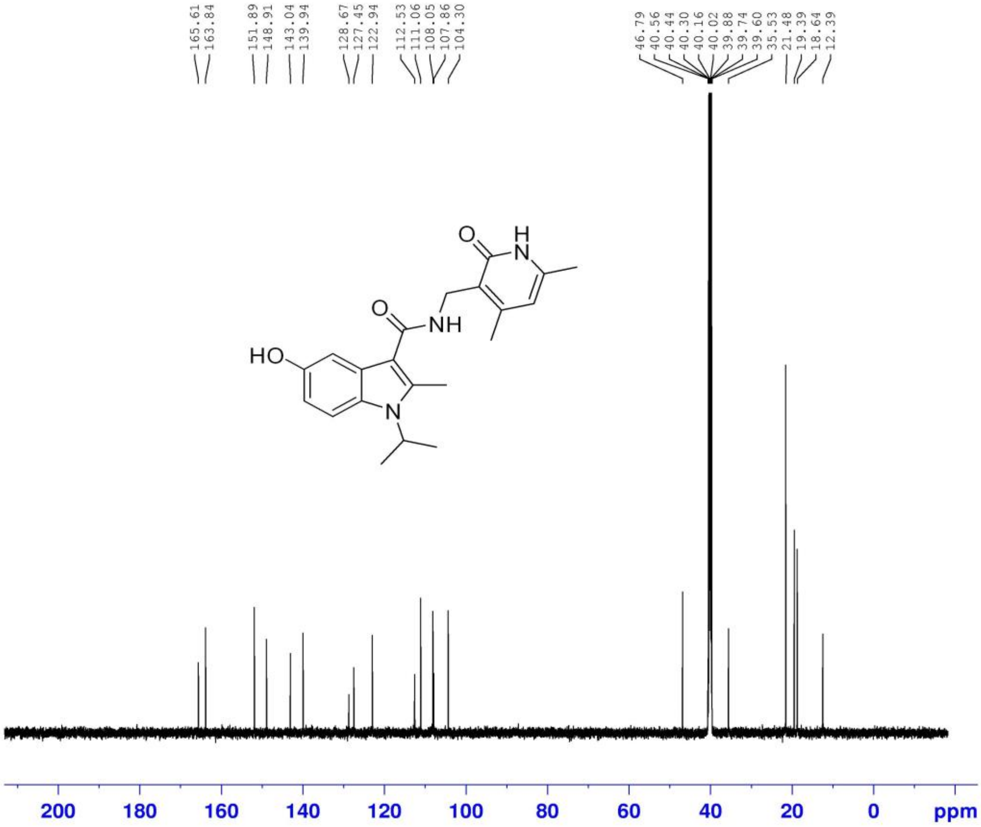
165.61 163.84
151.89 148.91 143.04 139.94
128.67 127.45 122.94
112.53 111.06 108.05 107.86 104.30
46.79 40.56 40.44 40.30 40.16 40.02 39.88 39.74 39.60 35.53 21.48 19.39 18.64 12.39
O
H
N
O
NH
HO
N
200 180 160 140 120 100 80 60 40 20 0 ppm

Spectrum of L-16

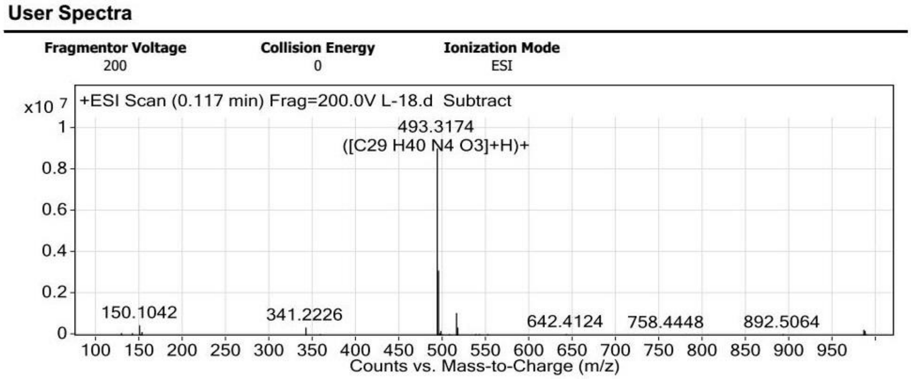

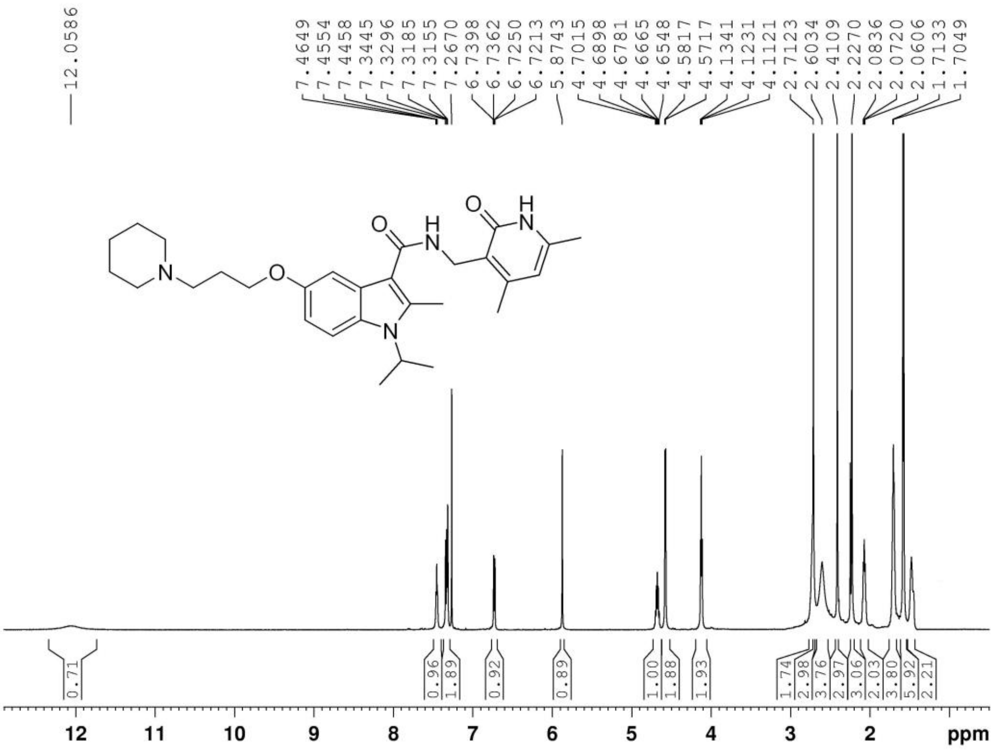

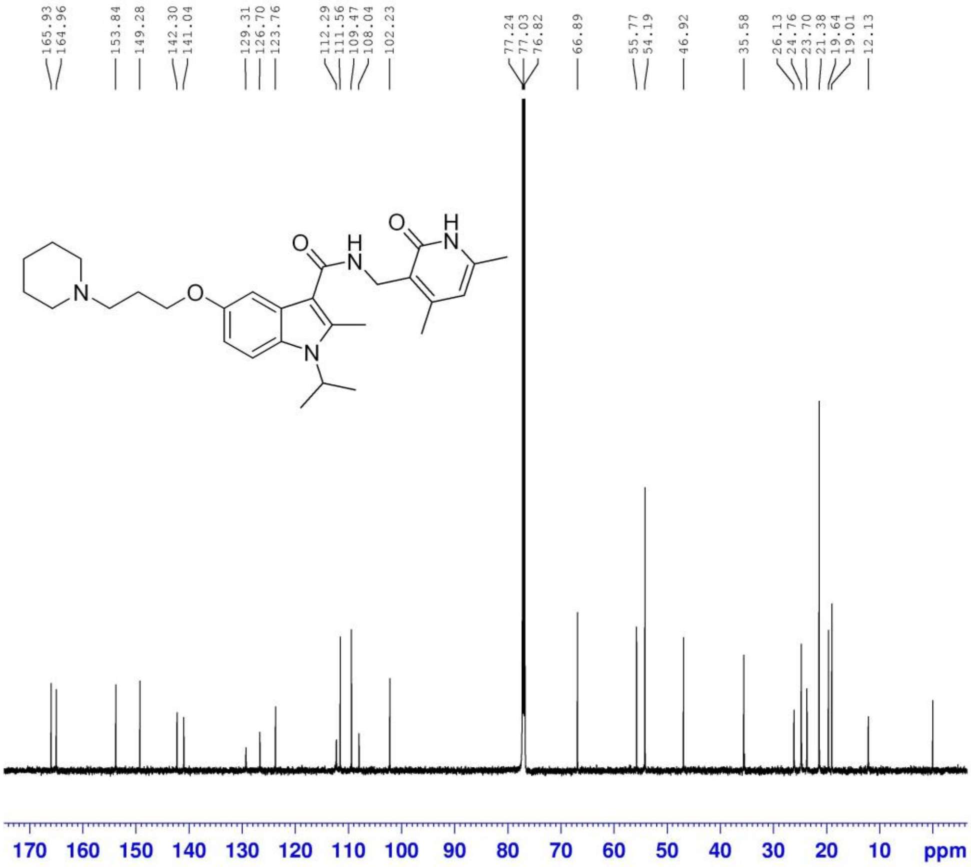

Spectrum of L-17

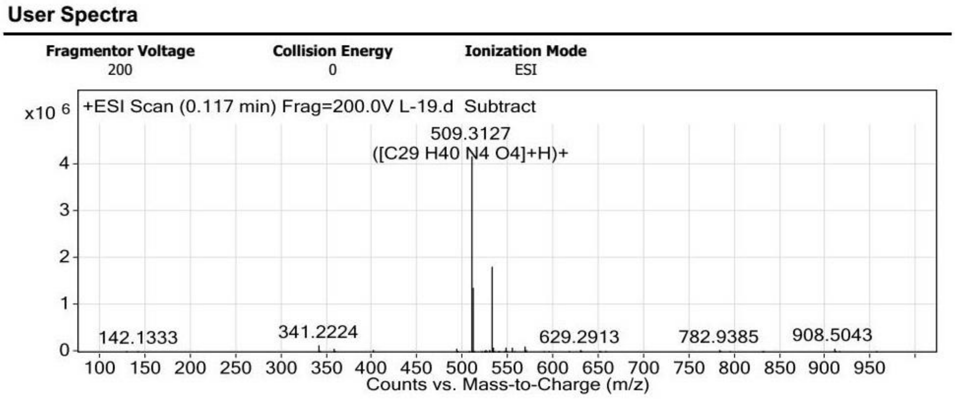

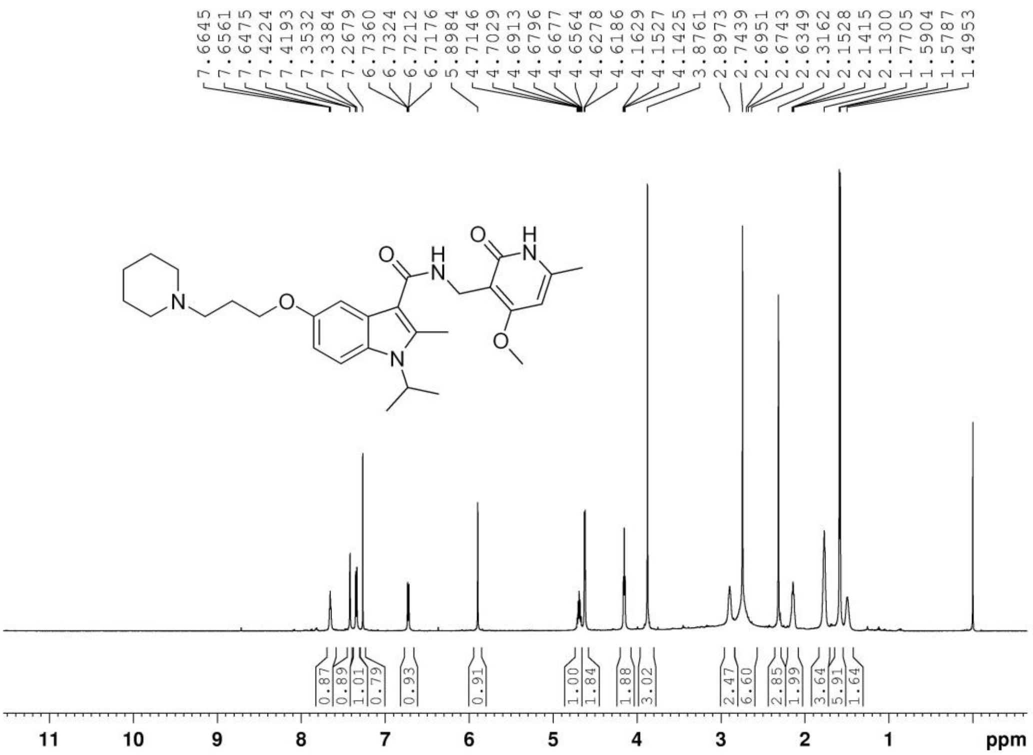
7.6645 7.6561 7.6475 7.4224 7.4193 7.3532 7.3384 7.2679 6.7360 6.7324 6.7212 6.7176 5.8984 4.7146 4.7029 4.6913 4.6796 4.6677 4.6564 4.6278 4.6186 4.1629 4.1527 4.1425 3.8761 2.8973 2.7439 2.6951 2.6743 2.6349 2.3162 2.1528 2.1415 2.1300 1.7705 1.5904 1.5787 1.4953
O
H
N
N
O
O
H
N
O
N
0.87 0.89 1.01 0.79 0.93 0.91 1.00 1.84 1.88 3.02 2.47 6.60 2.85 1.99 3.64 5.91 1.64
11 10 9 8 7 6 5 4 3 2 1 ppm

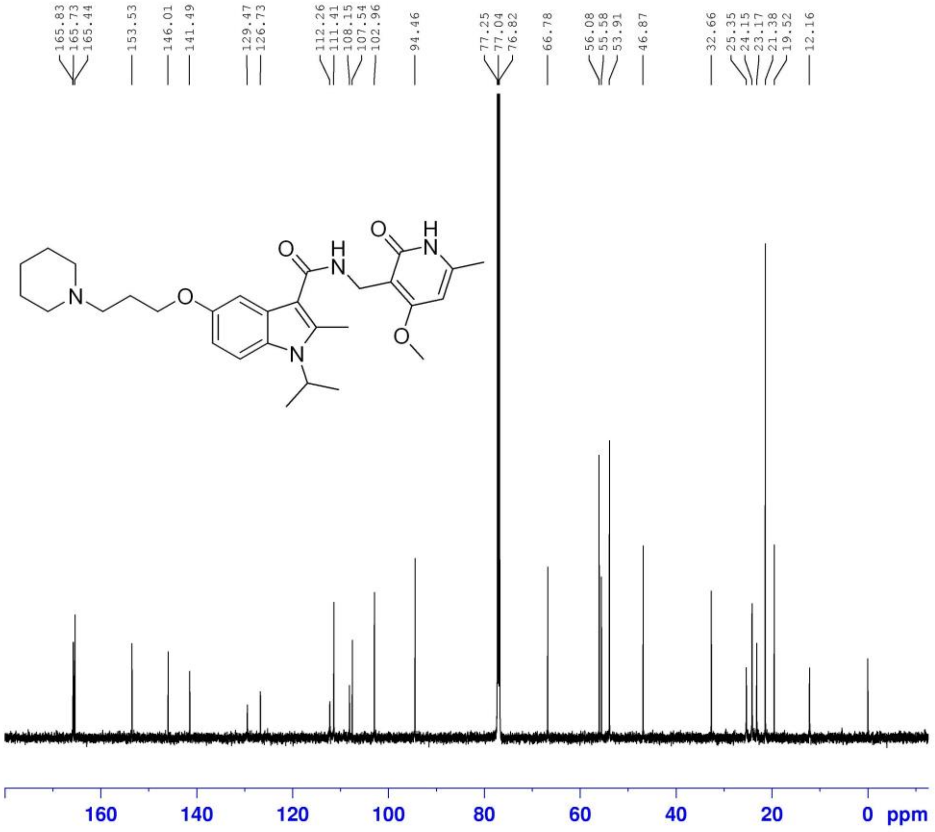
165.83 165.73 165.44 153.53 146.01 141.49 129.47 126.73 112.26 111.41 108.15 107.54 102.96 94.46 77.25 77.04 76.82 66.78 56.08 55.58 53.91 46.87 32.66 25.35 24.15 23.17 21.38 19.52 12.16
O
H
N
N
O
O
H
N
O
N
160 140 120 100 80 60 40 20 0 ppm

Spectrum of L-18

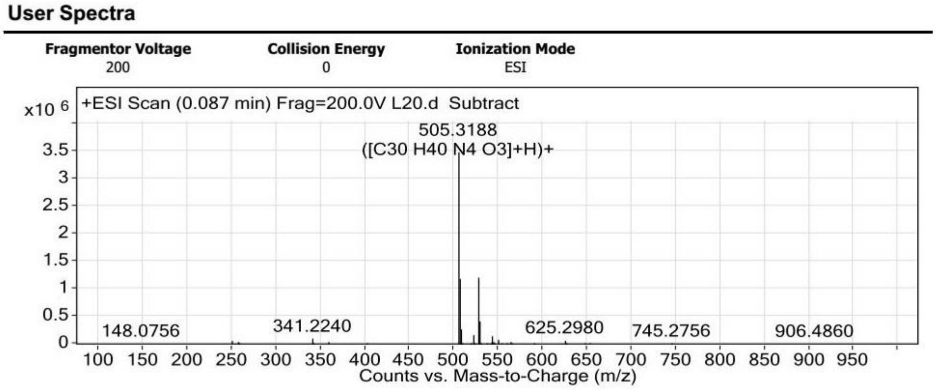

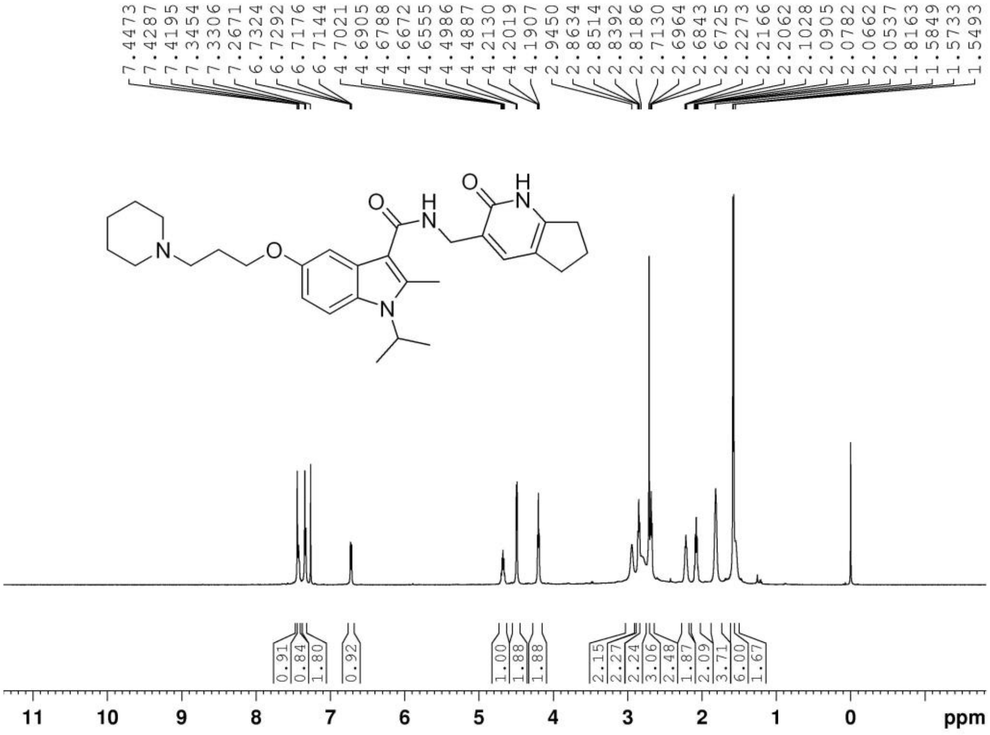

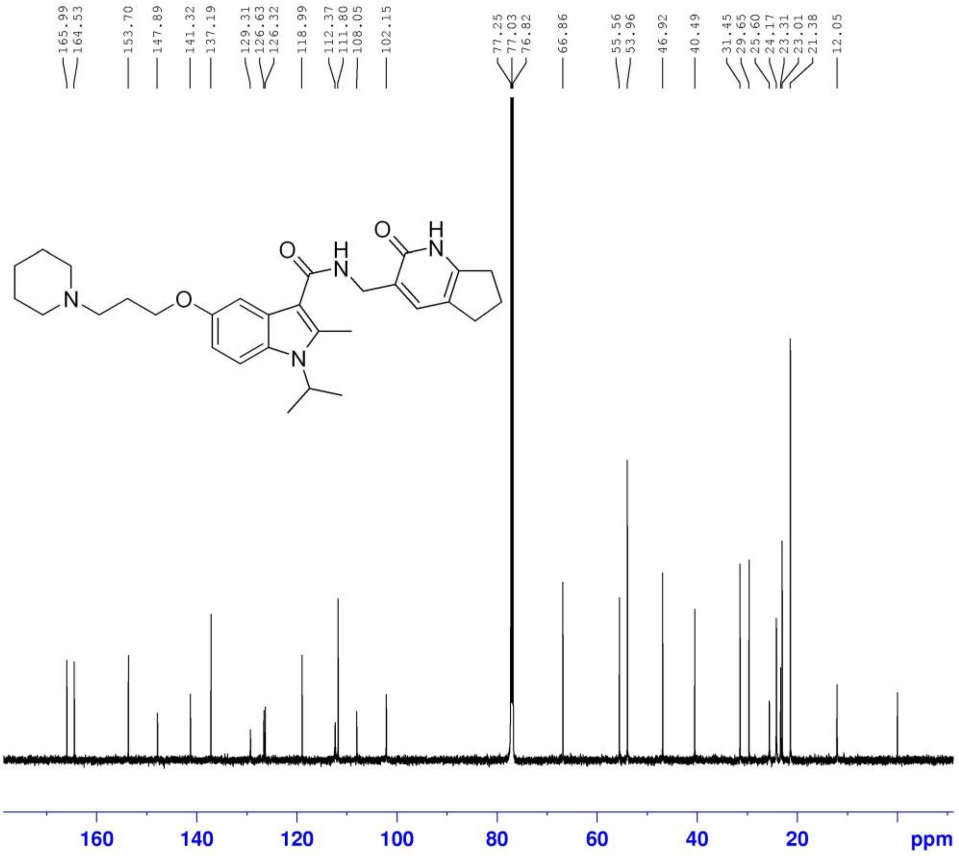

Spectrum of L-19

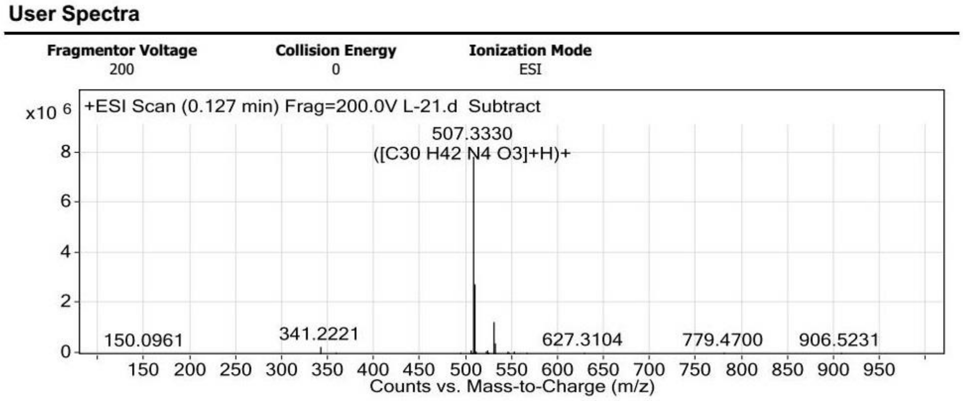

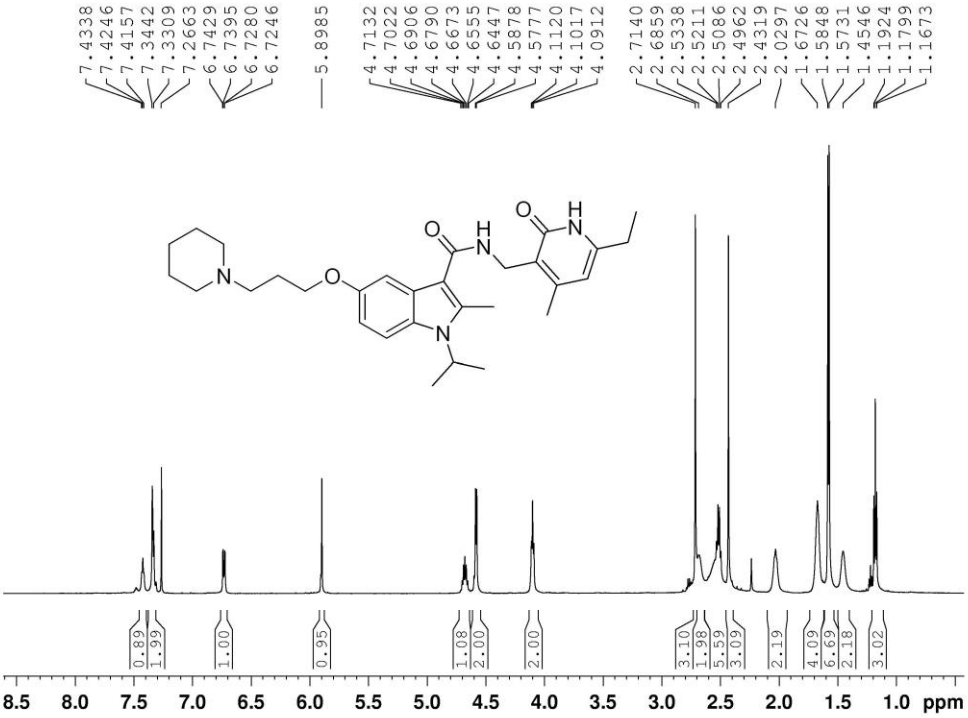
7.4338
7.4246
7.4157
7.3442
7.3309
7.2663
6.7429
6.7395
6.7280
6.7246
5.8985
4.7132
4.7022
4.6906
4.6790
4.6673
4.6555
4.6447
4.5878
4.5777
4.1120
4.1017
4.0912
2.7140
2.6859
2.5338
2.5211
2.5086
2.4962
2.4319
2.0297
1.6726
1.5848
1.5731
1.4546
1.1924
1.1799
1.1673
0.89
1.99
1.00
0.95
1.08
2.00
2.00
3.10
1.98
5.59
3.09
2.19
4.09
6.69
2.18
3.02
8.5 8.0 7.5 7.0 6.5 6.0 5.5 5.0 4.5 4.0 3.5 3.0 2.5 2.0 1.5 1.0 ppm

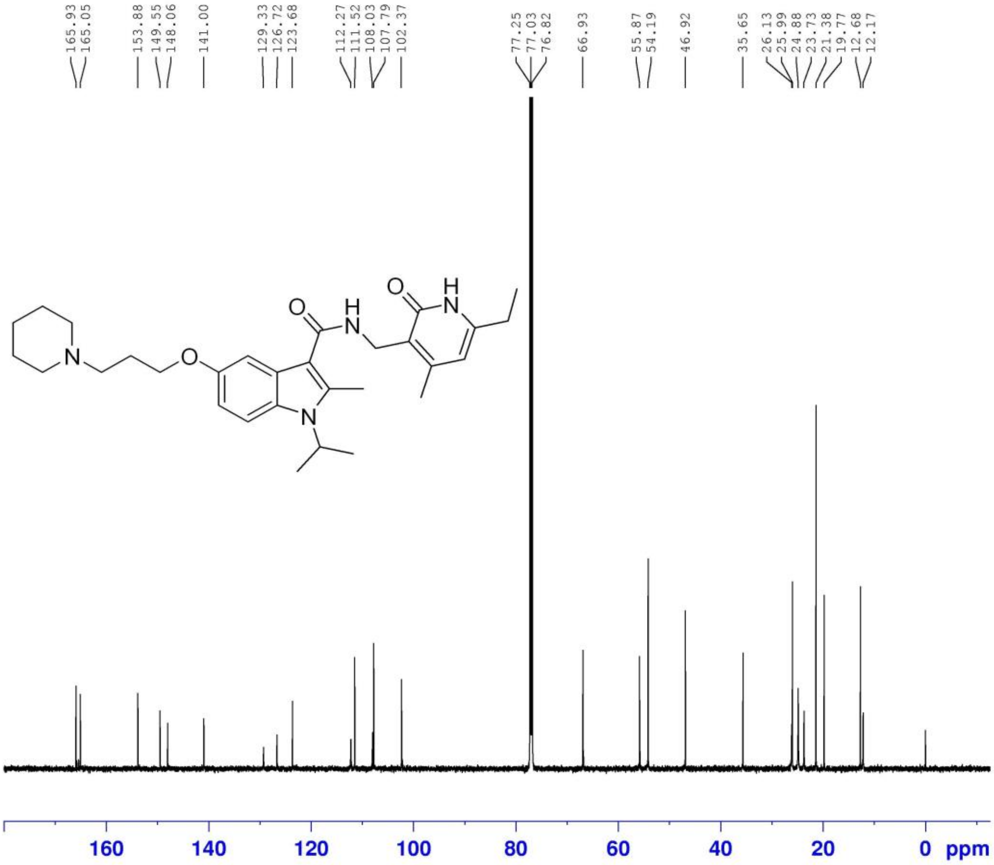
165.93
165.05
153.88
149.55
148.06
141.00
129.33
126.72
123.68
112.27
111.52
108.03
107.79
102.37
77.25
77.03
76.82
66.93
55.87
54.19
46.92
35.65
26.13
25.99
24.88
23.73
21.38
19.77
12.68
12.17
160 140 120 100 80 60 40 20 0 ppm

Spectrum of L-20

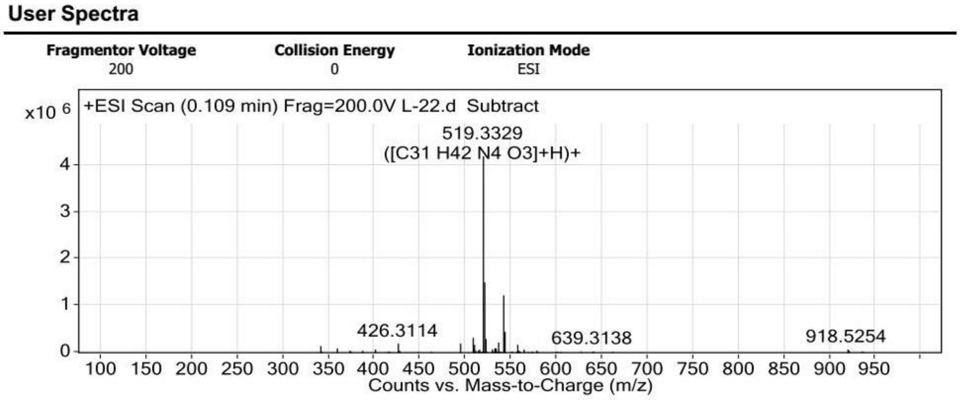

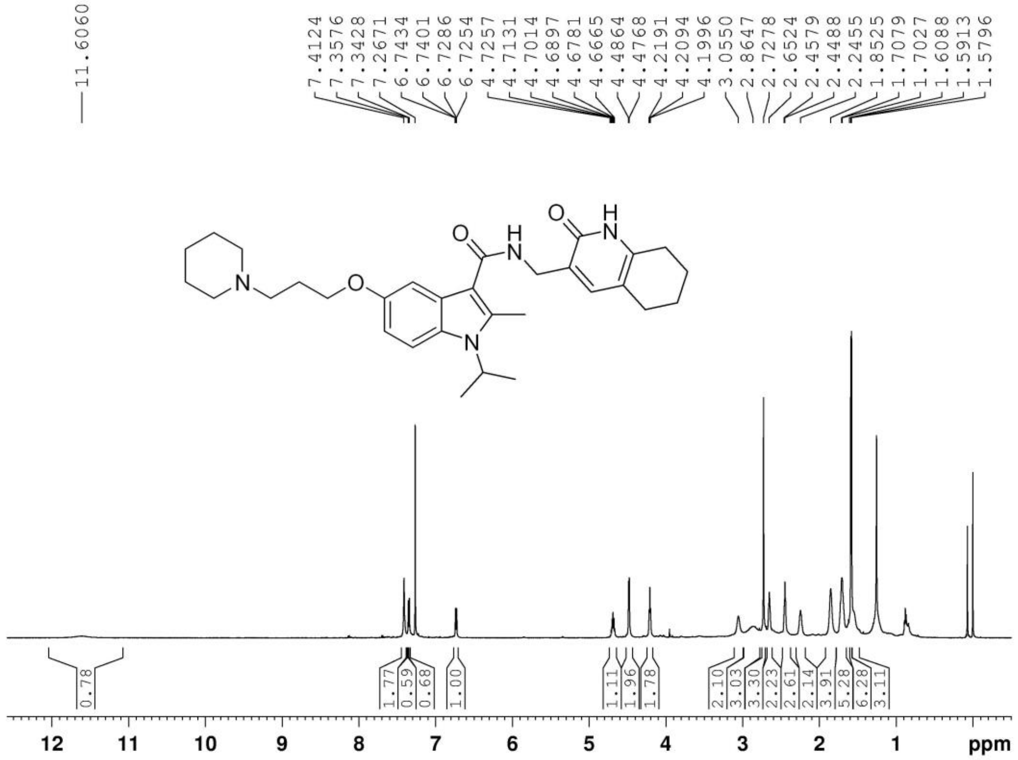

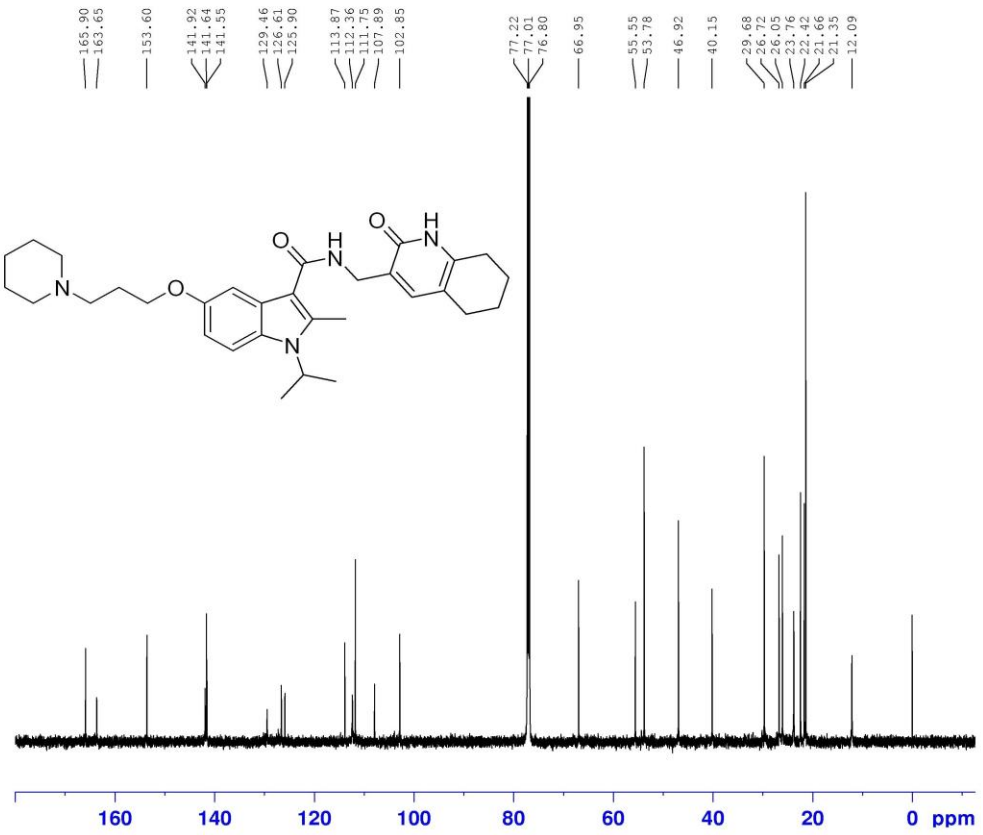

Spectrum of L-21

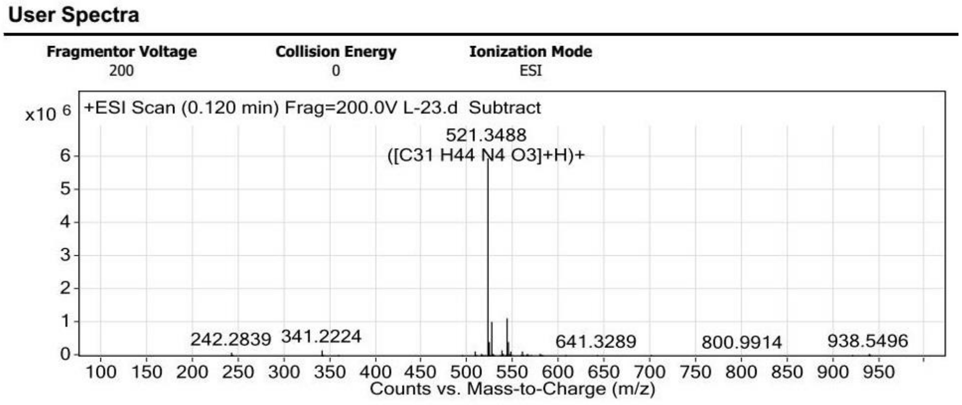

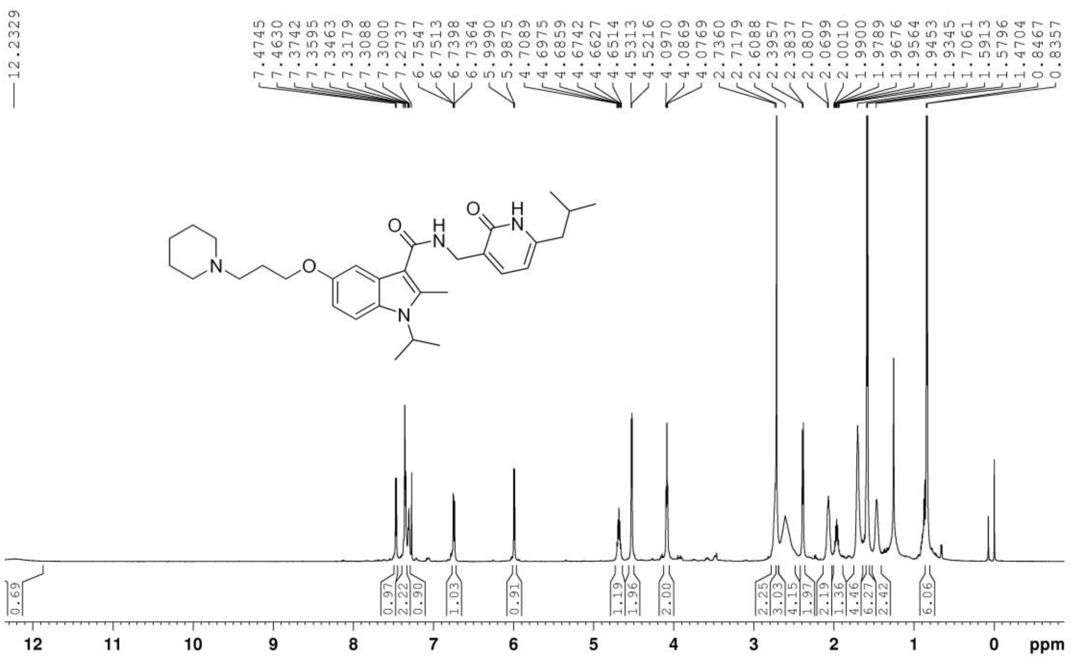
12.2329
7.4745 7.4630 7.3742 7.3595 7.3463 7.3179 7.3088 7.3000 7.2737 6.7547 6.7513 6.7398 6.7364 5.9990 5.9875 4.7089 4.6975 4.6859 4.6742 4.6627 4.6514 4.5313 4.5216 4.0970 4.0869 4.0769 2.7360 2.7179 2.6088 2.3957 2.3837 2.0807 2.0699 2.0010 1.9900 1.9789 1.9676 1.9564 1.9453 1.9345 1.7061 1.5913 1.5796 1.4704 0.8467 0.8357
0.69 0.97 2.22 0.90 1.03 0.91 1.19 1.96 2.00 2.25 3.03 4.15 1.97 2.19 1.36 4.46 6.27 2.42 6.06
12 11 10 9 8 7 6 5 4 3 2 1 0 ppm

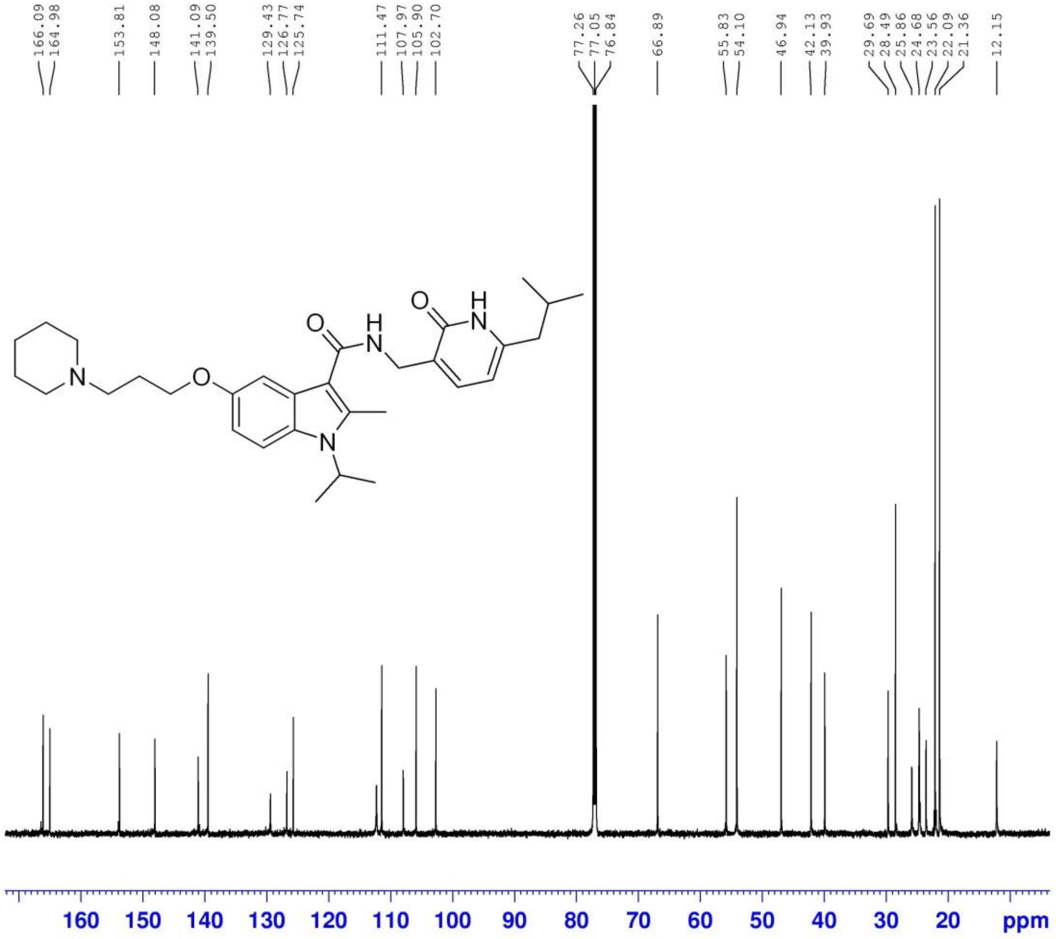
166.09 164.98 153.81 148.08 141.09 139.50 129.43 126.77 125.74 111.47 107.97 105.90 102.70 77.26 77.05 76.84 66.89 55.83 54.10 46.94 42.13 39.93 29.69 28.49 25.86 24.68 23.56 22.09 21.36 12.15
160 150 140 130 120 110 100 90 80 70 60 50 40 30 20 ppm

Spectrum of L-22

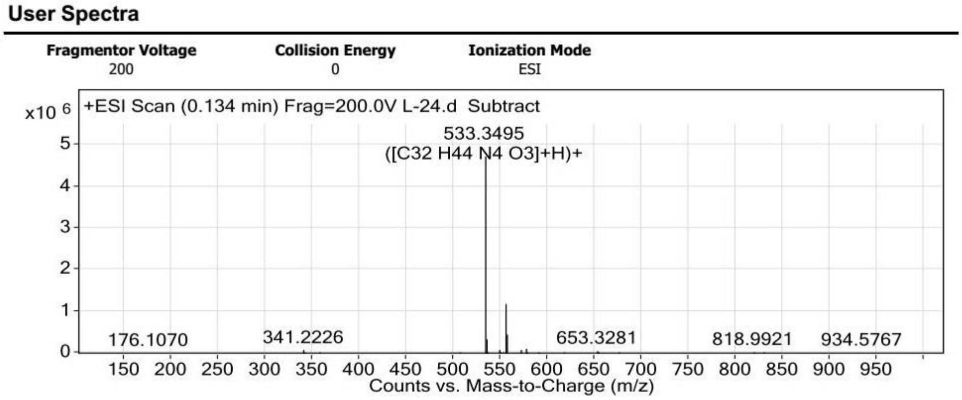

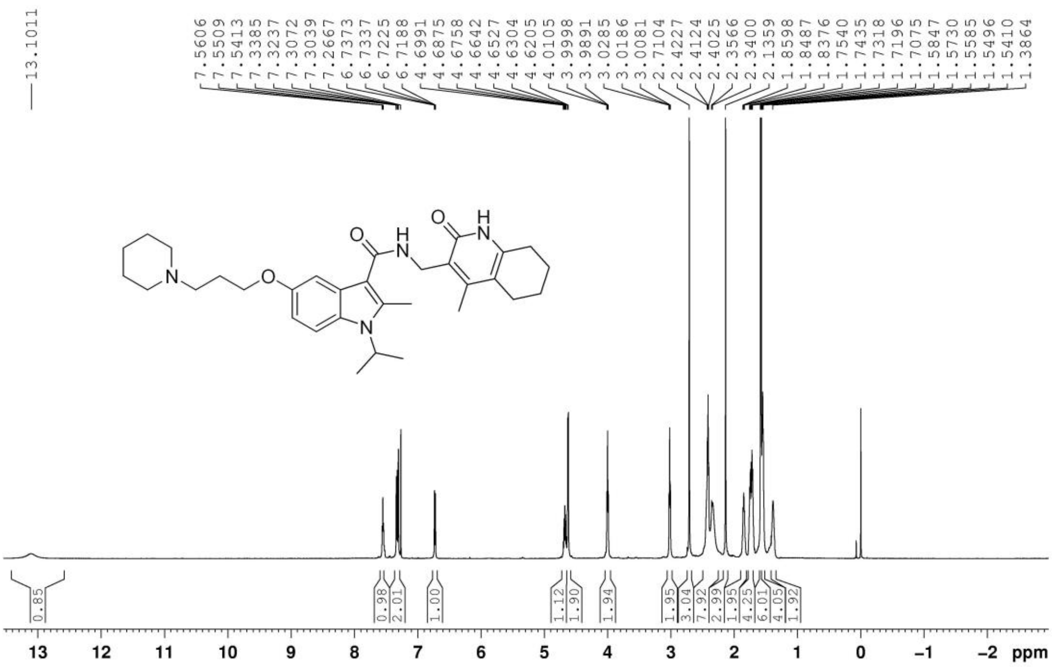

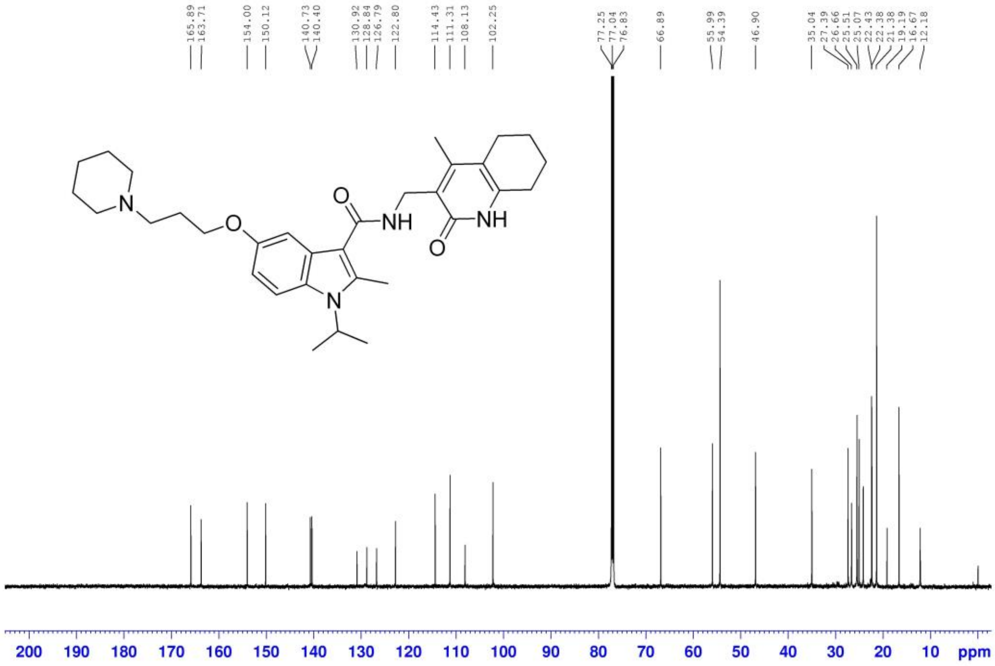
165.89
163.71
154.00
150.12
140.73
140.40
130.92
128.84
126.79
122.80
114.43
111.31
108.13
102.25
77.25
77.04
76.83
66.89
55.99
54.39
46.90
35.04
27.39
26.66
25.51
25.07
22.43
22.38
21.38
19.19
16.67
12.18
O
NH
NH
O
N
O
N
200 190 180 170 160 150 140 130 120 110 100 90 80 70 60 50 40 30 20 10 ppm
